# A Novel Signal Transduction Pathway that Modulates *rhl* Quorum Sensing and Bacterial Virulence in *Pseudomonas aeruginosa*

**DOI:** 10.1371/journal.ppat.1004340

**Published:** 2014-08-28

**Authors:** Qiao Cao, Yue Wang, Feifei Chen, Yongjie Xia, Jingyu Lou, Xue Zhang, Nana Yang, Xiaoxu Sun, Qin Zhang, Chao Zhuo, Xi Huang, Xin Deng, Cai-Guang Yang, Yan Ye, Jing Zhao, Min Wu, Lefu Lan

**Affiliations:** 1 Hainan University, Haikou, Hainan, China; 2 Shanghai Institute of Materia Medica, Chinese Academy of Sciences, Shanghai, China; 3 Institute of Chemistry and BioMedical Sciences, State Key Laboratory of Pharmaceutical Biotechnology, School of Life Sciences, Nanjing University, Nanjing, China; 4 State Key Laboratory of Respiratory Diseases and the First Affiliated Hospital of Guangzhou Medical College, Guangzhou, China; 5 Department of Chemistry and Institute for Biophysical Dynamics, The University of Chicago, Chicago, Illinois, United States of America; 6 Department of Basic Sciences, University of North Dakota School of Medicine and Health Sciences, Grand Forks, North Dakota, United States of America; The University of Texas at Austin, United States of America

## Abstract

The *rhl* quorum-sensing (QS) system plays critical roles in the pathogenesis of *P. aeruginosa*. However, the regulatory effects that occur directly upstream of the *rhl* QS system are poorly understood. Here, we show that deletion of gene encoding for the two-component sensor BfmS leads to the activation of its cognate response regulator BfmR, which in turn directly binds to the promoter and decreases the expression of the *rhlR* gene that encodes the QS regulator RhlR, causing the inhibition of the *rhl* QS system. In the absence of *bfmS*, the Acka-Pta pathway can modulate the regulatory activity of BfmR. In addition, BfmS tunes the expression of 202 genes that comprise 3.6% of the *P. aeruginosa* genome. We further demonstrate that deletion of *bfmS* causes substantially reduced virulence in lettuce leaf, reduced cytotoxicity, enhanced invasion, and reduced bacterial survival during acute mouse lung infection. Intriguingly, specific missense mutations, which occur naturally in the *bfmS* gene in *P. aeruginosa* cystic fibrosis (CF) isolates such as DK2 strains and RP73 strain, can produce BfmS variants (BfmS_L181P_, BfmS_L181P/E376Q_, and BfmS_R393H_) that no longer repress, but instead activate BfmR. As a result, BfmS variants, but not the wild-type BfmS, inhibit the *rhl* QS system. This study thus uncovers a previously unexplored signal transduction pathway, BfmS/BfmR/RhlR, for the regulation of *rhl* QS in *P. aeruginosa*. We propose that BfmRS TCS may have an important role in the regulation and evolution of *P. aeruginosa* virulence during chronic infection in CF lungs.

## Author Summary

The *rhl* quorum-sensing (QS) system allows *P. aeruginosa* to regulate diverse metabolic adaptations and virulence. However, how *rhl* QS system is regulated remains largely unknown. Here, we report that two-component sensor BfmS controls *rhl* QS system by repressing its cognate response regulator BfmR, which directly suppresses the expression of *rhl* QS regulator RhlR gene and reduces the production of QS signal molecule N-butanoyl-L-homoserine lactone (C4-HSL). We find that BfmS is critical to the ability of *P. aeruginosa* to modulate the expression of virulence-associated traits and adapt to the host. Intriguingly, although wild-type BfmS is a repressor of BfmR, naturally occurring missense mutation (L181P, L181P/E376Q, or R393H) can convert its function from a repressor to an activator of BfmR, leading to BfmR activation, which in turn reduces the level of *rhl* QS signal C4-HSL. These results, therefore, provide important and novel insight into the regulation and evolution of *P. aeruginosa* virulence.

## Introduction

*Pseudomonas aeruginosa* is an important opportunistic pathogen that accounts for 10% of all hospital-acquired infections [1], [2]. Most notably, *P. aeruginosa* is the leading cause of chronic pulmonary infections and mortality in cystic fibrosis (CF) patients [3]. The success of *P. aeruginosa* relies on the production and precise coordination of numerous virulence-associated factors such as lipopolysaccharide, flagella, type IV pili, exopolysaccharide alginate, toxins, proteases, lipases, pyocyanin, and rhamnolipids, which are primarily controlled by regulatory systems such as the quorum-sensing (QS) system and the two-component system (TCS) [4]–[12].

*P. aeruginosa* has two well-characterized acyl-homoserine lactone (acyl-HSL)- based QS systems, *las* (LasR-LasI) and *rhl* (RhlR-RhlI) [4]–[6], [8]–[10]. In addition, a third *Pseudomonas* quinolone signal (PQS) acts as a link between the *las* and *rhl* QS systems, although PQS is not involved in sensing cell density [4]–[6], [8]–[10]. The synthase of LasI catalyzes the synthesis of N-(3-oxododecanoyl) homoserine lactone (3O-C12-HSL), whereas RhlI catalyzes the synthesis of N-butyryl-homoserine lactone (C4-HSL), which induces their respective cognate transcriptional regulators LasR and RhlR, responsible for the activation of numerous QS-controlled genes [4]–[6], [8]–[10]. The transcriptional regulator LasR is generally considered to sit at the top of the QS hierarchy in *P. aeruginosa*. LasR/3O-C12-HSL activates the transcription of *rhlR*, and RhlR/C4-HSL activates the transcription of *rhlI* and various virulence-associated genes [4]–[6], [8]–[10]. However, RhlR is able to control the expression of LasR-specific factors independent of LasR [13]. 2-(2-hydroxylphenyl)-thiazole-4-carbaldehyde (IQS) could activate the *rhl* system in a LasR-independent manner [14]. Thus, the regulation of the *rhl* QS system is much more complicated than previously thought. So far, LasR and Vfr are the two transcriptional regulators known to regulate the expression of *rhlR* directly, other than RhlR itself [4]–[6], [8]–[10], [15].

Pathogenic bacteria, including *P. aeruginosa*, probes its surrounding environment constantly and makes appropriate decisions during infection [7], [11], [12], [16]–[18]. An important molecular device to achieve sampling of environmental signals is the two-component system (TCS) [12], [16]. Classically, two-component systems are composed of an inner membrane-bound sensor, which is able to detect an environmental stimulus, and a response regulator, which is phosphorylated by the sensor kinase and which, in turn, modulates the expression of genes necessary for the appropriate physiological response [16]. Approximately 130 genes encoding for TCS components have been identified in the genome of *P. aeruginosa* [1], [7], [11]. This provides *P. aeruginosa* with a sophisticated capability to regulate diverse metabolic adaptations, virulence and antibiotic resistance processes [7]. In fact, a large number of TCSs or TCS components, such as GacSA, PhoPQ, SadARS, RetS, and LadS have been described as having a key role during the infection process [7], [8], [11]. However, the direct links between TCS and QS remain poorly understood [6]–[8], [11], [19]–[22].

The observation that the expression of BfmRS TCS was dramatically up-regulated in the lungs of cystic fibrosis patients compared to *in vitro* growth intrigued us [23]. We sought to determine the roles of BfmRS in virulence regulation in *P. aeruginosa*. In this study, we showed that BfmRS TCS directly controls *rhl* QS system and modulates the ability of *P. aeruginosa* to adapt to the host. We demonstrate that BfmRS TCS may play important roles in the regulation and evolution of bacterial virulence during long-term bacterial adaptation to lungs afflicted with cystic fibrosis.

## Results

### BfmS positively controls *rhl* QS in *P. aeruginosa*

**Figure 1:**
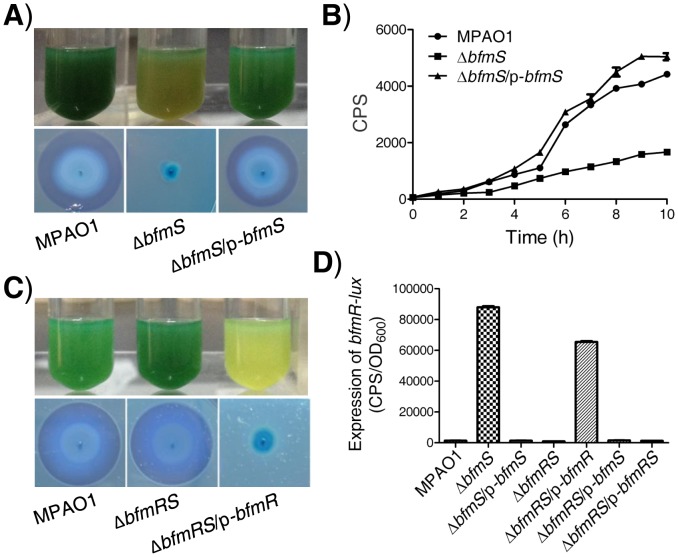
Effect of *bfmS* deletion on the production of virulence-associated factors and the promoter activity of *bfmR*. In all panels, MPAO1, Δ*bfmS*, and Δ*bfmRS* harbor plasmid PAK1900, respectively. **A**) Upper panel, *P. aeruginosa* MPAO1 and its derivatives were grown in Pyocyanin production broth (PPB) medium at 37°C for 36 h with shaking (250 rpm); the presence of the blue-green pigment indicates pyocyanin production. Lower panel, bacterial strains were inoculated onto a cetyltrimethylammonium bromide (CTAB) plate and incubated at 37°C for 24 h and then for 72 h at room temperature; the presence of a blue halo surrounding the colonies indicates the production of rhamnolipids. **B**) Relative amount of C4-HSL measured by the pDO100 (pKD-*rhlA*) system. MPAO1 and its derivatives were grown in M8-glutamate minimal medium supplemented with 0.2% glucose at 37°C for 24 h with shaking (250 rpm). Supernatants were subsequently prepared and measured for their relative C4-HSL contents. Plasmid pKD-*rhlA* carries the C4-HSL-responsive *rhlA* promoter fused to *luxCDABE*, so CPS (counts per second) values become an indirect measure of supernatant C4-HSL. **C**) Upper panel, MPAO1 and its derivatives were grown in PPB medium at 37°C for 24 h with shaking (250 rpm). Lower panel, MPAO1 and its derivatives were grown on CTAB plate and incubated at 37°C for 24 h and then for 72 h at room temperature. **D**) Expression of *bfmR-lux* in MPAO1 and its derivatives. Bacteria were grown in M8-glutamate minimal medium supplemented with 0.2% glucose at 37°C for 24 h with shaking (250 rpm) and then the *bfmR-lux* activity was measured. All experiments were independently repeated at least three times and the data shown represent comparable results. Values represent means ± standard error of the mean (SEM).

In *P. aeruginosa*, BfmS (PA4102) is a putative two-component sensor kinase with uncharacterized functions although its cognate response regulator BfmR (PA4101) has been reported to play an important role in biofilm maturation [24], [25]. To probe the biological roles of BfmS, we generated a *bfmS* null mutant strain (Δ*bfmS*) as described in the Materials and Methods section and in our previous studies [26]. Interestingly, Δ*bfmS* strain was defective in green pigment and rhamnolipids (Figure 1A), which can be complemented by introducing the native copy of *bfmS* (Table S1 in Text S1) into the Δ*bfmS* strain (Figure 1A). Quantitative analysis of pyocyanin and rhamnolipids indicates that the deletion of *bfmS* results in a 3.5-fold decrease of pyocyanin production and a 5-fold decrease in rhamnolipid production respectively (Figure S1A and S1B). Given that rhamnolipids promote the swarming motility of *P. aeruginosa* [27], we next examined the swarming motility of a wild-type MPAO1 strain, a Δ*bfmS* strain and its complementary strain (Δ*bfmS*/p-*bfmS*). As shown in Figure S1C, deletion of *bfmS* abolished swarming whereas both wild-type MPAO1 strain and the complementary strain swarmed on the surface of plates at 36 h.

The *rhlAB* operon is required for rhamnolipid synthesis [4]–[6], [9], [10]. We therefore constructed an *rhlA* promoter-*lux* fusion (*rhlA*-*lux*, Table S1 in Text S1) and measured its activity in a wild-type MPAO1 strain, a *bfmS* deletion strain (Δ*bfmS*), and its complementary strain (Δ*bfmS*/p-*bfmS*). The expression of *rhlA-lux* fusion in Δ*bfmS* was significantly lower than those of other strains when bacteria were grown in an M8-glutamate minimal medium supplemented with 0.2% glucose (Figure S1D). This result suggests that the decreased expression of *rhlAB* in Δ*bfmS* strain is likely responsible for the reduction in rhamnolipid production. Since the expression of *rhlAB* is positively controlled by the *rhl* quorum-sensing system in *P. aeruginosa* [4]–[6], [9], [10], we next sought to measure the RhlI-dependent autoinducer C4-HSL content in the wild-type MPAO1 strain, the Δ*bfmS* strain, and the complementary strain (Δ*bfmS*/p-*bfmS*). We used the pDO100 (pKD-*rhlA*) system [28] (Table S1 in Text S1) that carries a *lux* reporter fused with a *rhlA* promoter. As a result, supernatants prepared from either the wild-type MPAO1 strain or the complementary strain (Δ*bfmS*/p-*bfmS*), but not the Δ*bfmS* strain, markedly promoted the luminescence values and thereby C4-HSL levels (Figure 1B). We also observed that deletion of *bfmS* results in decreased *rhlI* promoter activity (Figure S1E). Based on these results, we conclude that BfmS positively controls *rhl* QS system in *P. aeruginosa*.

### Transcriptional profiling of the *bfmS* deletion mutant strain

To further study the roles of *bfmS*, we used microarray analysis in order to compare the transcriptome of the Δ*bfmS* strain with that of the wild-type MPAO1 strain. As a result, we identified 131 genes with increased transcript levels (≥2-fold) (Table S2 in Text S1) and 71 genes with decreased transcript levels (≤2-fold) (Table S3 in Text S1) in the Δ*bfmS* strain versus wild-type MPAO1 strain. These 202 genes represent ∼3.6% of the total number of annotated genes in the *P. aeruginosa* PAO1 genome. Of those 202 genes, 42% encode hypothetical proteins of unknown functions (Tables S2 and S3 in Text S1). Grouping these genes according to their annotated function shows that they belong to several functional categories, primarily transport of small molecules, carbon compound catabolism, translation, and adaptation (Tables S2 and S3 in Text S1).

Among the 131 genes whose expression is up-regulated in the Δ*bfmS* strain, 7 genes were up-regulated more than 10-fold. Interestingly, these 7 genes, including *PA4100*, *bfmR*, *PA4103*, *PA4104*, *PA4105*, *PA4106*, and *PA4107*, are located at or near the *bfmRS* (*PA4101*-*PA4102*) loci (Figure S2A, Table S2 in Text S1). These microarray-based expression data are consistent with the operon predictions for *P. aeruginosa*, which suggested that *PA4103* and *PA4104* are organized into *PA4103* operon (*PA4103*-*PA4104*) while *PA4105*, *PA4106* and *PA4107* are organized into *PA4107* operon (*PA4107*-*PA4106*-*PA4105*) (www.pseudomonas.com). Among these genes, *PA4100* encodes a dehydrogenase of unknown function, and *bfmR* encodes a two-component response regulator that acts as a biofilm maturation regulator, whereas *PA4103*, *PA4104*, *PA4105*, *PA4106*, and *PA4107* encode hypothetical proteins. Although PA4103 contains a ferric reductase like transmembrane component (pfam01794) and PA4107 contains a calcium binding motif (cd00051), their biological functions are unknown. Further characterization of the functions of these genes may provide insight into the roles of *bfmS* in *P. aeruginosa*.

There are 11 genes whose expressions are down-regulated more than 10-fold in Δ*bfmS* strain (Figure S2, Table S3 in Text S1). Among them, 5 genes (*rhlA*, *rhlB*, *antA*, *antB*, and *antC*) are already known to be controlled by the *rhl* QS system [29], [30]. In addition, we observed that deletion of *bfmS* decreases the transcription of *rhlI* by approximately 66% (Table S3 in Text S1). We also found a moderate, 20% decrease in *rhlR* transcription in the Δ*bfmS* mutant compared with the parent, which is consistent with the results of an *rhlR-lux* reporter gene analysis (Figure S3A). These results further suggest that *bfmS* positively controls the *rhl* QS. To further confirm the differentially expressed genes identified by the microarray analysis, 12 genes representing a range of microarray signal intensity and expression profiles were subjected to real-time (RT) PCR analyses. There was a high degree of consistency among data generated by these two methods (Table S4 in Text S1), which assures the reliability of microarray analysis in determining transcriptional changes.

### BfmS negatively controls BfmR, which is positively auto-regulated

Since deletion of *bfmS* led to the over-expression of *bfmR* (>90-fold) (Figure S2A, Table S2 in Text S1), we next sought to determine if the elevated *bfmR* contributes to the phenotypes observed in the Δ*bfmS* strain. We generated a *bfmRS* double mutant strain (Δ*bfmRS*) (Table S1 in Text S1) and performed phenotypic analysis. Interestingly, the Δ*bfmRS* strain and wild-type MPAO1 strain display similar phenotypes when bacteria are grown in Pyocyanin production broth (PPB) or on a cetyltrimethylammonium bromide (CTAB plate) (Figure 1C). The introduction of a wild-type *bfmR* gene (p-*bfmR*, Table S1 in Text S1) into Δ*bfmRS* strain restored its phenotypes similar to Δ*bfmS* strain (Figure 1C). These results suggest that the effect of *bfmS* deletion on either the pigment production or rhamnolipids production in *P. aeruginosa* is likely mediated through the over-expression of *bfmR*. Moreover, qRT-PCR analysis indicates that the expressions of at least 12 genes were significantly affected by the deletion of *bfmS*, whereas their altered expression levels caused by *bfmS* deletion were suppressed by additional deletion of *bfmR*. Thus, *bfmR* may mediate most, if not all, the output of *bfmS*.

We next tested if BfmS could affect the expression of BfmR. We constructed a *bfmR* promoter-*lux* fusion (*bfmR*-*lux*, Table S1 in Text S1) and then measured its activity in a wild-type MPAO1 strain, a *bfmS* deletion strain (Δ*bfmS*), its complementary strain (Δ*bfmS*/p-*bfmS*), a *bfmRS* double deletion strain (Δ*bfmRS*), and a Δ*bfmRS* strain harboring p-*bfmR*, p-*bfmS* or p-*bfmRS* (Table S1 in Text S1). As shown in Figure 1D, the activity of *bfmR*-*lux* in Δ*bfmS* strain was about 60-fold higher than that of the wild-type MPAO1 strain. Complementation with p-*bfmS* in the Δ*bfmS* strain restored the activity of *bfmR*-*lux* to levels similar to the wild-type strain (Figure 1D). In addition, the activity of *bfmR*-*lux* in the Δ*bfmRS* strain was similar to that observed in the wild-type MPAO1 strain; however, the introduction of p-*bfmR*, but not p-*bfmS* or p-*bfmRS*, to the Δ*bfmRS* strain dramatically increased the activity of *bfmR*-*lux* (>46-fold) (Figure 1D). Hence, BfmR can activate its own gene promoter in the absence of BfmS.

We next evaluated if the absence of BfmS causes an accumulation of BfmR in *P. aeruginosa*. To this end, we constructed an integration vector mini-ctx-BfmR-Flag (Table S1 in Text S1) and the resulting clone was mobilized into the wild-type MPAO1 and Δ*bfmRS* strain, yielding an MPAO1::*BfmR*-*Flag* strain and Δ*bfmRS*::*BfmR*-*Flag* strain, respectively. The Δ*bfmRS*::*BfmR*-*Flag* strain displayed a pigment-deficient phenotype as observed for either the Δ*bfmS* strain or the Δ*bfmRS*/p-*bfmR* strain (Figure S4A). The cell lysates of MPAO1::*BfmR*-*Flag* strain, Δ*bfmRS*::*BfmR*-*Flag* strain and its complementary strain (Δ*bfmRS*::*BfmR*-*Flag*/p-*bfmS*) were subjected to Western blot analysis using anti-FLAG antibodies. As shown in Figure S4B, a large amount of BfmR-Flag in the Δ*bfmRS*::*BfmR*-*Flag* strain was detected. In contrast, no detectable signal was obtained for the BfmR-Flag generated from the MPAO1::*BfmR*-*Flag* strain or the complementary strain (Δ*bfmRS*::*BfmR*-*Flag*/p-*bfmS*) (Figure S4B). Therefore, the absence of BfmS leads to an accumulation of its cognate response regulator BfmR.

### BfmR directly binds to the promoters of its own, *PA4107*, *PA4103*, and *rhlR*

**Figure 2:**
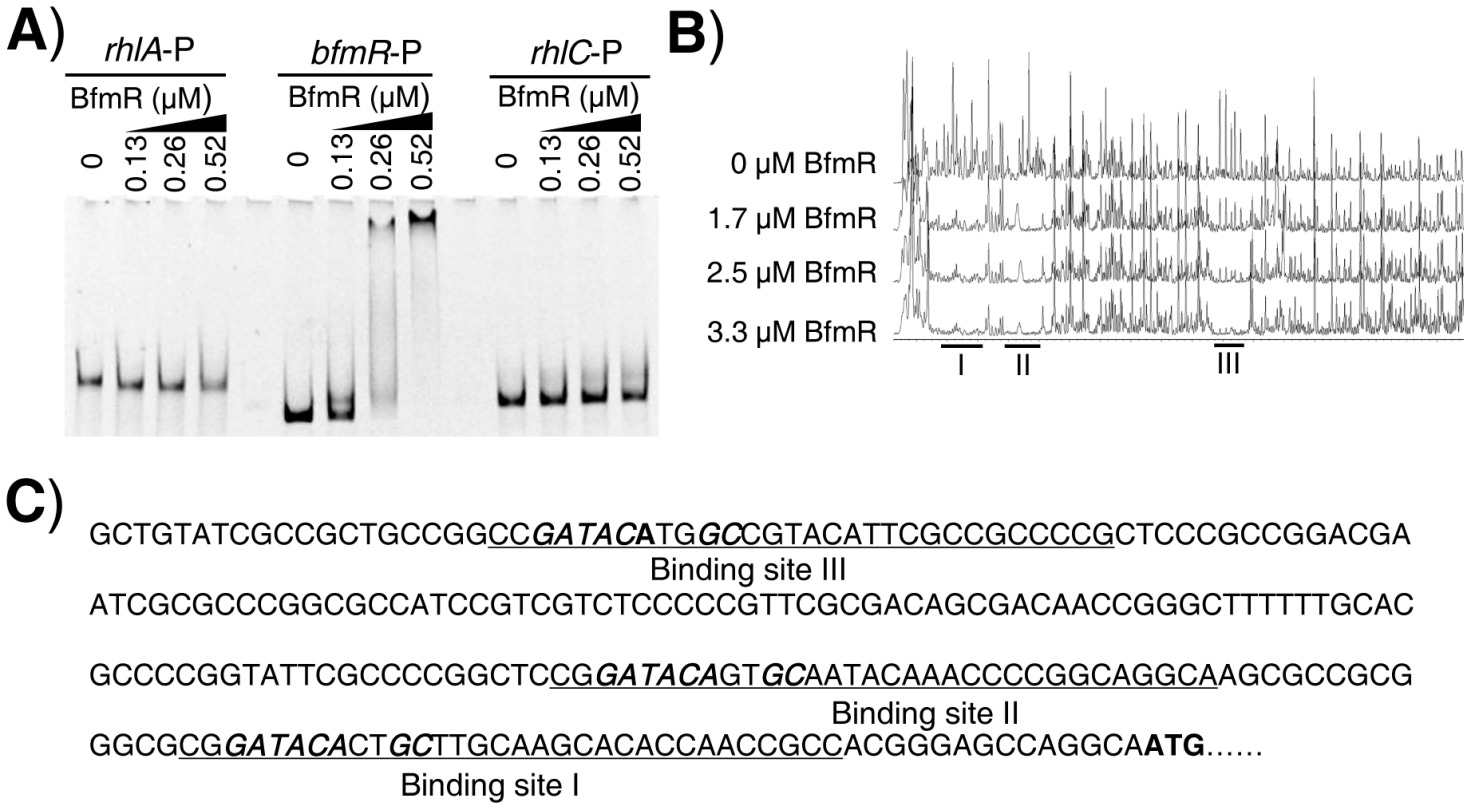
Direct binding of BfmR to its own promoter. **A**) EMSA showing that 6His-BfmR binds to its own promoter but not to the promoter of either *rhlA* or *rhlC*. **B**) Dye primer-based DNase I footprint assays show the protection pattern of the *bfmR* promoter after digestion with DNase I following incubation in the absence or presence of different amounts of 6His-BfmR, as indicated. Three BfmR-protected regions in the *bfmR* promoter DNA were demonstrated. **C**) *bfmR* promoter sequence with a summary of the DNase I footprint assay results. The BfmR-protected regions are underlined, and the putative BfmR box is in bold and in italics. Start codon of *bfmR* is in bold.

Since BfmR activates its own gene promoter, we next aimed to test if BfmR could bind its own promoter. We performed electrophoretic mobility shift assay (EMSA) using 6His-BfmR protein and DNA fragments containing *bfmR*, *rhlA*, and *rhlC* promoter regions, respectively. We found that 6His-BfmR could shift the *bfmR* promoter DNA, although it failed to shift the *rhlA* or *rhlC* promoter DNA (Figure 2A). We further determined the specific DNA sequence that BfmR can recognize in the *bfmR* promoter region by using a dye-primer-based DNase I footprint assay. We uncovered three BfmR-protected regions in the *bfmR* promoter DNA (Figure 2B). Interestingly, all three BfmR-protected regions contain a consensus sequence GATACAnnGC (where n is any nucleotides, Figure 2C).

Using RAST (http://rsat.ulb.ac.be/rsat/), we found that 41 promoters (−1 bp to −400 bp of the coding region) of *P. aeruginosa* PAO1, including the *PA4017* promoter, contain a putative BfmR-binding motif (GATACAnnGC) (Table S5 in Text S1). As expected, BfmR could shift the *PA4107* promoter DNA (Figure S5A) in our EMSA analysis, although it failed to shift the *rhlI* promoter DNA (Figure S5A). Interestingly, we also observed that BfmR is able to bind to *PA4103* promoter DNA (Figure S5B) that lacks a canonical BfmR-binding motif (GATACAnnGC) (Table S5 in Text S1). Using a dye-primer-based DNase I footprint assay, we uncovered that the BfmR-protected region of *PA4103* promoter DNA contains a non-canonical BfmR-binding motif (GATACAnnAC, the mismatch is underlined) (Figure S5C), which is subsequently determined to be required for the BfmS-mediated regulation of *PA4103* promoter activity (Figure S5D). Thus, it is likely that BfmR directly control the expression of the *PA4107* and *PA4103* operon.

**Figure 3:**
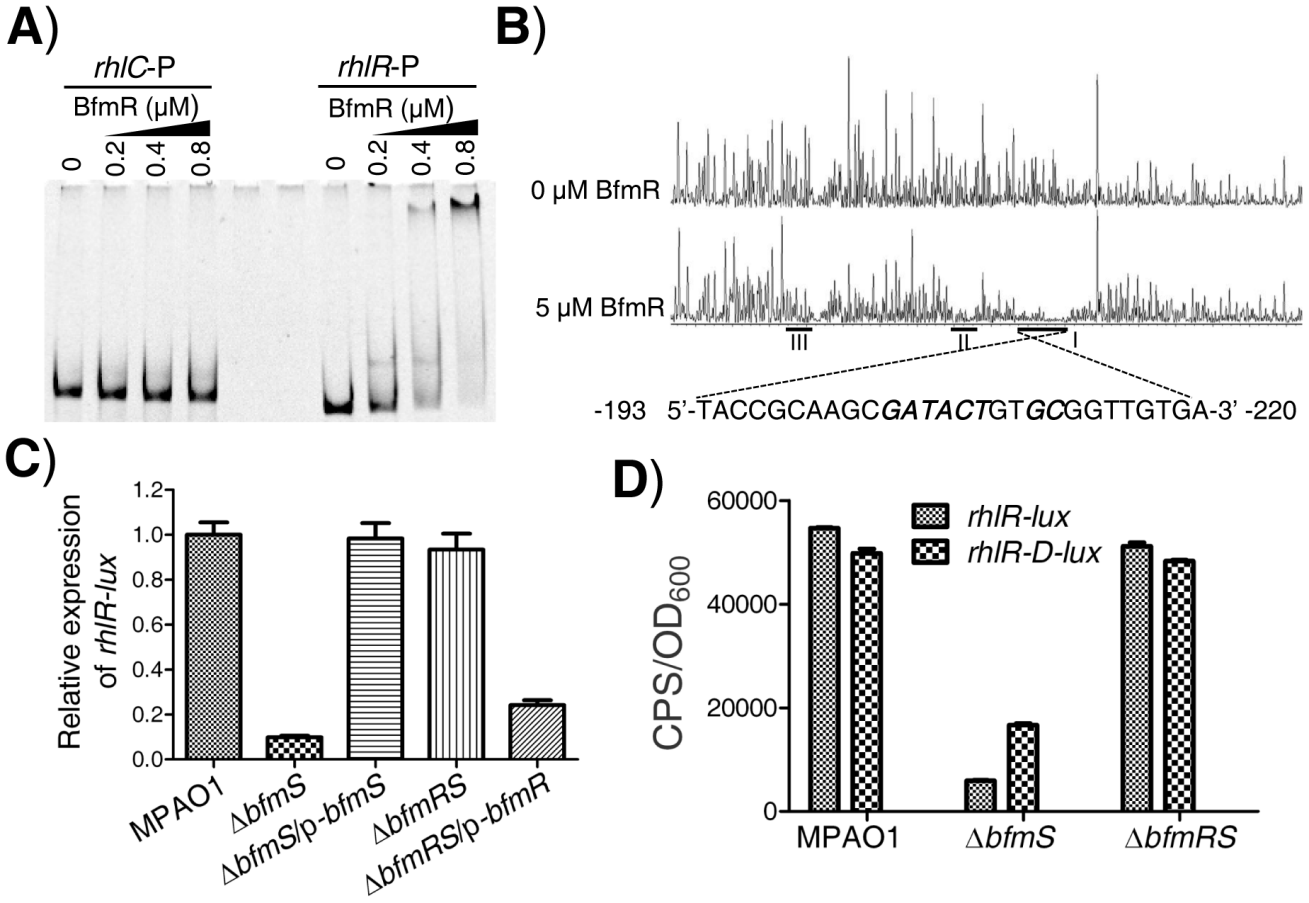
BfmR regulates the expression of *rhlR* in a direct manner. **A**) EMSA shows that 6His-BfmR directly binds to the promoter DNA of *rhlR* but not to that of *rhlC*. **B**) Electropherograms show the protection pattern of the *rhlR* promoter after digestion with DNase I following incubation in the absence or the presence of 6His-BfmR. There are three BfmR-protected regions (I, II, and III) in the promoter of the *rhlR*. BfmR-protected regions I harbors a putative BfmR-binding motif, which was highlighted in bold and in italics, as indicated. **C**) The relative expression of *rhlR-lux* in the wild-type MPAO1 (harboring PAK1900), the Δ*bfmS* strain (harboring PAK1900), the Δ*bfmS*/p-*bfmS* strain, the Δ*bfmRS* strain (harboring PAK1900), and the Δ*bfmRS*/p-*bfmR* strain. The relative gene expression in the wild-type MPAO1 was set to 1, and the other values were adjusted accordingly. **D**) The expression of *rhlR-lux* and *rhlR-D-lux* in the wild-type MPAO1, the Δ*bfmS* strain and the Δ*bfmRS* strain, as indicated. Bacteria were grown in low phosphate (0.32 mM) M8-glutamate minimal medium supplemented with 2% glucose at 37°C for 48 h. Values represent means ± SEM. The assays were independently repeated at least three times and the data shown represent comparable results.

As aforementioned, BfmR negatively controls the *rhl* QS system of *P. aeruginosa*, which does not need to bind promoter of *rhlI* (Figure S5A and S5B). These observations prompted us to determine if BfmR binds to the promoter of *rhlR*. EMSA analysis indicated that 6His-BfmR bound to the *rhlR* promoter DNA but not to the promoter region of *rhlC* that serves as a negative control (Figure 3A). Dye-primer-based DNase I footprint assay indicated that there were three BfmR-protected regions in the promoter of the *rhlR* (Figure 3B). Interestingly, protected region I (−220 to −193 from the start codon of *rhlR*) harbored a putative BfmR-binding motif (GATACTnnGC) with one mismatch (underlined) (Figure 3B), oriented in the opposite direction of the transcription of *rhlR*. Protected region II extends from nucleotide −151 to nucleotide −171 while protected region III extends from nucleotide −69 to nucleotide −85, relative to the start codon of *rhlR*, respectively (Figure 3B).

There are 44 *bfmS*-regulated genes harbor a consensus sequence (GATACAnnGC with or without one mismatch) in their promoter region (−1 bp to −400 bp of the coding region) (Tables S2 and S3 in Text S1). Additionally, there are 984 promoters (−1 bp to −400 bp of the coding region) harbor the consensus sequence (GATACAnnGC without or with one mismatch) in the PAO1 genome. These observations suggest that BfmR may serves as a global regulator affecting expression of a large number of genes.

### BfmR represses the expression of *rhlR* in the absence of BfmS

We next elucidated if BfmR regulates the expression of *rhlR*. To do this, we measured *rhlR* promoter-*lux* fusion activity in the wild-type MPAO1 strain, the Δ*bfmS* strain, the complementary strain (Δ*bfmS*/p-*bfmS*), the Δ*bfmRS* strain, and the Δ*bfmRS* strain harboring p-*bfmR* (Δ*bfmRS*/p-*bfmR*). The low-phosphate (0.32 mM Pi) M8-glutamate minimal medium supplemented with 0.2% glucose, used as phosphate limitation, served to stimulate the expression of *rhlR* [21]. As shown in Figure 3C and S3B, the activity of *rhlR*-*lux* in the Δ*bfmS* strain was more than 8-fold lower than that observed in the wild-type MPAO1 strain. Complementation with p-*bfmS* in the Δ*bfmS* strain restored the activity of *rhlR*-*lux* similar to that of the wild-type strain (Figure 3C). Moreover, Δ*bfmRS* strain exhibited *rhlR*-*lux* activity similar to that observed in the wild-type MPAO1 strain, while the introduction of p-*bfmR* into the Δ*bfmRS* strain caused a 3.8-fold decrease in *rhlR*-*lux* activity (Figure 3C). Thus, it is likely that *bfmS* activates the expression of *rhlR* by repressing BfmR, which acts to negatively regulate *rhlR* expression and *rhl* QS. This notion was further substantiated by the observations that under low-phosphate growth conditions Δ*bfmS* also exhibits decreased *rhlI* (Figure S3C) and *rhlA* (Figure S3D) promoter activity, and lowered C4-HSL content (Figure S3E) as compared to wild-type MPAO1.

To determine if the putative BfmR-binding motif (GATACTnnGC) (Figure 3B) is involved in the BfmR-mediated inhibition of *rhlR-lux* activity, we deleted the first five residues (GATACT) in the consensus sequence (yielding *rhlR-D-lux*, Table S1 in Text S1), and examined the ability of the mutant sequence to permit the inhibition of the reporter gene in Δ*bfmS* strain. As shown in Figure 3D, the *rhlR*-*lux* activity in Δ*bfmS* strain was approximately 8-fold lower than that observed in the wild-type MPAO1 strain or in the Δ*bfmRS* strain. However, the *rhlR-D-lux* activity was about 2-fold lower in the Δ*bfmS* strain compared to the wild-type MPAO1 strain or the Δ*bfmRS* strain (Figure 3D). Thus, the five residues (GATACT) are required for the full inhibition of *rhlR-lux* activity in Δ*bfmS* strain, demonstrating the importance of these conserved binding-site residues. However, besides GATACT sequence elements, additional regulatory sequence elements within the promoter region of *rhlR* are most likely involved in BfmR-mediated inhibition of *rhlR-lux* activity, given that the *rhlR-D-lux* activity in Δ*bfmS* strain is still decreased, although to a much lesser extent than that of *rhlR-lux* (Figure 3D).

### The Pta-AckA pathway modulates the activation of BfmR

**Figure 4:**
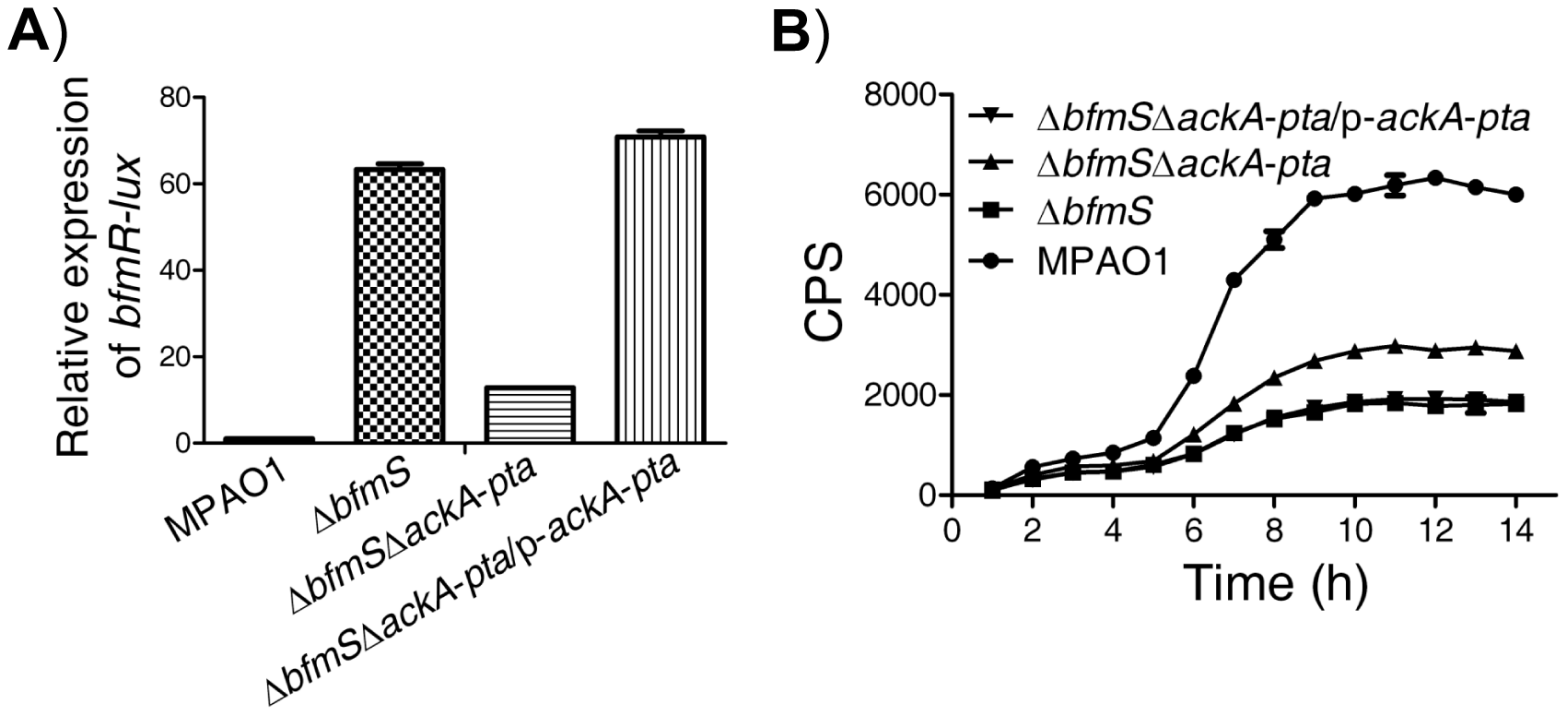
Effect of the Pta-AckA pathway and carbon sources availability on the activation of BfmR in the absence of BfmS. In all panels, MPAO1, Δ*bfmS*, and Δ*bfmS*Δ*ackA*-*pta* harbor plasmid PAK1900, respectively. **A**) The relative expression of *bfmR-lux* in the wild-type MPAO1, the Δ*bfmS* strain, the Δ*bfmS*Δ*ackA*-*pta* strain, and its complementary strain (Δ*bfmS*Δ*ackA*-*pta*/p-*ackA-pta*) when bacteria were grown in M8-glutamate minimal medium supplemented with 0.2% glucose at 37°C for 24 h with shaking (250 rpm). Values are relative to MPAO1 (set to 1). **B**) Relative amount of C4-HSL measured by the pDO100 (pKD-*rhlA*) system. MPAO1 and its derivatives were grown in M8-glutamate minimal medium supplemented with 0.2% glucose at 37°C for 24 h with shaking (250 rpm). Supernatants were subsequently prepared and measured for the relative C4-HSL contents. Values represent means ± SEM. The assays were independently repeated at least three times with similar results obtained, and the graphs show a set of representative data.

Like many other response regulators [31], BfmR can be phosphorylated by acetyl phosphate (Figure S6A) and hence activated *in vitro* (Figure S6B). We further observed that the predicted phosphorylation site, aspartate residue D55, is required for the activation of BfmR *in vitro* and *in vivo* (Figure S7). As acetyl∼P is an intermediate in the acetate kinase (AckA)-phosphate acetyltransferase (Pta) pathway [31], we hypothesized that the AckA-Pta pathway may be involved in the activation of BfmR. Thus, we constructed a mutant strain (Δ*bfmS*Δ*ackA*-*pta*, Table S1 in Text S1) with deletion of both the *bfmS* gene and *ackA*-*pta* operon and measured the activity of *bfmR-lux* as well as the C4-HSL content in this strain and the Δ*bfmS* strain, respectively. As shown in Figure 4, the expression of *bfmR-lux* was lower (Figure 4A) while the C4-HSL content was higher (Figure 4B) in the Δ*bfmS*Δ*ackA*-*pta* strain than that of the Δ*bfmS* strain, respectively. The introduction of wild-type *ackA*-*pta* operon (p-*ackA-pta*, Table S1 in Text S1) into the Δ*bfmS*Δ*ackA*-*pta* strain was able to restore either the activity of *bfmR-lux* (Figure 4A) or the C4-HSL content to the level of the Δ*bfmS* strain (Figure 4B). Therefore, in the Δ*bfmS* strain, acetyl phosphate or the component that is dependent on the Acka-Pta pathway is required for the full activity of BfmR.

### BfmS regulates bacterial virulence and the ability of *P. aeruginosa* to adapt to the host environment

**Figure 5:**
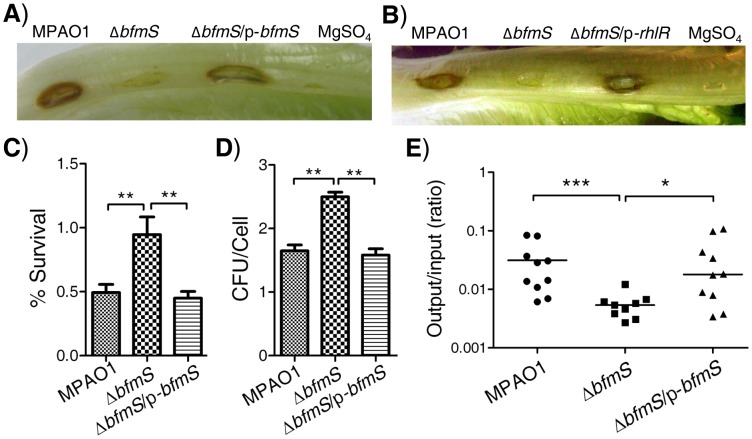
Effect of *bfmS* deletion on bacterial virulence and the ability of *P. aeruginosa* to adapt to the host. In all panels, MPAO1 and Δ*bfmS* harbor plasmid PAK1900, respectively. **A**) Photographs show lettuce midribs after three days of infection with 1×10^7^ cfu of *P. aeruginosa*. Wild-type MPAO1 strain or Δ*bfmS*/p-*bfmS* strain shows necrosis and tissue maceration of infection. The Δ*bfmS* strain shows weak signs of infection. **B**) Constitutive expression of *rhlR* in Δ*bfmS* strain could restore the virulence to the level of the wild-type MPAO1 strain. In **A**) and **B**), lettuce leaves were inoculated with 10 mM MgSO_4_ as a control. **C**) Effect of *P. aeruginosa* inoculum on the survival rate of murine lung epithelial cell line 12 (MLE-12). **D**) Internalization of *P. aeruginosa* into the murine lung epithelial cell line 12 (MLE-12). In **C**) and **D**), values represent means ± SEM. **E**) Recovery of *P. aeruginosa* derivatives in a mouse model of acute pneumonia. Results are expressed as the ratio of cfu recovered per lung (output) to cfu present in the initial inoculum (input) and represent results from n = 9–10 mice per strain; the line shows the geometric mean for each group. CFU, Colony-Forming Unit. The Mann–Whitney test was used to calculate p-values (two-tailed). * p<0.05, ** p<0.01, *** p<0.001. Results are representative of two independent experiments.

As BfmS modulates the *rhl* QS system that contributes significantly to the virulence of *P. aeruginosa* [4]–[6], [9], [10], we infected romaine lettuce leaves with *P. aeruginosa* to see if BfmS controls bacterial virulence. The pathogenicity assay revealed a significant difference in the manifestation of infection symptoms caused by the Δ*bfmS* strain compared to wild-type MPAO1 strain. Relative to wild-type MPAO1, the Δ*bfmS* strain failed to cause severe necrotic lesions of the leaves, which can be complemented by introducing the wild-type *bfmS* gene into the Δ*bfmS* strain (Figure 5A). In addition, the Δ*bfmRS* strain exhibited a virulence phenotype similar to that of a wild-type MPAO1 strain, while the introduction of p-*bfmR* into the Δ*bfmRS* strain led to a low virulence phenotype (Figure S8). Moreover, the constitutive expression of *rhlR* in Δ*bfmS* strain could restore either the virulence of Δ*bfmS* strain to the level of the wild-type MPAO1 strain (Figure 5B), suggesting that the decreased expression of *rhlR* is likely responsible for the attenuated virulence of Δ*bfmS* strain in the lettuce leaf model of *P. aeruginosa* infection.

Since cytotoxicity and invasion of *P. aeruginosa* are useful traits for this pathogen [32], we further characterized BfmS to check if it regulates the cytotoxicity or the invasion of *P. aeruginosa* in a murine lung epithelial cell line (MLE-12), a widely used *in vitro* model for studying host-pathogen interactions [33]–[35]. Using an MTT assay, we found that about 50% MLE-12 cells were killed when challenged with wild-type MPAO1 strain; however, only 5% of MLE-12 cells were killed after inoculation with the Δ*bfmS* strain (Figure 5C). Using a colony forming unit (CFU) assay, we showed that deletion of *bfmS* significantly increases (p<0.01) the internalization of *P. aeruginosa* by approximately 50% (Figure 5D). Further, the invasive and cytotoxic phenotypes of Δ*bfmS* strain could be completely restored to the wild-type levels by the introduction of p-*bfmS* (Figure 5C and 5D). Thus, deletion of *bfmS* causes a loss of cytotoxic capacity while it enhances the invasion of *P. aeruginosa* MPAO1 to MLE-12 cells.

To further determine the virulence regulated by BfmS, a mouse model of acute pneumonia was used as described in our previous study [26]. C57BL/6J mice were intranasally infected with approximately 5×10^6^ CFU of wild-type MPAO1, *bfmS* null mutant Δ*bfmS*, and its complementary strain Δ*bfmS*/p-*bfm*S. Figure 5E shows the CFU of bacteria recovered from the lungs compared to the initial inoculum at 18 h post infection, with a geometric mean indicated for each group. In this assay, wild-type MPAO1 was recovered in numbers approximately at 3.13% of the inoculum dose from lungs with a result 5.6-fold higher than that (0.56%) of the Δ*bfmS* strain. Further, bacteria of complementary strain (Δ*bfmS*/p-*bfm*S) were recovered from lungs with 3.43%, similar to that of the wild-type MPAO1 strain (Figure 5E). These results indicate that deletion of *bfmS* decreases *P. aeruginosa* survival in the mouse lungs in this model and thus reduced virulence.

### Specific mutations in *bfmS* result in the activation of BfmR

**Figure 6:**
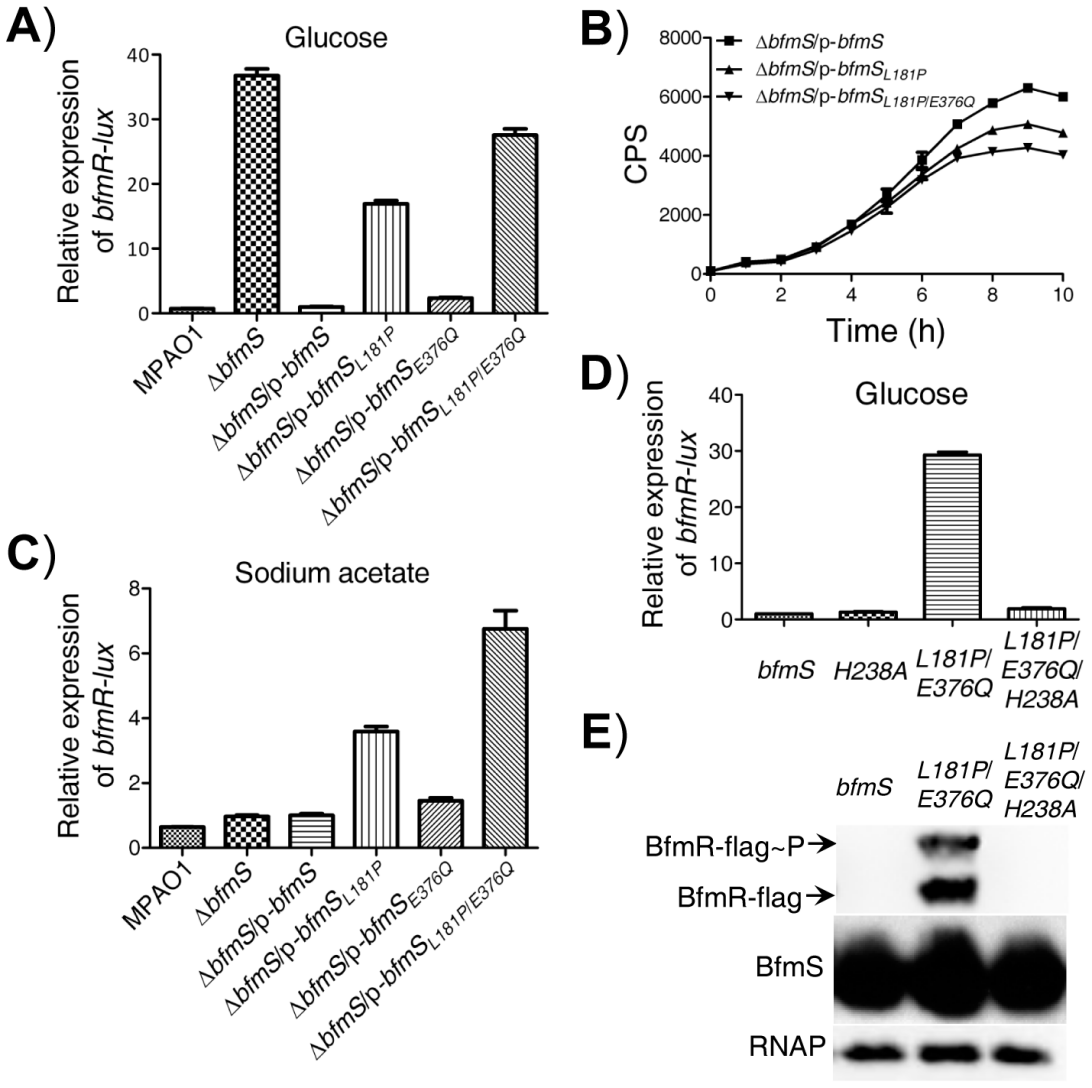
Effect of DK2 lineage-specific amino acid substitutions in BfmS on the activation of BfmR. In all panels, MPAO1 and Δ*bfmS* harbor plasmid PAK1900, respectively. **A**) The relative expression of *bfmR-lux* in the wild-type MPAO1 and its derivatives when bacteria were grown in M8-glutamate minimal medium supplemented with 0.2% glucose at 37°C for 36 h with shaking (250 rpm), as indicated. Values are relative to MPAO1 (set to 1). **B**) Relative amount of C4-HSL measured by the pDO100 (pKD-*rhlA*) system. MPAO1 and its derivatives were grown in M8-glutamate minimal medium supplemented with 0.2% glucose at 37°C for 36 h with shaking (250 rpm). Supernatants were subsequently prepared and measured for the relative C4-HSL contents. **C**) The relative expression of *bfmR-lux* in the wild-type MPAO1 and its derivatives when bacteria were grown in M8-glutamate minimal medium supplemented with 0.082% sodium acetate at 37°C for 36 h with shaking (250 rpm). Values are relative to MPAO1 (set to 1). **D**) The *bfmR-lux* activity in Δ*bfmS*/p-*bfmS* strain (*bfmS*), Δ*bfmS*/p-*bfmS_H238A_* strain (*H238A*), Δ*bfmS*/p-*bfmS_L181P/E376Q_* (*L181P/E376Q*), and Δ*bfmS*/p-*bfmS_L181P/E376Q/H238A_* strain (*L181P/E376Q/H238A*) when bacteria were grown in M8-glutamate minimal medium supplemented with 0.2% glucose at 37°C for 36 h with shaking (250 rpm). Values are relative to Δ*bfmS*/p-*bfmS* strain (set to 1). In **A**) to **D**), results are representative of three independent experiments and values represent means ± SEM. **E**) Phos-tag analysis shows that amino acid substitution L181P/E376Q, but not the L181P/E376Q/H238A, causes overproduction of phosphorylated and unphosphorylated BfmR. Western blot analysis of BfmS showing that missense mutations in *bfmS* do not affect the protein levels of BfmS, and immunoblots for RNAP (RNA polymerase) served as loading control. Cell lysates of Δ*bfmRS*::*BfmR*-*Flag*/p-*bfmS* strain (*bfmS*), Δ*bfmRS*::*BfmR*-*Flag*/p-*bfmS_L181P/E376Q_* (*L181P/E376Q*), and Δ*bfmRS*::*BfmR*-*Flag*/p-*bfmS_L181P/E376Q/H238A_* strain (*L181P/E376Q/H238A*) were used as described in Materials and Methods section.

The *P. aeruginosa* DK2 lineage is highly successful and has been isolated from ∼40 cystic fibrosis (CF) patients since the start of the sampling program in 1973 [36]. We noted that the DK2 lineage-specific mutations in BfmS are point mutations that cause two amino acid substitutions, proline replaces leucine 181 (L181P), and glutamine replaces glutamic acid (E376Q). Among them, L181P was fixed in the DK2 lineage before the year 1979, while E376Q was subsequently fixed in the DK2 lineage after 1991 [36]. We next investigated the regulatory effect associated with these amino acid substitutions observed in the BfmS. We created p-*bfmS_L181P_*, p-*bfmS_E376Q_*, and p-*bfmS_L181P_*_/*E376Q*_ plasmids (Table S1 in Text S1) and introduced them to the Δ*bfmS* strain, and tested their effects on *bfmR-lux* activity. Interestingly, L181P and E376Q substitutions in BfmS caused a 16-fold and a 2-fold increase in the activity of *bfmR-lux*, respectively (Figure 6A). More significantly, the combined substitution (L181P/E376Q) led to a 27-fold increase in the activity of *bfmR-lux* (Figure 6A). Accordingly, L181P or L181P/E376Q substitutions in BfmS decreased the RhlI-dependent autoinducer C4-HSL content (Figure 6B). These data strongly suggest that mutations in specific residues of BfmS result in activation of BfmR.

### Specific missense mutations convert BfmS function from a repressor to an activator of BfmR

The DK2 lineage-specific mutations (L181P, L181P/E376Q) may abolish the negative regulatory effects of BfmS on BfmR or alternatively, it may transform BfmS into a positive regulator of BfmR and therefore, activate BfmR (Figure 6A and 6B). To discriminate between these two possibilities, we tested the effect of these amino acid substitutions in BfmS on the activity of *bfmR-lux* when bacteria were grown in M8-glutamate minimal medium supplemented with sodium acetate. BfmS did not function as a negative regulator of BfmR when bacteria were grown in this media, given that the Δ*bfmS* strain exhibited similar *bfmR-lux* activity as the MPAO1 strain (Figure 6C). However, the Δ*bfmS*/p-*bfmS_L181P_* strain and Δ*bfmS*/p-*bfmS_L181P/E376Q_* strain displayed *bfmR-lux* activity 2.5-fold and 6-fold higher than that the activity observed in the reference strain (Δ*bfmS*/p-*bfmS*), respectively (Figure 6C). These results suggest that the L181P or L181P/E376Q amino acid substitution render BfmS as a positive regulator of BfmR. Further, the introduction of p-*bfmS_L181P_* or p-*bfmS_L181P/E376Q_* to the *bfmRS* double deletion mutant strain (Δ*bfmRS*) failed to increase the activity of *bfmR-lux* (Figure S9A), suggesting that the effect of L181P or L181P/E376Q substitutions in BfmS on the *bfmR-lux* activity is mediated through the activation of BfmR.

BfmS is a member of the HisKA subfamily of bacterial histidine kinases and it is predicted that the conserved H238 residue is required for its kinase activity [37]. We next investigated the regulatory role associated with this residue by changing His to Ala. We created p-*bfmS_H238A_* and p-*bfmS_L181P_*_/*E376Q*/*H238A*_ plasmids (Table S1 in Text S1) and introduced them to the Δ*bfmS* strain, and examined their effects on the *bfmR-lux* activity. As shown in Figure 6D, H238A substitution in BfmS has no significant effect on the activity of *bfmR-lux* when bacteria were grown in M8-glutamate minimal medium supplemented with 0.2% glucose. However, H238A substitutions in the BfmS_L181P/E376Q_ abolished its ability to induce the *bfmR-lux* activity (Figure 6D), which suggests that the H238 residue or the kinase activity is required for BfmS_L181P/E376Q_ to activate BfmR. This hypothesis is further supported by the fact that amino acid substitution L181P/E376Q, but not L181P/E376Q/H238A, causes overproduction of phosphorylated and unphosphorylated BfmR (Figure 6E).

Furthermore, we noted that the RP73-specific mutation in *bfmS* causes the substitution of arginine by histidine at the codon 393 (R393H) [38]. The *P. aeruginosa* strain RP73 was isolated after 16.9 years of chronic lung infection in a CF patient [38]. Like the L181P substitution or the L181P/E376Q substitution, the R393H substitution in BfmS resulted in increased *bfmR-lux* activity (Figure S9B and S9C) and decreased level of C4-HSL (Figure S9D), suggesting that BfmS_R393H_ activates BfmR. As the regulatory effects of these BfmS variants (BfmS_L181P_, BfmS_L181P/E376Q_, and BfmS_R393H_) on BfmR have been changed from negative to positive, we therefore term them “reverse function” mutants. Although we failed to obtain either the soluble full-length BfmS protein or the cytoplasmic region of BfmS that prevented us from the assays of the phosphatase/kinase activities of BfmS *in vitro*, our genetic analyses clearly indicate that specific missense mutations (L181P, L181P/E376Q, or R393H) can convert BfmS from a repressor to an activator of BfmR.

## Discussion

**Figure 7:**
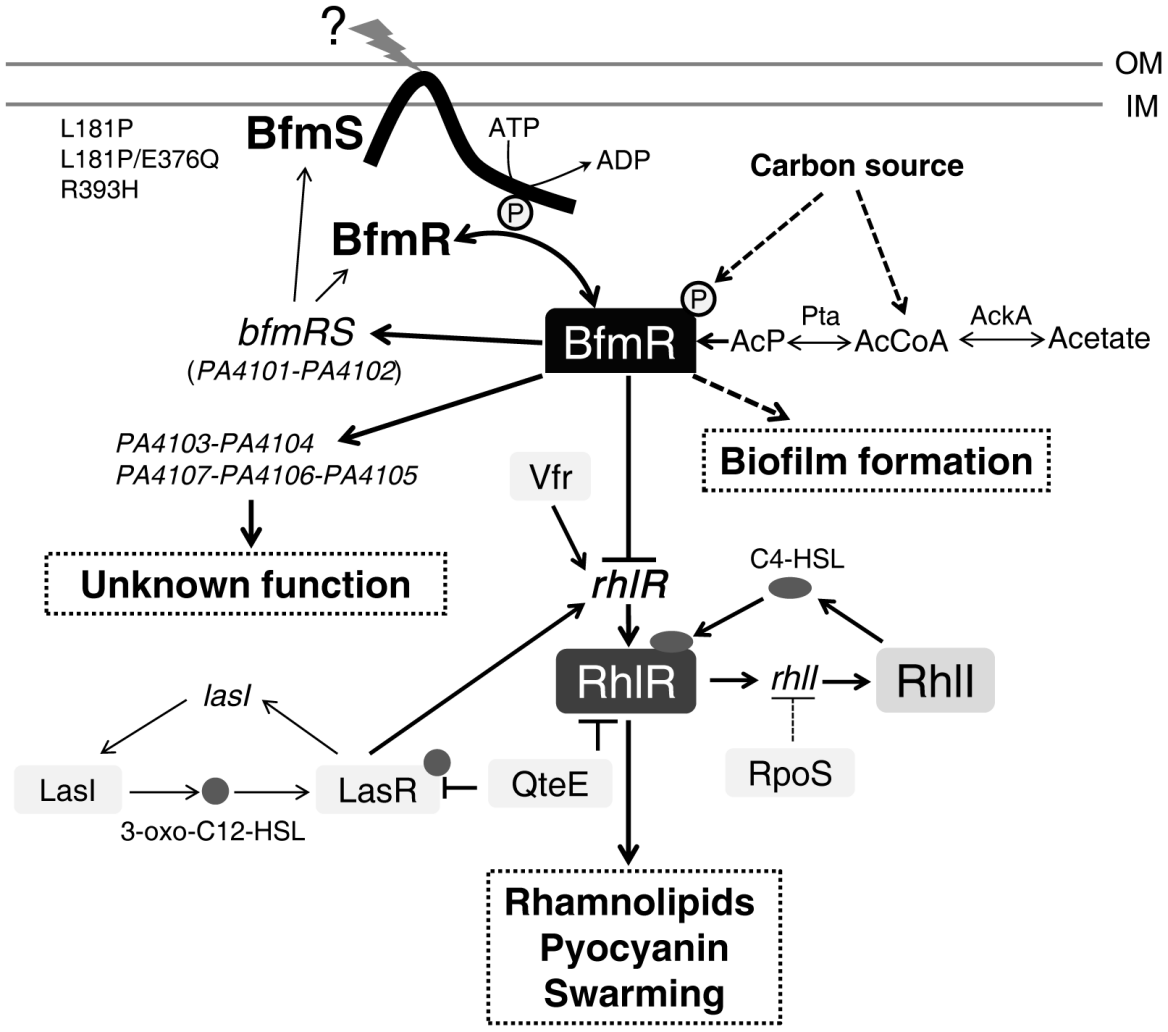
Model of the regulatory networks involving BfmRS in *P. aeruginosa*. The lines show the interaction between the players: arrow, activation; hammerheads, repression; solid line, a direct influence or direct connection; dotted line, a putative or indirect connection. The question mark (“?”) denotes a yet-unidentified factor (factors) required for triggering the kinase or phosphatase activity of BfmS. QteE reduces LasR protein stability [80]. QteE also blocks RhlR accumulation, and this effect is independent of QteE's action on LasR [80]. Inactivation of RpoS causes elevated levels of *rhlI* (but not *rhlR*) transcription [81]. See text for details. OM, outer membrane. IM, inner membrane. AcP, acetyl phosphate.

In this study, we uncovered a novel signal transduction pathway, BfmS/BfmR/RhlR, for the regulation of the *rhl* QS system in *P. aeruginosa*. We demonstrated that BfmS has profound effects on the expression of virulence-associated traits and the ability of *P. aeruginosa* to adapt to the host. In addition, we found that deletion of *bfmS* leads to a dramatic increase in biofilm formation and that BfmR mediates this effect (Figure S10). This appearance is consistent with previous observations that BfmR is a biofilm maturation regulator [24], [25]. Intriguingly, BfmS is able to switch its function from a repressor to an activator of BfmR in cystic fibrosis (CF) isolates such as DK2 strains and RP73 strain. A proposed model for signal transduction by BfmRS TCS is shown in Figure 7.

The prototypical two-component regulatory system is composed of a sensor kinase and a response regulator. In general, the sensor kinase senses an environment change and communicates it *via* phosphorylation to its cognate response regulator, and hence activates the response regulator's function. We demonstrated that BfmS functions as a negative regulator of BfmR (Figure 1D and Figure S4), whose activation requires phosphorylation on D55 (Figure S7). Therefore, BfmS might act as a phosphatase as opposed to a kinase for BfmR under our experimental conditions. In fact, many two-component sensors are bifunctional, catalyzing both the phosphorylation and dephosphorylation of their cognate response regulator [37], [39]–[41]. For some sensor kinases, the phosphatase activity may be the critical function *in vivo* [42]–[45]. Interestingly, the BfmS homologue in *Pseudomonas syringae*, RhpS, has also been shown to be a negative regulator of its cognate regulator RhpR in our previous studies [44], [45].

In the absence of BfmS, the Acka-Pta pathway can modulate the activity of BfmR (Figure 4). These observations suggest that the activation of BfmR is shaped by BfmS as well as by the nutritional status of *P. aeruginosa*. However, the expression of *bfmR-lux* in the Δ*bfmS*Δ*ackA*-*pta* strain was still about 12-fold higher than that in the wild-type MPAO1 strain (Figure 4A), indicating that acetyl phosphate or the component that is dependent on the Acka-Pta pathway is likely not the sole trigger of BfmR activation. This notion was further substantiated by the observation that the deletion of *ackA*-*pta* operon only partially alleviates the inhibition of QS signal C4-HSL production caused by the absence of *bfmS* (Figure 4B).

The *rhlR* gene encodes the transcriptional regulator RhlR, which has a central role in the quorum-sensing response, and is therefore very important for *P. aeruginosa* to co-ordinate its virulence in order to establish a successful infection [4], [6], [8]–[10], [46], [47]. We found that BfmR binds to and represses the *rhlR* promoter (Figure 3). A DNase I footprint analysis demonstrated that the BfmR-protected region (binding site I) of the *rhlR* promoter has a putative BfmR-binding motif (GATACTnnGC) that is crucial to the BfmR-mediated inhibition of *rhlR-lux* activity (Figure 3D), thereby reinforcing the likelihood that BfmR directly regulates *rhlR*. However, the detailed effect of BfmR on the *rhlR* gene expression awaits further study, since the *rhlR* promoter harbors multiple transcription start sites [15], regulatory sequences [15], and at least three BfmR-protected regions (Figure 3B). To our knowledge, this is the first evidence of a two-component regulator regulating *rhlR* in a direct manner. However, this finding is in contrast to a previous report suggesting that BfmR functions independently of QS signaling [24]. The exact cause of this discrepancy remains unknown. In the previous report [24], Petrova *et al* drew the conclusion based on the fact that deletion of *bfmR* has no significant effect on the transcript abundance of *rhlA* and *lasB*. Consistent with this, we observed that deletion of *bfmRS* has no significant effect on *rhl*-dependent phenotypes (Figure 1A and 1B) and the expression of *rhlA* (Table S4 in Text S1). However, deletion of *bfmS* alone leads to the activation of BfmR (Figure 1D and S4B), which in turn directly binds to the promoter and decreases the expression of the *rhlR* (Figure 3), causing the inhibition of the *rhl* QS system (Figure 1 and S3). Additionally, the decreased expression of *rhlR* in Δ*bfmS* strain (Figure 3C and 3D) may contribute to the attenuated virulence in lettuce leaves (Figure 5A) and the reduced production of QS signal C4-HSL, pyocyanin and rhamnolipids (Figure 1 and S1), since the constitutive expression of *rhlR* in Δ*bfmS* strain could restore these phenotypes to wild-type levels or higher (Figure 5B and S11). Moreover, the expression of *rhlI* in the Δ*bfmS* strain was significantly lower (>2-fold) than that of the wild-type strain (Tables S3 and S4 in Text S1). Thus, the *bfmS* deletion, which results in activation of BfmR, affects all aspects of the *rhl* QS system.

Deletion of *bfmS* impacts the expression of 202 genes that comprise 3.6% of the *P. aeruginosa* genome (Tables S2 and S3 in Text S1). These observations suggest that BfmS acts as a global regulator in *P. aeruginosa*. Besides regulating *rhl* quorum sensing, BfmS also regulates the expression of a large number of genes such as *PA4103*, *PA4104*, *PA4105*, *PA4106* and *PA4107*, whose transcripts are likely independent of the quorum-sensing regulated [48]. Thus, it is not surprising that BfmS has a profound effect on the expression of virulence-associated traits and the ability of *P. aeruginosa* to adapt to the host (Figure 5). Interestingly, BfmS has a positive impact on acute virulence phenotypes (Figure 5A and 5E), but a negative effect on biofilm formation (Figure S10) that acts as a major virulence-associated trait contributing to chronic infections [49]. This formation suggests that BfmS may play an important role in mediating the switch between the acute and chronic infection lifestyles of *P. aeruginosa*.

*P. aeruginosa* can cause serious acute and chronic infections in humans and it underwent numerous genetic adaptations during evolution in the CF airways, resulting in remodeling of the regulatory networks to match the fluctuations in the environment of CF lung [36], [50]–[52]. *bfmS* in *P. aeruginosa* CF isolates are often found to undergo missense mutations. For instance, L181P (point mutations causing the substitution of leucine by proline at codon 181) or L181P/E376Q in at least 10 DK2 strains [36], R393H in RP73 strain [38], A21P/T120K/L164F in either LESB58 or LES431strain, L164F in c7447m strain, A4T in CIG1 strain, T120K/L163V/L164F in C3719 strain, D295N in CF5 strain, and P6S/L164F in CF614 strain (http://www.ncbi.nlm.nih.gov/). We found that specific missense mutations in *bfmS* gene (L181P, L181P/E376Q, and R393H) result in elevated BfmR activity (Figure 6 and Figure S9), which contributes to biofilm formation (Figure S10) [24], [25] as well as to the inhibition of the *rhl* QS system (Figures 1, 3, and S3). It is well known that biofilm formation enables *P. aeruginosa* to cause persistent infections [49] while the loss of quorum sensing is one of the dominating changes that occur during the adaptive process of the *P. aeruginosa* in the CF lung [36], [50]. Thus, the naturally occurring missense mutations in BfmS may provide a selective advantage to either DK2 strains or RP73 strain during the course of chronic infection in CF lungs. However, it should be noted that the *P. aeruginosa* community in the CF lung is very dynamic [50], [51], [53] and only a fraction of the isolates will most probably possess these mutations. Therefore, the role of these missense mutations in the chronic lung infection awaits further investigation.

Intriguingly, although BfmS functions as a negative regulator of BfmR (Figure 1D and S4B), the naturally occurring missense mutations in *bfmS* gene (L181P, L181P/E376Q, and R393H) can produce BfmS variants that no longer repress, but instead activate BfmR (Figure 6 and Figure S9). These “reverse function” mutants of BfmS may exhibit an elevated ratio of kinase to phosphatase activity on BfmR, given that the activation of BfmR requires the phosphorylation on D55 (Figure S7). In agreement with this notion, we found that H238, the conserved histidine residues predicted to be involved in the autophosphorylation of BfmS, is required for BfmS_L181P/E376Q_ to activate BfmR (Figure 6D and 6E). The occurrence of BfmS “reverse function” mutants is not strain dependent, as evidenced by the fact that either DK2 lineage-specific mutations or RP73-specific mutation in *bfmS* could reverse its function against BfmR. The L181P mutation was located in the HAMP domain of BfmS, while the E376Q and the R393H mutation were located in ATP-binding domain. Currently, it is not clear how these missense mutations change the function of BfmS. Although further studies are needed to elucidate this elegant mechanism, our genetic analyses clearly indicated that naturally occurring missense mutations in *P. aeruginosa* gene could result in reverse of function, rather than simply loss (weakened) or gain (strengthened) of function.

Noticeably, *bfmRS* operon and BfmR-activated transcripts such as *PA4103*-*4104* and *PA4107*-*4106*-*4105* (Figure S2, Tables S2 and S4 in Text S1), were dramatically up-regulated in the lungs of cystic fibrosis patients compared to *in vitro* planktonic bacteria, indicating that BfmRS system is likely activated during chronic infection in CF lungs [23]. These genes also exhibit much higher gene expression levels in some *P. aeruginosa* CF isolates such as DK2-lineage strains (late stage infection isolates) [54] and E601 strain [55] compared to wild-type laboratory strain PAO1. These observations and results from the current study suggest that BfmRS TCS may sense and respond to environmental stress in CF lungs. We envision that further studies aimed at the characterization of the stimuli that BfmRS and/or its variants detect within the host could be of great importance to a full understanding of the mechanisms that make *P. aeruginosa* a successful pathogen and to the development of novel strategies to limit its infections.

## Materials and Methods

### Ethics statement

Animal experiments were performed in strict accordance with the Regulations for the Administration of Affairs Concerning Experimental Animals approved by the State Council of People's Republic of China (11-14-1988). All animal procedures were approved by the Institutional Animal Care and Use Committee (IACUC) of Shanghai Public Health Clinical Center (Permit Number: 2013P201). The laboratory animal usage license number is SYXK-HU-2010-0098, certificated by Shanghai Committee of Science and Technology.

### Bacterial strains, plasmids, and culture conditions

The bacterial strains and plasmids used in this study are listed in Table S1 in Text S1. Unless noted otherwise, *P. aeruginosa* MPAO1 [56] and its derivatives were grown in Luria-Bertani (LB) broth, Pyocyanin production broth [57] (PPB: 20 g peptone, 1.4 g MgCl_2_, 10 g K_2_SO_4_, 20 ml glycerol per liter; pH 7.0), or M8-glutamate minimal medium [27] (6 g Na_2_HPO_4_, 3 g KH_2_PO_4_, 0.5 g NaCl, 0.24 g MgSO_4_, 0.5 g glutamate per liter; pH 7.4) supplemented with 0.2% glucose, as indicated. *E. coli* cultures were grown in Luria-Bertani (LB) broth. All cultures were incubated at 37°C with shaking (250 rpm). For plasmid maintenance in *P. aeruginosa*, the medium was supplemented with 100 µg/ml carbenicillin or 100 µg/ml kanamycin when required. For plasmid maintenance in *E. coli*, the medium was supplemented with 100 µg/ml carbenicillin, 50 µg/ml kanamycin, 300 µg/ml trimethoprim, or 10 µg/ml gentamicin, as appropriate. For marker selection in *P. aeruginosa*, either 30 µg/ml gentamicin or 10 µg/ml tetracycline were used when required.

### Construction of *P. aeruginosa* Δ*bfmS*, Δ*bfmRS* and Δ*bfmS*Δ*ackA*-*pta* mutants

For gene replacement, a SacB-based strategy [58] was employed as described in our previous study [26]. To construct the *bfmS* null mutant (Δ*bfmS*), polymerase chain reactions (PCRs) were performed in order to amplify sequences upstream (1,574 bp) and downstream (1,562 bp) of the intended deletion. The upstream fragment was amplified from MPAO1 genomic DNA using primers BfmSupF (with *Eco*RI site) and BfmSupR (with *Xba*I site), while the downstream fragment was amplified with primers, BfmSdownF (with *Xba*I site) and BfmSdownR (with *Hin*dIII site). The two PCR products were digested and then cloned into the *Eco*RI/*Hin*dIII-digested gene replacement vector pEX18Ap, yielding pEX18Ap::*bfmS*UD. A 1.8 kb gentamicin resistance cassette was cut from pPS858 with *Xba*I and then cloned into pEX18Ap::*bfmS*UD, yielding pEX18Ap::*bfmS*UGD. The resultant plasmid, pEX18Ap::*bfmS*UGD, was electroporated into MPAO1 with selection for gentamicin resistance. Colonies were screened for gentamicin sensitivity and loss of sucrose (5%) sensitivity, which typically indicates a double-cross-over event and thus marks the occurrence of gene replacement. The Δ*bfmS* strain was further confirmed by PCR.

A similar strategy was used to construct the Δ*bfmRS* strain as described above. Briefly, the upstream fragment (1,832 bp) of the intended deletion was amplified with primers BfmRupF (with *Eco*RI site) and BfmRupR (with *Xba*I site). The downstream fragment (1,562 bp) was amplified with primers, BfmSdownF (with *Xba*I site) and BfmSdownR (with *Hin*dIII site). A 1.8 kb gentamicin resistance cassette was cut from pPS858 with *Xba*I and then cloned into pEX18Ap::*bfmRS*UD, yielding pEX18Ap::*bfmRS*UGD.

Again, a similar strategy was used to construct the Δ*bfmS*Δ*ackA*-*pta* strain. Primers Acka-up-F (with *Kpn*I site) and Acka-up-R (with *Bam*HI site) amplified the upstream fragment (2,245 bp) of the intended deletion of *ackA*-*pta* operon in Δ*bfmS*. Primers Pta-domn-F (with *Bam*HI site) and Pta-domn-R (with *Hin*dIII site) amplified the downstream fragment (1,709 bp). A 2.3 kb tetracycline resistance cassette was amplified from the integration vector mini-CTX-lacZ with primers, Mini-TC-F (with *Bam*HI site) and Mini-TC-F (with *Bam*HI site). The resultant plasmid, pEX18Ap::*acka-pta*UTD, was electroporated into Δ*bfmS* strain with selection for tetracycline resistance. Colonies were screened for tetracycline sensitivity and loss of sucrose (5%) sensitivity, which typically indicate a double-cross-over event and thus mark the occurrence of gene replacement. PCR further confirmed the deletion of *pta-acka* loci.

### Construction of chromosomal-borne BfmR-Flag strains

Primers bfmRflag-F (with *Hin*dIII site) and bfmRflag-R (with *Bam*HI site) (Table S6 in Text S1) were used to perform PCR of the BfmR gene that was meant to fuse with the Flag-tag. A 1,586-bp PCR product covering the region from 848 bp upstream and the BfmR gene (not including the stop codon) was generated. The *Hin*dIII- and *Bam*HI-digested PCR product was cloned into the *Hin*dIII and *Bam*HI sites of the mini-CTX-lacZ [59] to generate mini-ctx-BfmR-Flag. The resulting plasmid was conjugated into *P. aeruginosa* MPAO1 and Δ*bfmRS* strains and the construct was integrated into the *attB* site as described previously though a diparental mating using *E. coli* S17 λ-pir as the donor, yielding a MPAO1::*BfmR*-*Flag* strain and a Δ*bfmRS*::*BfmR*-*Flag* strain, respectively (Table S1 in Text S1). In these mutant strains, parts of the mini-CTX-*lacZ* vector containing the tetracycline resistance cassette were deleted using a flippase (FLP) recombinase encoded on the pFLP2 plasmid.

### Plasmid construction for the constitutive expression of *bfmR*, *bfmS*, *bfmRS*, *rhlR*, and *acka-pta* operon

To construct the plasmid for constitutive expression of *bfmR*, a 806 bp PCR product covering 15 bp of the *bfmR* upstream region, the *bfmR* gene, and 50 bp downstream of *bfmR* was amplified using primers BfmR(comp)Fwr (with *Hin*dIII site) and BfmR(comp)Rev (with *Bam*HI site). The product was digested with *Hin*dIII and *Bam*HI and ligated into PAK1900 [60] in the same orientation as p*lac* to generate p-*bfmR*.

To construct the plasmid for the constitutive expression of *bfmS*, a 1,385 bp PCR product covering 30 bp of the *bfmS* upstream region, the *bfmS* gene, and 50 bp downstream of *bfmS* was amplified using primers BfmS(comp)Fwr (with *Hin*dIII site) and BfmS(comp)Rev (with *Bam*HI site), and then cloned into PAK1900, yielding p-*bfmS*.

To construct the plasmid for the constitutive expression of *bfmRS*, a 2,107 bp PCR product covering 15 bp of the *bfmR* upstream region, the *bfmRS* operon, and 50 bp downstream of *bfmS* was amplified using primers BfmR(comp)Fwr (with *Hin*dIII site) and BfmS(comp)Rev (with *Bam*HI site) and then cloned into PAK1900, yielding p-*bfmRS*.

To construct the plasmid for the constitutive expression of *rhlR*, a 770 bp DNA fragment covering 44 bp of the *rhlR* upstream region and the *rhlR* was amplified using primers RhlR-OE-F (with *Hin*dIII site) and RhlR-OE-R (with *Hin*dIII site) and then cloned into PAK1900. The construct with *rhlR* in the same orientation as p*lac* was confirmed by DNA sequencing, yielding p-*rhlR*.

To construct the plasmid for constitutive expression of *acka-pta* operon, a 3,563 bp DNA fragment covering 136 bp of *acka* upstream region, the *acka-pta* operon, and a 65 bp downstream of *pta* was amplified using primers Acka-comp-F (with *Hin*dIII site) and Pta-comp-R (with *Bam*HI site) and then cloned into PAK1900, yielding plasmid p-*ackA-pta*.

The five mutations, p-*bfmR_D55A_*, p-*bfmS_L181P_*, p-*bfmS_E376Q_*, and p-*bfmS_L181P_*_/*E376Q*_, and p-*bfmS_R393H_* were obtained using the QuikChange II site-directed mutagenesis kit (Stratagene). For generating p-*bfmR_D55A_*, the primer pair BfmR(D55A)-F/BfmR(D55A)-F was used. For generating p-*bfmS_L181P_*, primer pair PA4102L181P-F/PA4102L181P-R was used. For generating p-*bfmS_E376Q_*, the primer pair PA4102E376Q-F/PA4102E376Q-R was used. For generating p-*bfmS_L181P_*_/*E376Q*_, primer pairs PA4102L181P-F/PA4102L181P-R and PA4102E376Q-F/PA4102E376Q-R were used. For generating p-*bfmS_R393H_*, the primer pairs R393H-F/R393H-R was used.

All constructs were sequenced to ensure that no unwanted mutations resulted.

### Construction, expression, and purification of 6His-BfmR, 6His-BfmR_D55A_, and BfmS_34–154_

Full-length of *bfmR* was cloned into pET28a with a thrombin-cleavable N-terminal His-tag. Primers bfmR-F (with *Nde*I site) and bfmR-R (*Xho*I) were used to amplify the *bfmR* gene from *P. aeruginosa* MPAO1 chromosomal DNA. The amplified fragments were ligated into similarly cut pET28a (Novagen) in order to produce the plasmids pET28a-6His-BfmR. pET28a-6His-BfmR_D55A_ was obtained by using the primer pair BfmR(D55A)-F/BfmR(D55A)-R and a QuikChange II site-directed mutagenesis kit (Stratagene). The protein was expressed in the *E. coli* strain BL21 star (DE3) and purifications were performed as described in our previous studies [26], [61], [62]. Briefly, bacteria were grown at 37°C overnight in 10 ml of LB medium (containing 50 µg/ml kanamycin) with shaking (250 rpm). The next day, the cultures were transferred into 1 L of LB medium (containing 50 µg/ml kanamycin) incubated at 37°C with shaking (250 rpm) until the OD600 reached 0.6, and then IPTG (isopropyl-1-thio-β-d-galactopyranoside) was added to a final concentration of 1.0 mM. After 4 h incubation at 30°C with shaking (250 rpm), the cells were harvested by centrifugation and stored at −80°C. The cells were lysed at 4°C by sonication in lysis buffer [10 mM Tris (pH 7.4), 300 mM NaCl, 1 mM PMSF, and 2 mM DTT]. Clarified cell lysate was loaded onto a HisTrap HP column (Amersham Biosciences), washed with Ni-NTA washing buffer, and eluted with Ni-NTA elution buffer. The fractions containing 6His-BfmR or 6His-BfmR_D55A_ were concentrated and loaded onto a Superdex-200 gel filtration column with a running condition of 10 mM Tris (pH 7.4), 300 mM NaCl, and 2 mM DTT. The purified protein was >90% pure as estimated by a 12% (wt/vol) SDS/PAGE gel.

The DNA sequence of the extracellular sensory domain of BfmS consisting of 121 residues (Gln34-Trp154) was amplified from MPAO1 genomic DNA with the primers PA4102-EX-F (with *Nco*I) and PA4102-EX-R (with *Bam*HI) by PCR and was subsequently cloned into pET28b using *Nco*I and *Bam*HI as the restriction enzymes. Following confirmation by DNA sequencing, the recombinant plasmid (pET28b-*bfmS_34–154_*) was transformed into *E. coli* strain BL21 star (DE3). The extracellular sensory domain of BfmS (designated BfmS_34–154_) was expressed and purified as described above with some modifications. Briefly, bacteria were grown at 37°C overnight in 10 ml of LB medium (containing 50 µg/ml kanamycin) with shaking (250 rpm). The next day, the cultures were transferred into 1 L of LB medium (containing 50 µg/ml kanamycin) incubated at 37°C with shaking (200 rpm) until the OD600 reached 0.6, and then IPTG was added to a final concentration of 1.0 mM. After 16 h incubation at 16°C with shaking (200 rpm), the cells were harvested by centrifugation and stored at −80°C. The cells were lysed at 4°C by sonication in lysis buffer [50 mM Tris-HCl, pH 8.0, 100 mM NaCl, 10% glycerol 1 mM PMSF, and 2 mM DTT]. Clarified cell lysate was loaded onto a HisTrap HP column (Amersham Biosciences), and eluted with Ni-NTA elution buffer (50 mM Tris-HCl, pH 8.0, 100 mM NaCl, 20 mM imidazole 10% glycerol, and 1 mM DTT,). The fractions containing BfmS_34–154_ were concentrated and loaded onto a Superdex-75 gel filtration column with a running condition of 20 mM Tris-HCl, pH 8.0, 100 mM NaCl, 10% glycerol, 1 mM DTT. The purified protein was >90% pure as estimated by a 12% (wt/vol) SDS/PAGE gel.

### Monitoring gene expression by *lux*-based reporters

The plasmid pMS402 [63] carrying a promoterless *luxCDABE* reporter gene cluster was used to construct promoter-*luxCDABE* reporter fusions of the *bfmR* as described previously [28], [64]. For *bfmR-lux*, the *bfmR* promoter region (−463 to +18 of the start codon) was amplified by PCR using the primers PMS402-bfmR-F (with *Xho*I site) and PMS402-bfmR-R (with *Bam*HI site). For *rhlA-lux*, the *rhlA* promoter region (−526 to −20 of the start codon) was amplified by PCR using the primers pms402-rhlA-F (with *Xho*I site) and pms402-rhlA-R (with *Bam*HI site). For *rhlR-lux*, the *rhlR* promoter region (−450 to +19 of the start codon) was amplified by PCR using the primers pms402-rhlR-1F (with *Bam*HI site) and pms402-rhlR-R (with *Bam*HI site). The promoter oriented in the same direction as *luxCDABE* was selected for further analysis. To generate *rhlR* promoter mutant *rhlR-D* (deletion of GATACT, which is the central part of the putative BfmR-binding site on the reverse DNA strand), the DNA fragment was amplified using primers pms402-rhlR-1F/pms402-rhlR-R and subsequently cloned into pGEM-T vector. *rhlR-D* (*rhlR* promoter lacking putative BfmR-binding site) was obtained using a QuikChange II site-directed mutagenesis kit (Stratagene) and primer pair pms402-rhlR(D1)F/pms402-rhlR(D1)R. For *4103-lux*, the *PA4103* promoter region (−659 to +19 of the start codon) was amplified by PCR using primers pms402-p4103-F (with *Xho*I site) and pms402-p4103-R (with *Bam*HI site). To generate *PA4103* promoter mutant *4103-M* (GATACA was mutated to ATATAT), primer pair p4103-mutation-F/p4103-mutation-R was used as described above. The promoter regions were cloned into the *Xho*I-*Bam*HI site or *Bam*HI site (for *rhlR-lux*) upstream of the *lux* genes on pMS402 and the cloned promoter sequences were confirmed by DNA sequencing. The constructs were transformed into MPAO1 or its derivatives by electroporation. Use of these *lux*-based reporters, gene expression under different conditions was measured as counts per second (cps) of light production with a 2104 EnVision Multilabel Plate Readers or Synergy 2 (Biotek). Relative light units were calculated by normalizing CPS to OD_600_.

### Quantitative real-time PCR

The bacterial growth and the extraction of total RNAs were performed as described above. The total DNase-treated RNA (5 µg) was reversely transcribed to synthesize cDNA using the PrimeScript RT reagent Kit (Takara) with random primers according to the manufacturer's protocol. The resulting cDNA were diluted by 1∶2, 1∶4, and 1∶8 respectively. Triplicate quantitative assays were performed on 1 µl of each cDNA dilution with the THUNDERBIRD SYBR qPCR Mix and 300 nM primers using an Applied Biosystems 7500 Fast Real-Time PCR System. Dissociation curve analysis was performed in order to verify product homogeneity. The gene-specific primers used for Quantitative real-time PCR for *PA4100*, *PA4103*, *PA4107*, *PA4108*, *ntrB*, *oprH*, *phoB*, *hmgA*, *rhlA*, *antA*, *nasA*, and *rhlI* are listed in Table S6 in Text S1. The amplicon of 16S rRNA was used as an internal control in order to normalize all data. Relative expression levels of interest genes were calculated by the relative quantification method (ΔΔCT) as previously described [65], [66].

### Sample preparation for *in vivo* detection of BfmR phosphorylation and Phos-tag gel electrophoresis

*P. aeruginosa* was grown at 37°C for 24 h on M8-glutamate minimal agar plate (M8-glutamate minimal medium supplemented with 0.2% glucose, and solidified with 2% agar). To prepare cell lysates for the Phos-tag gel assay, bacteria cells were scraped from the plate and immediately resuspended in 60 µl of lysis buffer [50 mM Tris-Cl (pH 7.5), 150 mM NaCl, 1 mM MgCl_2_, 0.1% Triton X-100, 15 µg/ml DNaseI, 0.5 mM PMSF, 1 mM DTT) with 0.1% (vol/vol) Lysonase. Sufficient lysis was achieved by repeated pipetting up and down for 10 s followed by addition of 20 µl of 4×SDS loading buffer. Resulting cell lysates (10 µl) were immediately loaded onto a Phos-tag gel for electrophoresis.

BfmR-flag and BfmR-flag∼P were separated on 10% acrylamide gels containing 25 µM acrylamide-Phos-tag ligand (Wako Pure Chemical) and 50 µM MnCl_2_ as previously described [67]. Electrophoresis was performed at 30 mA at 4°C for 80 min in Tris-Glycine-SDS running buffer (25 mM Tris, 192 mM glycine, 0.1% SDS, pH 8.4). After electrophoresis, the Phos-tag gel was washed 10 min at RT with Transfer Buffer [20%(v/v) methanol, 50 mM Tris, 40 mM glycine] supplied with 1 mM EDTA to remove Zn^2+^ from the gel, then the gel was incubated at room temperature with gentle shaking for another 10 min in Transfer Buffer twice to remove EDTA.

### Western blot analysis

Samples resolved on gels were transferred to PVDF (Bio-Rad) membranes through semi-dry transfer assembly (Bio-Rad) for 30 min at room temperature. BfmR-Flag proteins were detected by Western blot analysis using a mouse anti-Flag monoclonal antibody (Cat^#^: AGM12165, Aogma) followed by a secondary, sheep anti-mouse IgG antibody conjugated to horseradish peroxidase (HRP) (Code^#^: NA931, GE Healthcare). For detection of ClpP protein, anti-ClpP polyclonal antibody and anti-rabbit IgG antibody conjugated to horseradish peroxidase (HRP) (Code^#^: NA934, GE Healthcare) were used. Anti-ClpP polyclonal antibody, which cross-reacts with the ClpP of *Pseudomonas aeruginosa*, was prepared by immunizing a rabbit with a *Staphylococcus aureus* full-length ClpP protein (NWMN_0736). For detection of BfmS protein, anti-BfmS polyclonal antibody (prepared by immunizing a rabbit with a BfmS_34–154_ protein, Shanghai Immune Biotech CO., Ltd) and anti-rabbit IgG antibody conjugated to horseradish peroxidase (HRP) (Code^#^: NA934, GE Healthcare) were used. For detection of RNAP protein, anti-RNAP (Neoclone, #WP003) antibody and anti-mouse IgG antibody conjugated to horseradish peroxidase (HRP) (Code^#^: NA931, GE Healthcare). Immunoblots for ClpP and RNAP served as loading control. The membrane is exposed to X-ray film (Kodak) or the chemiluminescent is detected by a Imaging Quant LAS-4000 (GE), according to the manufacturer's recommendation.

### Measurement of pyocyanin production

Pyocyanin was extracted from culture supernatants and measured using previously reported methods [68]. Briefly, *P. aeruginosa* was grown in Pyocyanin production broth [57] (PPB: 20 g peptone, 1.4 g MgCl_2_, 10 g K_2_SO_4_, 20 ml glycerol per liter; pH 7.0) for 36 h at 37°C with shaking (250 rpm). The culture was subsequently centrifuged and filtered (pore size, 0.22 µm). 1.5 ml of chloroform was added to 2.5 ml of culture supernatant. After extraction, the chloroform layer was transferred to a fresh tube and mixed with 1 ml of 0.2 N HCl. After centrifugation, the top layer (0.2 N HCl) was removed and its absorption measured at 520 nm. Concentrations, expressed as micrograms of pyocyanin produced per ml of culture supernatant, were determined by multiplying the optical density at 520 nm (OD520) by 17.072.

### Measurement of rhamnolipid production

Rhamnolipids production was estimated by inoculating strains on M8-based agar plates supplemented with 0.2% glucose(m/v), 2 mM MgSO_4_, 0.0005% (m/v) methylene blue, and 0.02% (m/v) cetyltrimethylammonium bromide, as described previously [68], [69]. The orcinol assay was used to directly assess the amount of rhamnolipids in the sample as previously described [28]. After a culture of 48 h in LB medium at 37°C with shaking (250 rpm), 1 ml of the culture supernatant was extracted twice with 2 ml of diethyl ether. The pooled ether fractions were evaporated to dryness and the remainder was dissolved in 100 µl of distilled water and mixed with 100 µl of 1.6% orcinol, and 800 µl of 60% sulfuric acid. After heating for 30 min at 80°C in the dark, the samples were cooled for 3 h at room temperature in the dark. Absorbance at 421 nm (A421) was measured. Rhamnolipid concentrations were calculated by comparing A421 values with those obtained for rhamnose standards between 0 and 1000 µg/ml, assuming that 1 µg of rhamnose corresponds to 2.5 µg of rhamnolipids.

### Swarming motility assays

The motility assay was carried out as described previously [27], [68]. Swarming medium was based on M8-glutamate minimal medium [27] (6 g Na_2_HPO_4_, 3 g KH_2_PO_4_, 0.5 g NaCl, 0.24 g MgSO_4_, 0.5 g glutamate per liter; pH 7.4), supplemented with MgSO_4_ (2 mM), glucose (0.2%), and Casamino acid (0.5%), and solidified with 0.5% agar. Bacteria were inoculated with a toothpick onto swarm agar plates. Swarm agar plates were incubated for 24 hours at 37°C and then incubated for more time at room temperature.

### *In vitro* phosphorylation assays

Phosphorylation of 6His-BfmR was detected by utilizing the Pro-Q Diamond phosphorylation gel stain as described by the manufacturer (Invitrogen). Purified 6His-BfmR and 6His-BfmR_D55A_ were incubated with buffer (10 mM Tris pH 8.0; 1 mM DTT; 5 mM MgCl_2_; 10 mM KCl; 50 mM acetyl phosphate) at 37°C for 30 min. The acetyl phosphate-treated samples of 6His-BfmR and 6His-BfmR_D55A_ were resolved on a 12% SDS polyacrylamide gel, and then the gel was immersed in fixing solution (10% acetic acid, 50% methanol) for 30 min and subsequently washed three times with deionized water each for 10 min. The gel was stained with Pro-Q Diamond phosphoprotein gel stain for 60 min, followed by washing with deionized water for 30 min. The entire procedure was conducted at room temperature. Fluorescent output was recorded using an Tanon-5200 multi.

### Electrophoretic mobility shift assay (EMSA)

The electrophoretic mobility shift experiments were performed as described in our previous studies with some modifications [26], [61], [62]. Briefly, 20 µl of the DNA probe mixture (30 to 50 ng) and various amounts of purified proteins in binding buffer (10 mM Tris-Cl, pH 8.0; 1 mM DTT; 10% glycerol; 5 mM MgCl_2_; 10 mM KCl) were incubated for 30 min at 37°C. When indicated, 50 mM acetyl phosphate was added to the solution. Native polyacrylamide gel (6%) was run in 0.5× TBE buffer at 85 V at 4°C. The gel was stained with GelRed nucleic acid staining solution (Biotium) for 10 min, and then the DNA bands were visualized by gel exposure to 260-nm UV light.

DNA probes were PCR-amplified from *P. aeruginosa* MPAO1 genomic DNA using the primers listed in Table S6 in Text S1. The probes for *bfmR* promoter, a 481 bp DNA fragment covering the promoter region of *bfmR* (from −463 to +18 of the start codon) was amplified using primers bfmR-F(EMSA) and bfmR-R(EMSA). For *rhlR* promoter, a 470 bp DNA fragment covering the promoter region of *rhlR* (from −450 to +20 of the start codon) was amplified using primers rhlR-F(EMSA) and rhlR-F(EMSA). For *rhlI* promoter, a 446 bp DNA fragment covering the promoter region of *rhlI* (from −444 to +2 of the start codon) was amplified using primers rhlI-F(EMSA) and rhlI-R(EMSA). For *rhlA* promoter, a 572 bp DNA fragment covering the promoter region of *rhlA* (from −591 to −19 of the start codon) was amplified using primers rhlA-F(EMSA) and rhlA-R(EMSA). For *rhlC* promoter, a 540 bp DNA fragment covering the promoter region of *rhlC* (from −549 to −9 of the start codon) was amplified using primers rhlC-F(EMSA) and rhlC-F(EMSA). For *PA4103* promoter, a ca. 0.7 kb DNA fragment (*4103*-P) containing the promoter region of *PA4103* (from −659 to +19 of the start codon) was amplified from plasmid *4103-lux* DNA using primers pZE.05 and pZE.06. For *PA4107* promoter, a 360 bp DNA fragment (*4107*-P) covering the promoter region of *PA4107* (from −490 to −131 of the start codon) was amplified from *P. aeruginosa* MPAO1 genomic DNA using primers PA4108-F and PA4108-R. All PCR products were purified by using a QIAquick gel purification kit (QIAGEN).

### Dye primer-based DNase I footprinting assay

The published DNase I footprint protocol was modified [70] in this study in the same way as described in our previous study [61]. Briefly, PCR was used to generate DNA fragments using the primer sets as detailed in Table S6 in Text S1. For amplification of *bfmR* promoter, primers bfmR-F(EMSA) and 6FAM-bfmR-R were used. For amplification of the *rhlR* promoter, primers rhlR-F(EMSA) and 6FAM-rhlR-R were used. For amplification of the *PA4103* promoter, p4103-F (EMSA) and p4103-R-FAM were used. All PCR products were purified by with QIAquick gel purification kit (QIAGEN). 50 nM 6-carboxyfluorescein (6-FAM)-labeled promoter DNA and various amounts of 6His-BfmR (as indicated) in 50 µl of binding buffer (10 mM Tris-Cl, pH 8.0; 1 mM DTT; 10% glycerol; 5 mM MgCl_2_; 10 mM KCl; 50 mM acetyl phosphate) were incubated at room temperature for 10 min. 0.01 unit of DNase I was added to the reaction mixture and incubated for 5 more min. The digestion was terminated by adding 90 µl of quenching solution (200 mM NaCl, 30 mM EDTA, 1% SDS), and then the mixture was extracted with 200 µl of phenol-chloroform-isoamyl alcohol (25∶24∶1). The digested DNA fragments were isolated by ethanol precipitation. 5.0 µl of digested DNA was mixed with 4.9 µl of HiDi formamide and 0.1 µl of GeneScan-500 LIZ size standards (Applied Biosystems). A 3730XL DNA analyzer detected the sample, and the result was analyzed with GeneMapper software.

### Analyses of gene expressions with oligonucleotide microarray

Overnight *P. aeruginosa* cultures were washed and diluted 100-fold in M8-glutamate minimal medium (6 g Na_2_HPO_4_, 3 g KH_2_PO_4_, 0.5 g NaCl, 0.24 g MgSO_4_, 0.5 g Glutamate per liter; pH 7.4) supplemented with glucose (2 g/L). The bacteria were subsequently grown at 37°C for 48 h (OD_600_≈1.0) with shaking (250 rpm). Total RNA was immediately stabilized with RNAprotect Bacteria Reagent (Qiagen, Valencia, CA) and then extracted using a Qiagen RNeasy kit following the manufacturer's instructions. The total DNase-treated RNA samples were then analyzed by CapitalBio Corp for Chip (Affymetrix) assay. Briefly, samples were labeled according to the manufacturer (Affymetrix, Santa Clara, CA) and then hybridized to the Affymetrix GeneChip *P. aeruginosa* genome array (catalog number AFF-900339) for 16 h at 50°C though the use of the GeneChip hybridization oven at 60 rpm. Washing, staining, and scanning were performed using the Affymetrix GeneChip system. The data were normalized using Robust Multi-array Average (RMA) [71]. Gene expression analysis was performed using three independent mRNA samples for each strain. Microarray data were analyzed using SAM (Significance Analysis of Microarrays) software [72]. Criterion such as cutoff limitation for fold change ≥2 or ≤0.5 and q-value ≤5% was used to select differential expression genes. All data were submitted to the ArrayExpress database (http://www.ebi.ac.uk/arrayexpress) under accession number E-MTAB-1983.

### Bioassay of C4-HSL activity

The autoinducer of the *rhl* system, C4-HSL, was measured using an *rhlA* promoter-based *P. aeruginosa* strain, pDO100 (pKD-*rhlA*) [28]. This detection system was developed by fusing the C4-HSL-responsive *rhlA* promoter upstream of *luxCDABE* and introducing the construct into pDO100, a *rhlI* mutant strain [28]. Procedures were modified from the protocol described previously [28]. Briefly, the reporter strain pDO100 (pKD-*rhlA*) was grown in LB medium plus 100 µg/ml kanamycin overnight at 37°C with shaking (250 rpm) and diluted to an OD_600_ of 0.05 in fresh LB plus kanamycin. 90 µl was subsequently added to the wells of a 96-well microtitre plate. A 10 ml portion of the samples or medium control was added to the wells. The luminescence value was measured in a 2104 EnVision Multilabel Plate Readers or Synergy 2 (Biotek), and calculated from the luminescence value minus that of the medium control. The data are presented as CPS and are not normalized to OD_600_ of pDO100 (pKD-*rhlA*). In this assay, the growth curves of pDO100 (pKD-*rhlA*) are identical.

### Infection of MLE-12 cells

Different strains of bacteria were grown overnight in Luria-Bertani (LB) broth at 37°C with shaking. Then, the bacteria were subject to pelleting by centrifugation at 5,000 g and resuspended in 10 ml of fresh LB broth and allowed to grow until the mid-logarithmic phase. OD600 nm was measured, and the density was adjusted to 0.25 OD (0.1 OD = 1×10^8^ cells/ml). Mammalian cells were washed once with PBS after overnight culture in full medium, and changed to a serum-free and antibiotic-free medium immediately before infection. Cells were infected by various strains at a multiplicity of infection (m.o.i) of 10∶1 bacteria-to-cell ratio at indicated time points for 2 h. The cells were washed three times with PBS to remove surface bacteria and incubated with 100 µg/ml polymyxin B for 1 h. The cells were lyzed to evaluate the internalized bacteria using CFU assay on agar dishes as described in our previous studies [73], [74].

### 3-(4,5-dimethylthiazol-2-yl)-2,5-dimethyltetrazolium Bromide (MTT) assay

The killing of MLE-12 cells by bacterial infection was performed by continuing incubation for 24 h and cell survival measured using MTT assay [75]. MLE-12 cells were cultured in 96-well plates as above. After incubation for 24 h, MTT dye was added to the cells in each well with at a final concentration of 1 µg/ml. Then, the cells were incubated at 37°C until the color developed. The yellow color may change to brown upon reduction by enzymes. The reaction was terminated by adding 100 µl of stop solution (10% DMSO, 10% SDS in 50 mM HEPES buffer). The plate was left at room temperature overnight. The next day, the 560-nm absorbance was read using a plate reader in order to quantify the dye conversion [76]. Background correction was done with controls containing only the dye.

### Lettuce leaf model of infection

A lettuce leaf virulence assay was performed as described previously [77]–[79]. Briefly, *P. aeruginosa* strains were grown aerobically overnight at 37°C with shaking (250 rpm) in PPB broth or PPB broth containing carbenicillin (100 µg/ml) when appropriate, washed, resuspended, and diluted in sterile MgSO_4_ to a bacterial density of 1×10^9^ CFU/ml. Lettuce leaves were prepared by washing with sterile distilled H_2_O and 0.1% bleach. Samples (10 µl) were then inoculated into the midribs of Romaine lettuce leaves. Containers containing Whatman paper moistened with 10 mM MgSO4 and inoculated leaves were kept in a growth chamber at 37°C for five days. Symptoms were monitored daily. As a control, lettuce leaves were inoculated with 10 mM MgSO4.

### Mouse model of acute pneumonia

All *P. aeruginosa* strains were grown at 37°C overnight in PPB medium with shaking (250 rpm), diluted 100-fold in fresh PPB medium, and incubated at 37°C for 2.5–3.0 h until the cultures reached OD_600_ 0.8. Bacteria were collected by centrifugation, washed, and suspended in PBS buffer. Viable *P. aeruginosa* were enumerated by colony formation on *Pseudomonas* isolation agar (PIA) (Difco) plates in order to quantify the infectious dose. Mouse infections were carried out as described previously [26], using 8-week-old female C57BL/6J mice obtained from Shanghai SLAC Laboratory Animal Co. Ltd. and housed under specified pathogen-free conditions. Mice were anaesthetized with pentobarbital sodium (intraperitoneal injection, 80 mg/kg) and intranasally infected with c. 5×10^6^ cfu of each bacterial isolate. After that, animals were sacrificed 18 h post infection. Lungs were aseptically removed and homogenized in PBS plus 0.1% Triton X-100 in order to obtain single-cell suspensions. Serial dilutions of each organ were plated on *Pseudomonas* isolation agar (PIA) (Difco) plates. Bacterial burden per organ was calculated and is expressed as a ratio of the inoculum delivered per animal. Statistical analysis was performed using Prism software (GraphPad).

### Statistical analysis

Two-tailed Student's t tests or Mann–Whitney test was used to calculate p-values (two-tailed) using Prism software (GraphPad), as indicated. * p<0.05, ** p<0.01, *** p<0.001.

## Supporting Information

Figure S1 **Effect of** ***bfmS*** **deletion on the virulence-associated traits and the expression of** ***rhlA*** **in** ***P. aeruginosa*** **.** In all panels, MPAO1 is the wild-type MPAO1 strain harboring plasmid PAK1900, whereas Δ*bfmS* is the *bfmS* deletion mutant harboring plasmid PAK1900. **A**) Pyocyanin production. *P. aeruginosa* MPAO1 and its derivatives were grown in PPB medium at 37°C with shaking (250 rpm). After 36 h, pyocyanin was extracted with chloroform and its concentration was determined spectrophotometrically. **B**) Rhamnolipids production. The amounts of rhamnolipids in culture supernatants were determined by an indirect assay (orcinol test). Bacteria were grown in M8-glutamate minimal medium supplemented with 0.2% glucose at 37°C for 60 h with shaking (250 rpm). **C**) Representative swarm phenotypes of MPAO1 and its derivatives. The indicated bacterial strains were inoculated with a toothpick onto 0.5% swarm agar plates and incubated at 37°C for 24 h and then at room temperature for 48 h. **D**) and **E**) Expression of *rhlA-lux* and *rhlI-lux* (in pKD-*rhlI*, Table S1 in Text S1) in MPAO1 and its derivatives. Bacteria were grown in M8-glutamate minimal medium supplemented with 0.2% glucose at 37°C with shaking (250 rpm) and the *bfmR-lux* activity was measured, as indicated. The differences between groups were examined by two-tailed Student's t tests. ***, p<0.001. All the assays were independently repeated at least three times and the data shown represent comparable results. Values represent means ± standard error of the mean (SEM).
(TIF)

Figure S2 **Schematic presentation of genes whose expression is dramatically (>10-fold) affected by the deletion of** ***bfmS*** **.** The fold change indicates gene expression levels in Δ*bfmS* strain compared to expression in the wild-type MPAO1 strain. Genes with more than a 10-fold up- and down-regulation are highlighted in red and green, respectively. **A**) Genes located at or near the *bfmR*-*bfmS* locus of unknown function. **B**) *rhlAB* rhamnolipid biosynthetic operon. **C**) *antABC* anthranilate degradation operon. **D**) Genes/operons of nitrate assimilation. **E**) Genes/operons of unknown function.
(TIF)

Figure S3 **Effect of** ***bfmS*** **deletion on the promoter activities of** ***rhlR*** **,** ***rhlI*** **, and** ***rhlA*** **, and the C4-HSL contents.** In panels of **C**), **D**), and **E**), MPAO1 and Δ*bfmS* harbor plasmid PAK1900, respectively. **A**) Expression of *rhlR-lux* in MPAO1 and Δ*bfmS* mutant when bacteria were grown in M8-glutamate minimal medium (57.6 mM Pi) supplemented with 0.2% glucose at 37°C for 48 h with shaking (250 rpm). **B**) Expression of *rhlR-lux* in MPAO1 and Δ*bfmS* mutant when bacteria were grown low-phosphate (0.32 mM Pi) M8-glutamate minimal medium supplemented with 0.2% glucose at 37°C for 48 h with shaking (250 rpm). **C**) and **D**) Expression of *rhlI-lux* in MPAO1 and its derivatives when bacteria were grown low-phosphate (0.32 mM Pi) M8-glutamate minimal medium supplemented with 0.2% glucose at 37°C with shaking (250 rpm), as indicated. **E**) Relative amount of C4-HSL measured by the pDO100 (pKD-*rhlA*) system. Bacteria were grown low-phosphate (0.32 mM Pi) M8-glutamate minimal medium supplemented with 0.2% glucose at 37°C for 48 h with shaking (250 rpm). All experiments were independently repeated at least three times and the data shown represent comparable results. Values represent means ± standard error of the mean (SEM).
(TIF)

Figure S4 ***bfmR*** **is activated in the absence of** ***bfmS*** **.** In all panels, MPAO1::*BfmR*-*Flag* and Δ*bfmRS*::*BfmR*-*Flag* strain harbor the plasmid PAK1900. **A**) The absence of *bfmS* results in a greatly decreased pyocyanin production. Bacteria were grown for 24 h in PPB medium at 37°C with shaking (250 rpm). The presence of the blue-green pigment indicates pyocyanin production. **B**) Western blot analysis showing that the BfmR is significantly elevated in the absence of *bfmS*. ClpP protein is used as an internal control for loading error as described in the Materials and Methods section. Strains were grown in PPB medium with the appropriate antibiotic at 37°C for 24 h with shaking (250 rpm). The assays were repeated at least three times with similar results obtained.
(TIF)

Figure S5 **BfmR regulates the expression of** ***PA4107 and PA4103*** **in a direct manner.** **A**) and **B**) EMSA shows that 6His-BfmR directly binds to the promoter DNA of *PA4107* and PA4103 but not to that of *rhlI*, as indicated. **C**) Electropherograms show the protection pattern of the *PA4103* promoter DNA after digestion with DNase I following incubation in the absence or the presence of 6His-BfmR. BfmR-protected regions I harbors a putative BfmR-binding motif, which was highlighted in bold and in italics. **D**) The expression of *4103-lux* and *4103-M-lux* in wild-type MPAO1 or in the Δ*bfmS* strain, as indicated. Bacteria were grown in M8-glutamate minimal medium supplemented with 2% glucose at 37°C for 24 h. Values represent means ± SEM. The assays were independently repeated at least three times and the data shown represent comparable results.
(TIF)

Figure S6 **BfmR can be phosphorylated and activated by acetyl phosphate** ***in vitro*** **.** **A**) *In vitro* phosphorylation assays showing that 6His-BfmR but not 6His-BfmR_D55A_ could be phosphorylated by acetyl phosphate. Phosphorylated protein was detected by the Pro-Q Diamond phosphoprotein gel stain technique (upper panel), while total protein was visualized with the Coomassie brilliant blue stain (lower panel). Wild type (6His-BfmR) and the variant with the amino acid substitution of aspartate for alanine at amino acid 55 (6His-BfmR_D55A_) were purified by metal affinity chromatography. 2 µM of purified proteins was incubated in the absence (−) or presence (+) of 50 mM acetyl phosphate. Samples were separated in a conventional SDS 12% polyacrylamide gel. **B**) EMSA showing that acetyl phosphate enhances the DNA-binding ability of 6His-BfmR. The dissociation constants of 6His-BfmR to the *bfmR* promoter DNA: Kd = ∼0.2 µM, in the presence of acetyl phosphate (50 mM); Kd>0.6 µM, in the absence of acetyl phosphate. All experiments were repeated at least three times with similar results obtained.
(TIF)

Figure S7 **Different experiments indicate that aspartate residue D55 is required in order to activate BfmR.** **A**) EMSA showing that mutation of aspartate residue 55 to alanine attenuates the binding abilities of BfmR to its own promoter. Dissociation constants, Kd (6His-BfmR) ≤0.4 µM, Kd (6His-BfmR_D55A_) >0.6 µM, were determined by a densitometry analysis. **B**) Promoter reporter assays showing that mutation of aspartate residue 55 to alanine abolishes the ability of BfmR to induce *bfmR-lux* activity in Δ*bfmRS* strain. Bacteria were grown in M8-glutamate minimal medium supplemented with 0.2% glucose at 37°C for 24 h with shaking (250 rpm). Values are relative to MPAO1 (set to 1). Values represent means ± SEM. **C**) Phenotypic analysis showing that substitution of aspartate for alanine at amino acid 55 abolishes the ability of BfmR to repress the green pigment production of the Δ*bfmRS* strain. Bacteria were grown in PPB medium at 37°C for 24 h with shaking (250 rpm). All experiments were repeated at least twice with similar results obtained. In **B**) and **C**), MPAO1 and Δ*bfmRS* harbor plasmid PAK1900, respectively.
(TIF)

Figure S8 **BfmR regulates bacterial virulence.** In all panels, MPAO1 and Δ*bfmRS* strains harbor plasmid PAK1900. Photographs show lettuce midribs after three days of infection with 1×10^7^ cfu of *P. aeruginosa*. Δ*bfmRS* strain exhibits a virulence phenotype similar to that of a wild-type MPAO1 strain, while the introduction of p-*bfmR* into the Δ*bfmRS* strain leads to a low virulence phenotype. The assays were independently repeated at least three times and the data shown represent comparable results.
(TIF)

Figure S9 **Effects of amino acid substitutions in BfmS on the activation of BfmR.** **A**) Expressing *bfmS* and its derivatives on the *bfmR-lux* activity in Δ*bfmRS* strain. Δ*bfmRS* strain harbors plasmid PAK1900. Bacteria were grown in M8-glutamate minimal medium supplemented with 0.082% sodium acetate at 37°C for 36 h with shaking (250 rpm). Values are relative to Δ*bfmRS* strain harboring PAK1900 (set to 1). **B**) The relative *bfmR-lux* activity in Δ*bfmS*/p-*bfmS* strain (*bfmS*) and Δ*bfmS*/p-*bfmS_R393H_* strain (*bfmS_R393H_*) when bacteria were grown in M8-glutamate minimal medium supplemented with 0.2% glucose at 37°C for 36 h with shaking (250 rpm). Values are relative to Δ*bfmS*/p-*bfmS* strain (set to 1). **C**) The relative *bfmR-lux* activity in Δ*bfmS*/p-*bfmS* strain (*bfmS*) and Δ*bfmS*/p-*bfmS_R393H_* strain (*bfmS_R393H_*) when bacteria were grown in M8-glutamate minimal medium supplemented with 0.082% sodium acetate at 37°C for 36 h with shaking (250 rpm). Values are relative to Δ*bfmS*/p-*bfmS* strain (set to 1). **D**) Relative amount of C4-HSL measured by the pDO100 (pKD-*rhlA*) system. Δ*bfmS*/p-*bfmS* strain (*bfmS*) and Δ*bfmS*/p-*bfmS_R393H_* strain (*bfmS_R393H_*) were grown in M8-glutamate minimal medium supplemented with 0.2% glucose at 37°C for 36 h with shaking (250 rpm). Supernatants were subsequently prepared and measured for relative C4-HSL contents. The assays were independently repeated at least three times, and the data shown are representative of comparable results. Values represent means ± SEM. CPS, counts per second.
(TIF)

Figure S10 **Effect of** ***bfmRS*** **on the biofilm formation of** ***P. aeruginosa*** **.** In all panels, MPAO1, Δ*bfmS*, Δ*bfmRS*, MPAO1::*BfmR*-*Flag* and the Δ*bfmRS*::*BfmR*-*Flag* strain harbor plasmid PAK1900, respectively. MPAO1 and its derivatives were grown for five days in PPB medium at 37°C with shaking (250 rpm). Deletion of *bfmS* **A**), expressing either *bfmR* **B**) or *bfmR-Flag* **C**) in Δ*bfmRS* strain results in a greatly induced ring of biofilm at the air-liquid interface in shaken liquid cultures, as indicated by the arrow. The assays were independently repeated at least three times, and the data shown are representative of comparable results.
(TIF)

Figure S11 **Effect of expressing** ***rhlR*** **in Δ** ***bfmS*** **strain on the production of QS signal C4-HSL, rhamnolipid and pyocyanin.** **A**) Relative amount of C4-HSL measured by the pDO100 (pKD-*rhlA*) system. MPAO1 and its derivatives were grown in M8-glutamate minimal medium supplemented with 0.2% glucose at 37°C for 48 h with shaking (250 rpm). Supernatants were subsequently prepared and measured for the relative C4-HSL contents. **B**) Bacterial strains were inoculated onto a cetyltrimethylammonium bromide (CTAB) plate and incubated at 37°C for 24 h and then for 72 h at room temperature; the presence of a blue halo surrounding the colonies indicates production of rhamnolipids. **C**) MPAO1 and its derivatives were grown in Pyocyanin production broth (PPB) medium at 37°C for 24 h with shaking (250 rpm); the presence of the blue-green pigment indicates pyocyanin production. The assays were independently repeated at least three times, and the data shown are representative of comparable results. Values represent means ± SEM. CPS, counts per second.
(TIF)

Text S1 **Text S1 contains six Supplemental Tables (Table S1–6).** Table S1. Plasmids and bacterial strains used in this study; Table S2. 131 genes whose expressions are up-regulated more than 2-fold in Δ*bfmS* strain compared to wild-type MPAO1 strain; Table S3. 71 genes whose expression are down-regulated more than 2-fold in Δ*bfmS* strain compared to wild-type MPAO1 strain; Table S4. Verification of microarray results by Real-Time RT-PCR; Table S5. 41 promoters identified by consensus sequence search; Table S6. Primers used in this study.
(DOCX)
