# Supplementary figures and images for: A Novel Signal Transduction Pathway that Modulates rhl Quorum Sensing and Bacterial Virulence in Pseudomonas aeruginosa
Source: PLoS Pathog. 2014 Aug 28;10(8):e1004340. doi: 10.1371/journal.ppat.1004340 (PMC4148453; doi:10.1371/journal.ppat.1004340)

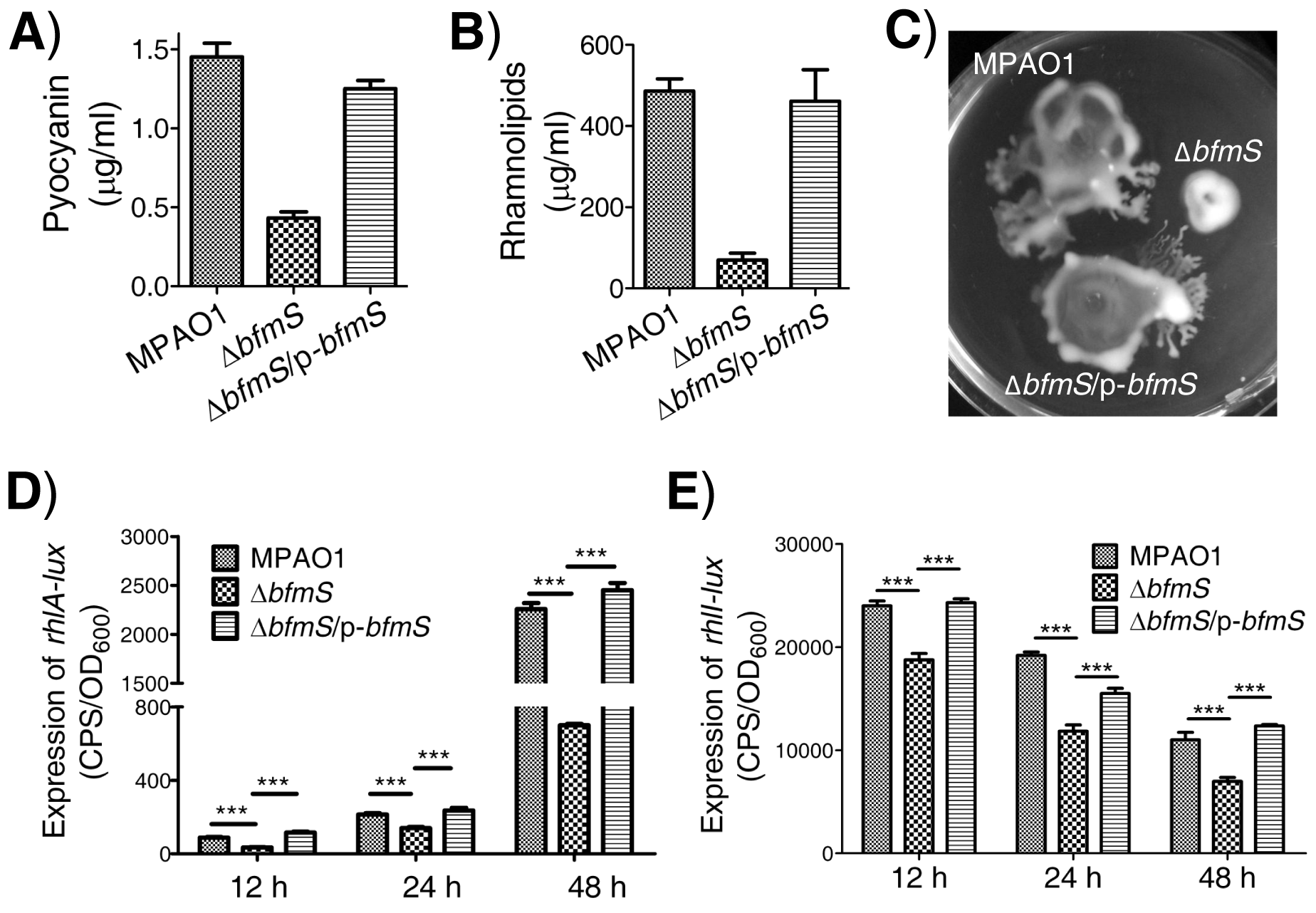

Supplement: Figure S1 — Effect of bfmS deletion on the virulence-associated traits and the expression of rhlA in P. aeruginosa . In all panels, MPAO1 is the wild-type MPAO1 strain harboring plasmid PAK1900, whereas ΔbfmS is the bfmS deletion mutant harboring plasmid PAK1900. A) Pyocyanin production. P. aeruginosa MPAO1 and its derivatives were grown in PPB medium at 37°C with shaking (250 rpm). After 36 h, pyocyanin was extracted with chloroform and its concentration was determined spectrophotometrically. B) Rhamnolipids production. The amounts of rhamnolipids in culture supernatants were determined by an indirect assay (orcinol test). Bacteria were grown in M8-glutamate minimal medium supplemented with 0.2% glucose at 37°C for 60 h with shaking (250 rpm). C) Representative swarm phenotypes of MPAO1 and its derivatives. The indicated bacterial strains were inoculated with a toothpick onto 0.5% swarm agar plates and incubated at 37°C for 24 h and then at room temperature for 48 h. D) and E) Expression of rhlA-lux and rhlI-lux (in pKD-rhlI, Table S1 in Text S1) in MPAO1 and its derivatives. Bacteria were grown in M8-glutamate minimal medium supplemented with 0.2% glucose at 37°C with shaking (250 rpm) and the bfmR-lux activity was measured, as indicated. The differences between groups were examined by two-tailed Student's t tests. ***, p<0.001. All the assays were independently repeated at least three times and the data shown represent comparable results. Values represent means ± standard error of the mean (SEM). (TIF) [file ppat.1004340.s001.tif]

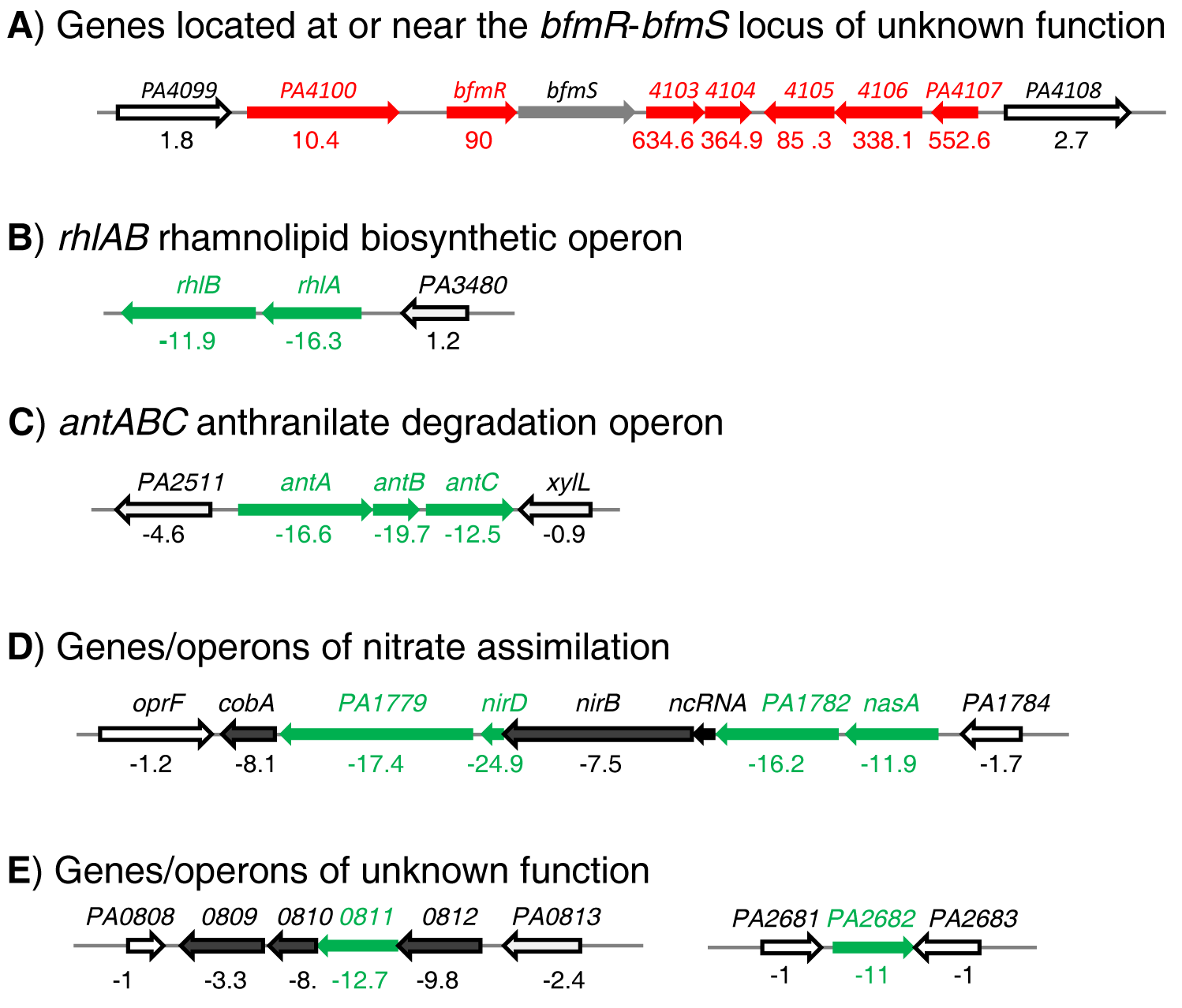

Supplement: Figure S2 — Schematic presentation of genes whose expression is dramatically (>10-fold) affected by the deletion of bfmS . The fold change indicates gene expression levels in ΔbfmS strain compared to expression in the wild-type MPAO1 strain. Genes with more than a 10-fold up- and down-regulation are highlighted in red and green, respectively. A) Genes located at or near the bfmR-bfmS locus of unknown function. B) rhlAB rhamnolipid biosynthetic operon. C) antABC anthranilate degradation operon. D) Genes/operons of nitrate assimilation. E) Genes/operons of unknown function. (TIF) [file ppat.1004340.s002.tif]

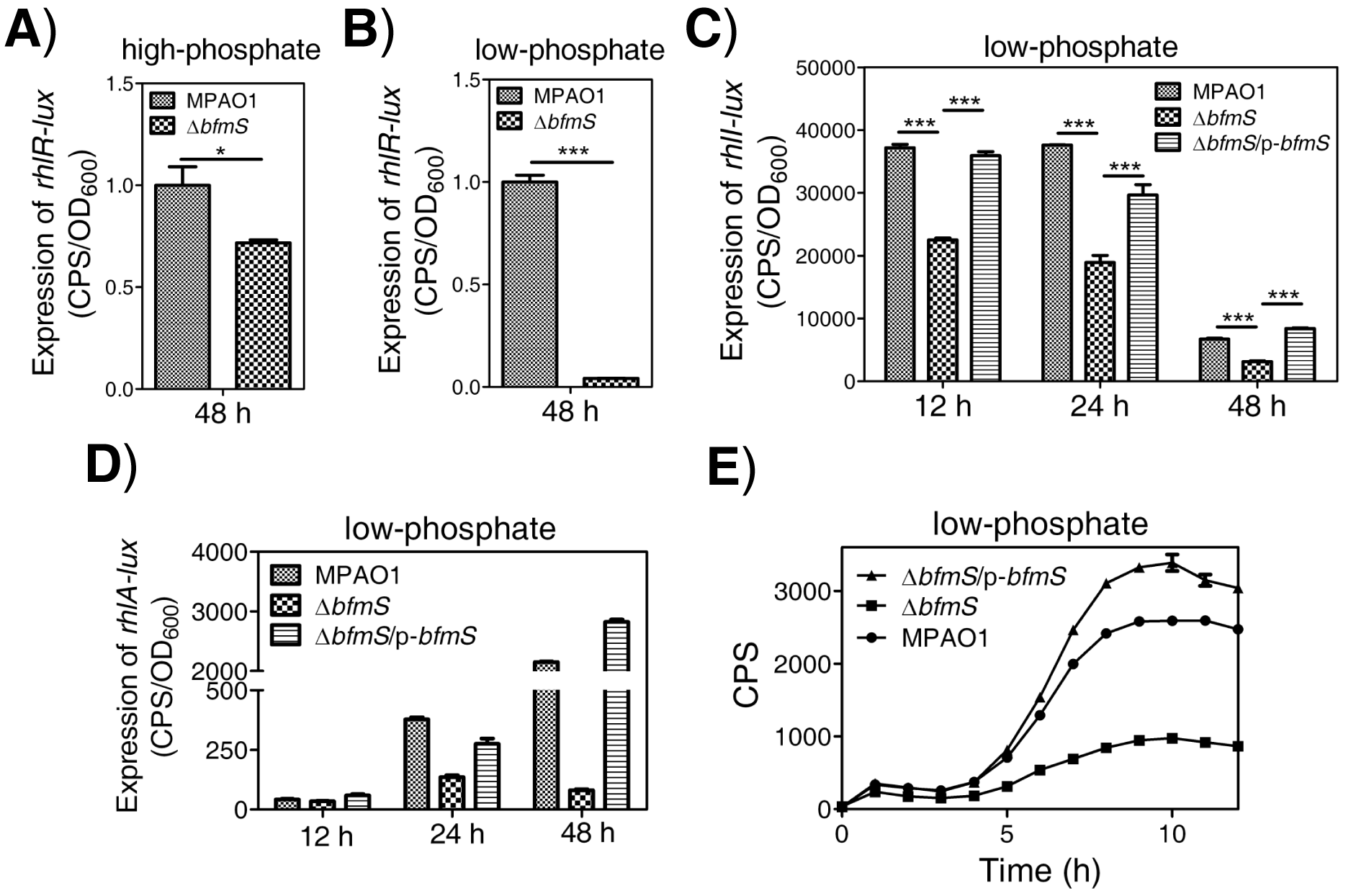

Supplement: Figure S3 — Effect of bfmS deletion on the promoter activities of rhlR , rhlI , and rhlA , and the C4-HSL contents. In panels of C), D), and E), MPAO1 and ΔbfmS harbor plasmid PAK1900, respectively. A) Expression of rhlR-lux in MPAO1 and ΔbfmS mutant when bacteria were grown in M8-glutamate minimal medium (57.6 mM Pi) supplemented with 0.2% glucose at 37°C for 48 h with shaking (250 rpm). B) Expression of rhlR-lux in MPAO1 and ΔbfmS mutant when bacteria were grown low-phosphate (0.32 mM Pi) M8-glutamate minimal medium supplemented with 0.2% glucose at 37°C for 48 h with shaking (250 rpm). C) and D) Expression of rhlI-lux in MPAO1 and its derivatives when bacteria were grown low-phosphate (0.32 mM Pi) M8-glutamate minimal medium supplemented with 0.2% glucose at 37°C with shaking (250 rpm), as indicated. E) Relative amount of C4-HSL measured by the pDO100 (pKD-rhlA) system. Bacteria were grown low-phosphate (0.32 mM Pi) M8-glutamate minimal medium supplemented with 0.2% glucose at 37°C for 48 h with shaking (250 rpm). All experiments were independently repeated at least three times and the data shown represent comparable results. Values represent means ± standard error of the mean (SEM). (TIF) [file ppat.1004340.s003.tif]

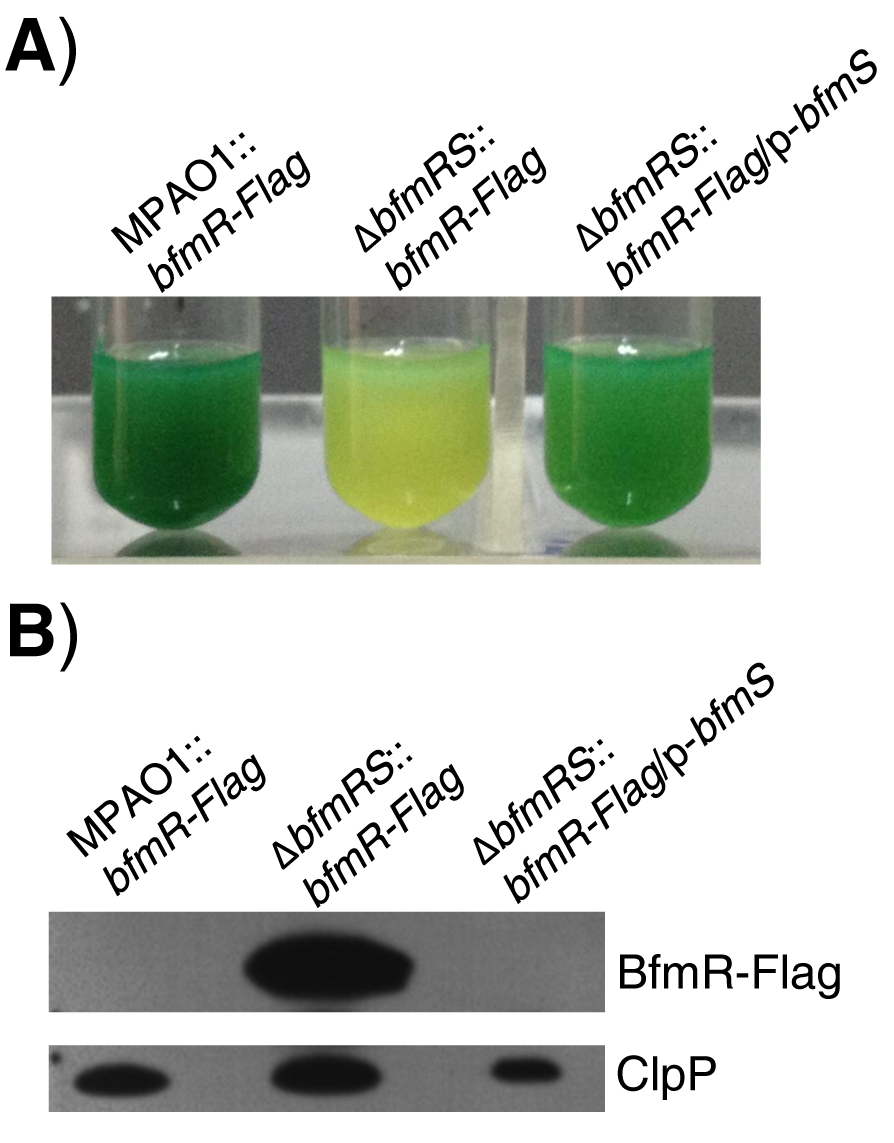

Supplement: Figure S4 — bfmR is activated in the absence of bfmS . In all panels, MPAO1::BfmR-Flag and ΔbfmRS::BfmR-Flag strain harbor the plasmid PAK1900. A) The absence of bfmS results in a greatly decreased pyocyanin production. Bacteria were grown for 24 h in PPB medium at 37°C with shaking (250 rpm). The presence of the blue-green pigment indicates pyocyanin production. B) Western blot analysis showing that the BfmR is significantly elevated in the absence of bfmS. ClpP protein is used as an internal control for loading error as described in the Materials and Methods section. Strains were grown in PPB medium with the appropriate antibiotic at 37°C for 24 h with shaking (250 rpm). The assays were repeated at least three times with similar results obtained. (TIF) [file ppat.1004340.s004.tif]

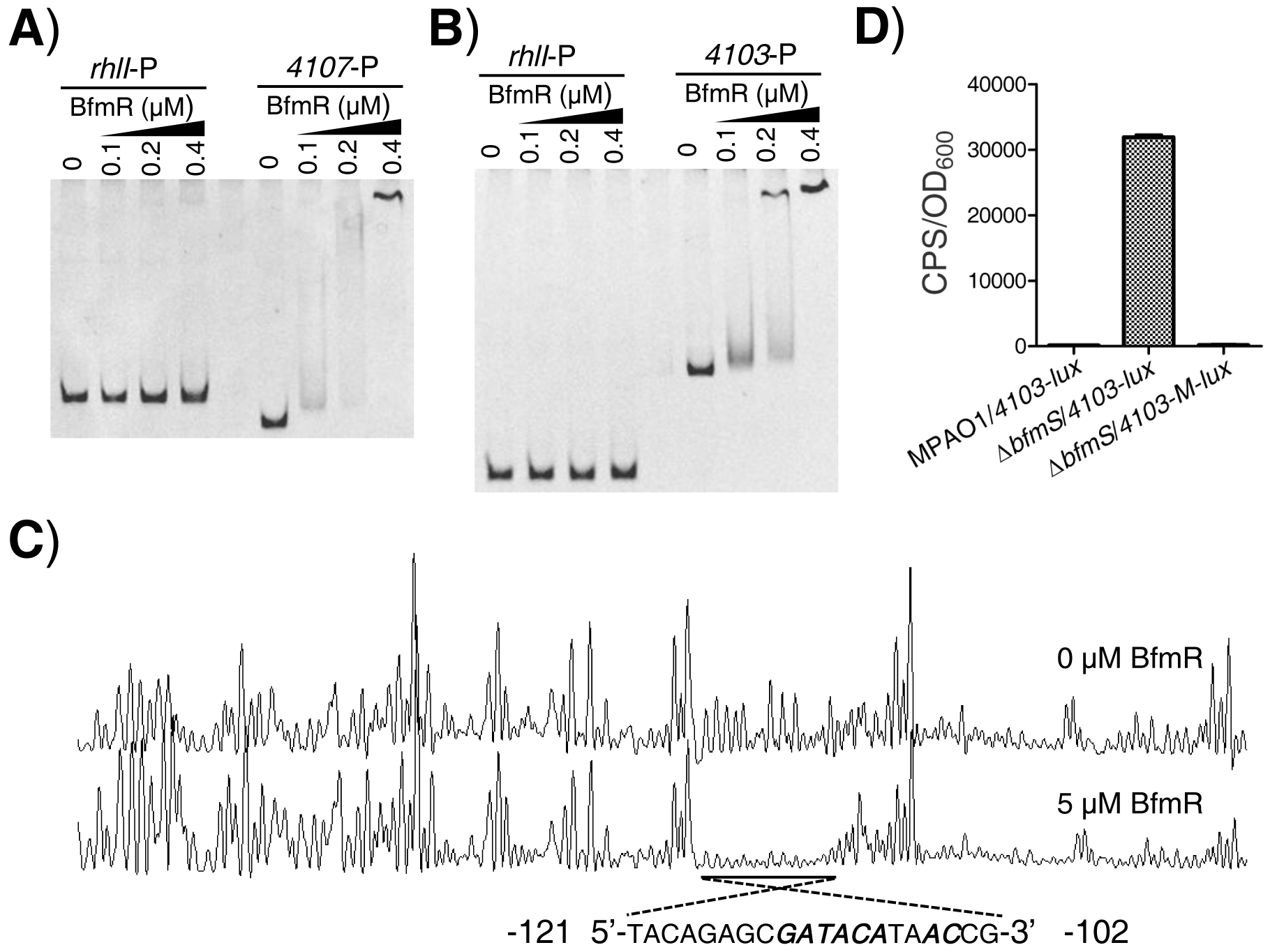

Supplement: Figure S5 — BfmR regulates the expression of PA4107 and PA4103 in a direct manner. A) and B) EMSA shows that 6His-BfmR directly binds to the promoter DNA of PA4107 and PA4103 but not to that of rhlI, as indicated. C) Electropherograms show the protection pattern of the PA4103 promoter DNA after digestion with DNase I following incubation in the absence or the presence of 6His-BfmR. BfmR-protected regions I harbors a putative BfmR-binding motif, which was highlighted in bold and in italics. D) The expression of 4103-lux and 4103-M-lux in wild-type MPAO1 or in the ΔbfmS strain, as indicated. Bacteria were grown in M8-glutamate minimal medium supplemented with 2% glucose at 37°C for 24 h. Values represent means ± SEM. The assays were independently repeated at least three times and the data shown represent comparable results. (TIF) [file ppat.1004340.s005.tif]

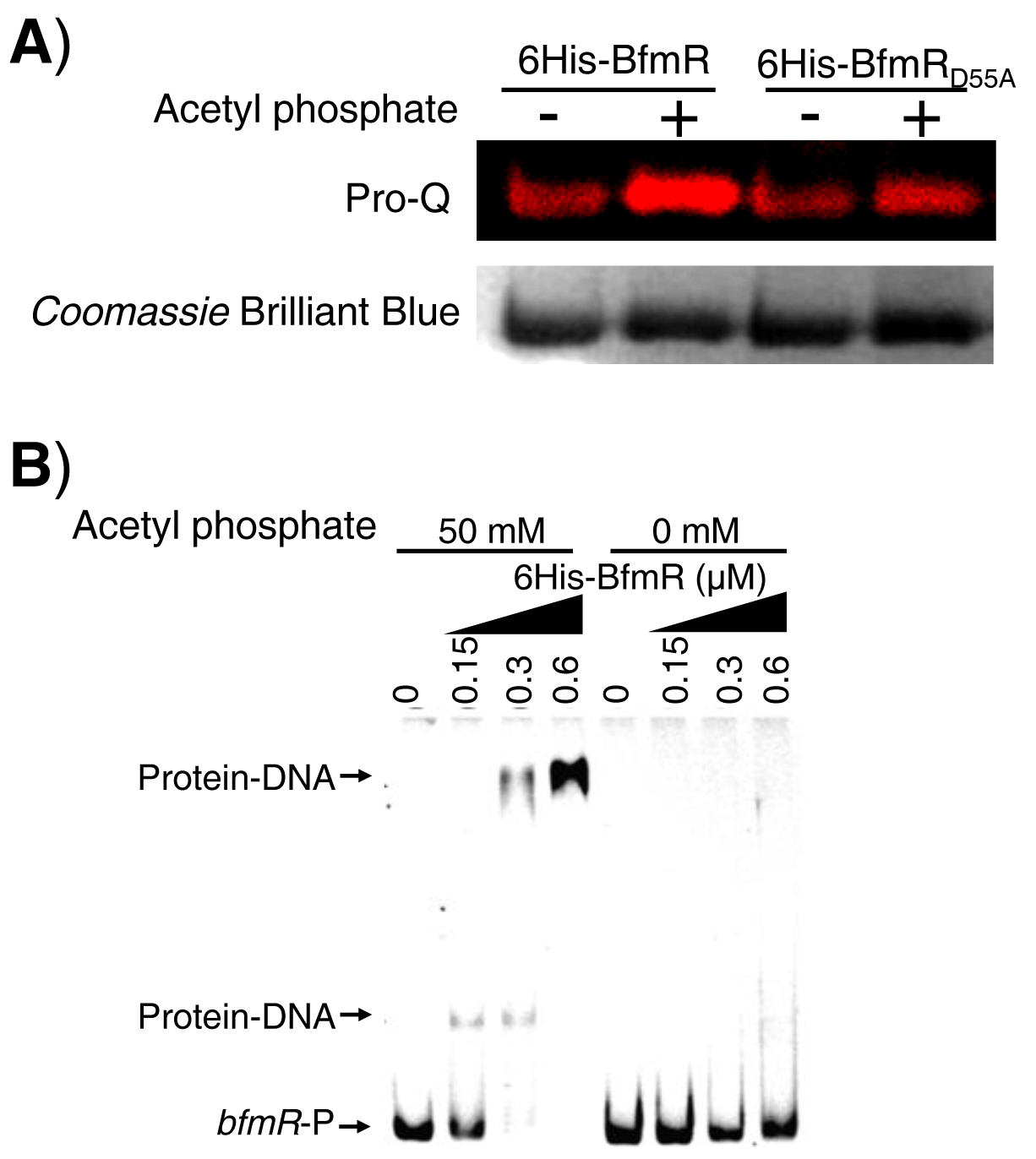

Supplement: Figure S6 — BfmR can be phosphorylated and activated by acetyl phosphate in vitro . A) In vitro phosphorylation assays showing that 6His-BfmR but not 6His-BfmRD55A could be phosphorylated by acetyl phosphate. Phosphorylated protein was detected by the Pro-Q Diamond phosphoprotein gel stain technique (upper panel), while total protein was visualized with the Coomassie brilliant blue stain (lower panel). Wild type (6His-BfmR) and the variant with the amino acid substitution of aspartate for alanine at amino acid 55 (6His-BfmRD55A) were purified by metal affinity chromatography. 2 µM of purified proteins was incubated in the absence (−) or presence (+) of 50 mM acetyl phosphate. Samples were separated in a conventional SDS 12% polyacrylamide gel. B) EMSA showing that acetyl phosphate enhances the DNA-binding ability of 6His-BfmR. The dissociation constants of 6His-BfmR to the bfmR promoter DNA: Kd = ∼0.2 µM, in the presence of acetyl phosphate (50 mM); Kd>0.6 µM, in the absence of acetyl phosphate. All experiments were repeated at least three times with similar results obtained. (TIF) [file ppat.1004340.s006.tif]

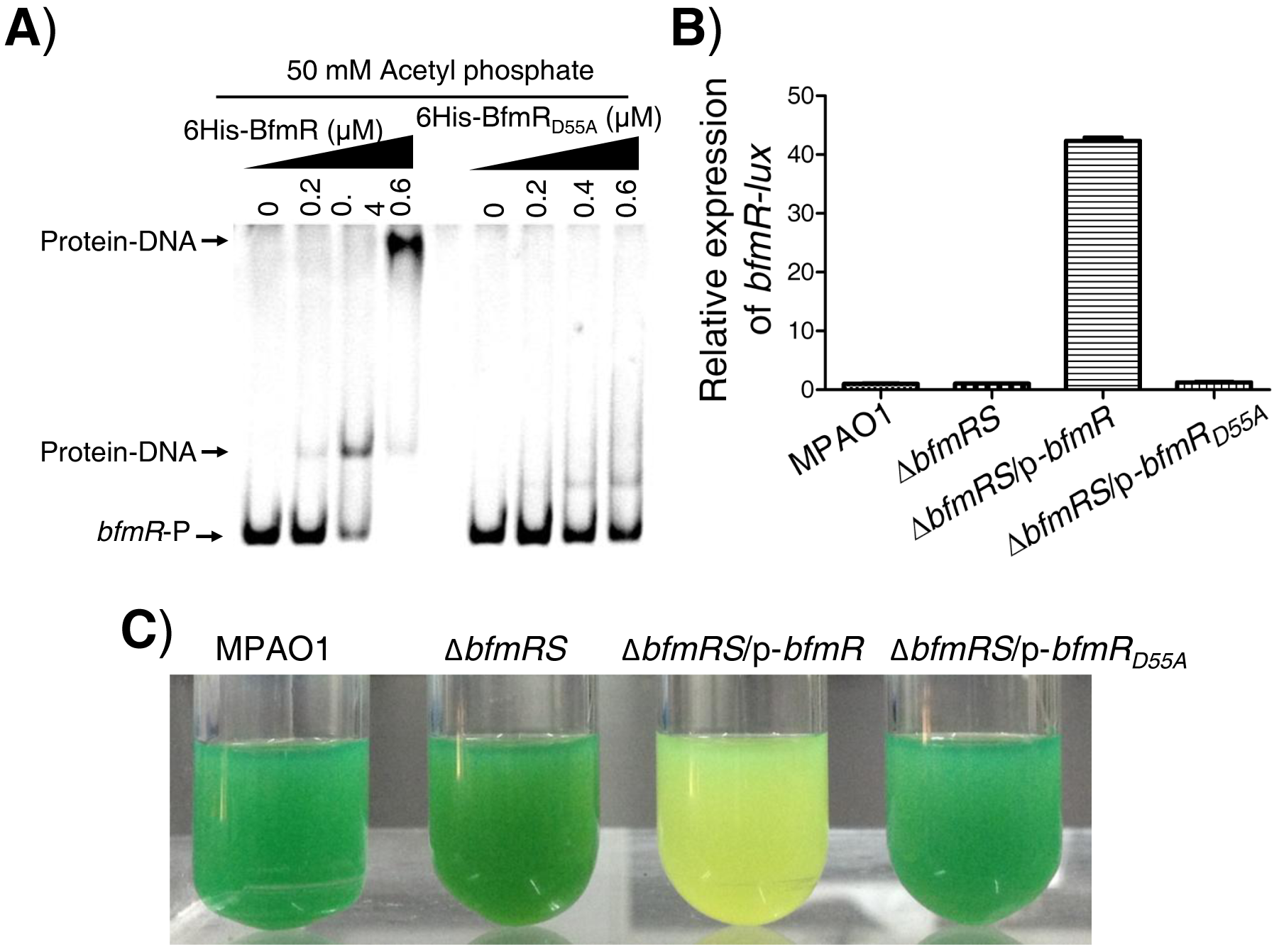

Supplement: Figure S7 — Different experiments indicate that aspartate residue D55 is required in order to activate BfmR. A) EMSA showing that mutation of aspartate residue 55 to alanine attenuates the binding abilities of BfmR to its own promoter. Dissociation constants, Kd (6His-BfmR) ≤0.4 µM, Kd (6His-BfmRD55A) >0.6 µM, were determined by a densitometry analysis. B) Promoter reporter assays showing that mutation of aspartate residue 55 to alanine abolishes the ability of BfmR to induce bfmR-lux activity in ΔbfmRS strain. Bacteria were grown in M8-glutamate minimal medium supplemented with 0.2% glucose at 37°C for 24 h with shaking (250 rpm). Values are relative to MPAO1 (set to 1). Values represent means ± SEM. C) Phenotypic analysis showing that substitution of aspartate for alanine at amino acid 55 abolishes the ability of BfmR to repress the green pigment production of the ΔbfmRS strain. Bacteria were grown in PPB medium at 37°C for 24 h with shaking (250 rpm). All experiments were repeated at least twice with similar results obtained. In B) and C), MPAO1 and ΔbfmRS harbor plasmid PAK1900, respectively. (TIF) [file ppat.1004340.s007.tif]

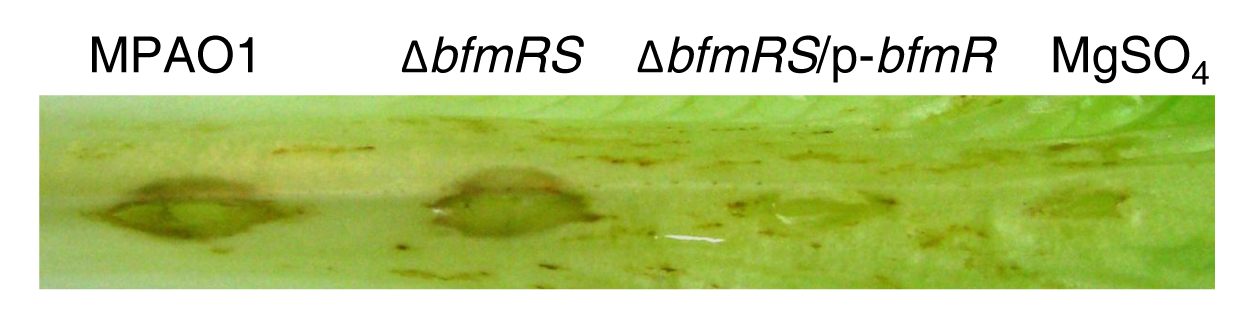

Supplement: Figure S8 — BfmR regulates bacterial virulence. In all panels, MPAO1 and ΔbfmRS strains harbor plasmid PAK1900. Photographs show lettuce midribs after three days of infection with 1×10^7^ cfu of P. aeruginosa. ΔbfmRS strain exhibits a virulence phenotype similar to that of a wild-type MPAO1 strain, while the introduction of p-bfmR into the ΔbfmRS strain leads to a low virulence phenotype. The assays were independently repeated at least three times and the data shown represent comparable results. (TIF) [file ppat.1004340.s008.tif]

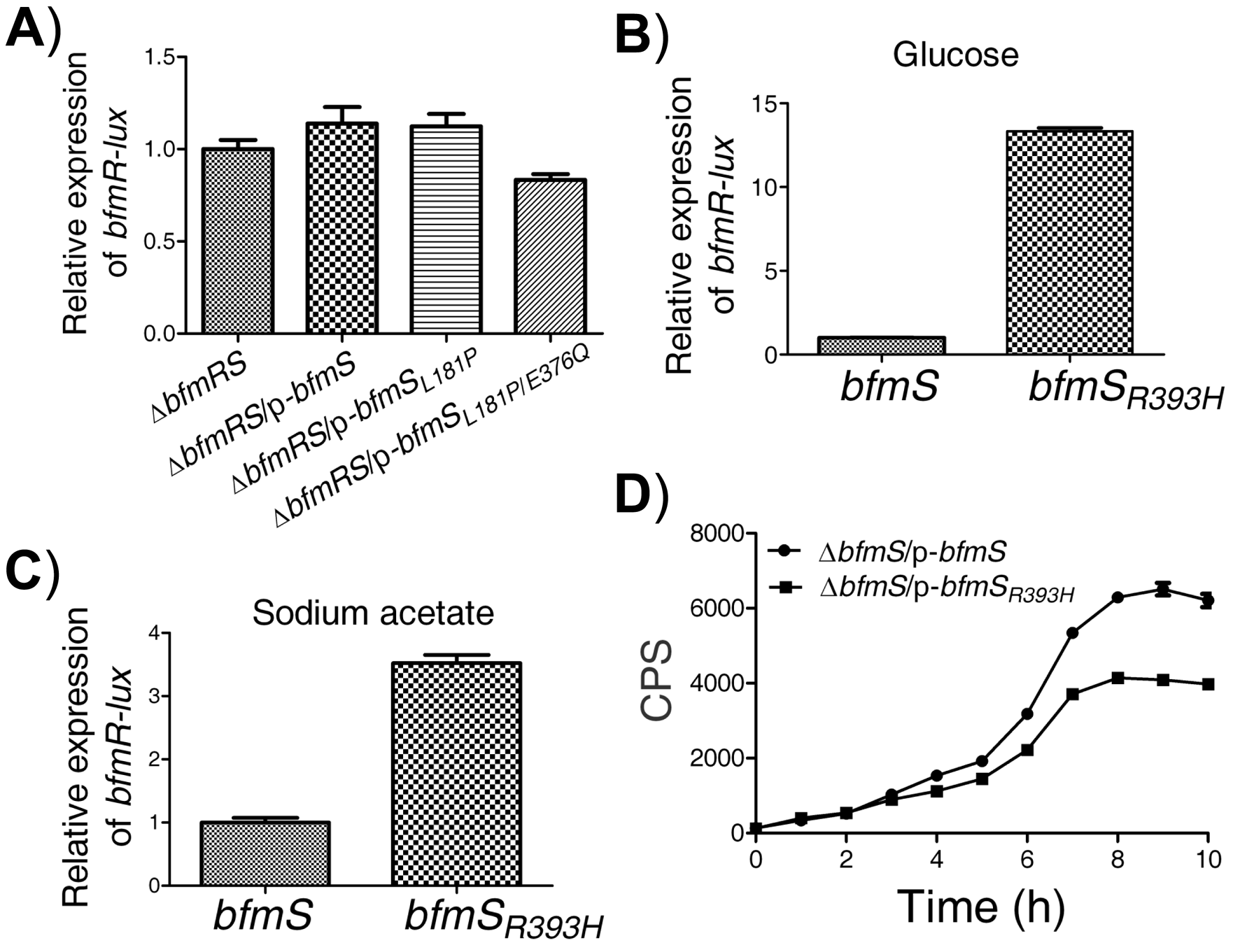

Supplement: Figure S9 — Effects of amino acid substitutions in BfmS on the activation of BfmR. A) Expressing bfmS and its derivatives on the bfmR-lux activity in ΔbfmRS strain. ΔbfmRS strain harbors plasmid PAK1900. Bacteria were grown in M8-glutamate minimal medium supplemented with 0.082% sodium acetate at 37°C for 36 h with shaking (250 rpm). Values are relative to ΔbfmRS strain harboring PAK1900 (set to 1). B) The relative bfmR-lux activity in ΔbfmS/p-bfmS strain (bfmS) and ΔbfmS/p-bfmSR393H strain (bfmSR393H) when bacteria were grown in M8-glutamate minimal medium supplemented with 0.2% glucose at 37°C for 36 h with shaking (250 rpm). Values are relative to ΔbfmS/p-bfmS strain (set to 1). C) The relative bfmR-lux activity in ΔbfmS/p-bfmS strain (bfmS) and ΔbfmS/p-bfmSR393H strain (bfmSR393H) when bacteria were grown in M8-glutamate minimal medium supplemented with 0.082% sodium acetate at 37°C for 36 h with shaking (250 rpm). Values are relative to ΔbfmS/p-bfmS strain (set to 1). D) Relative amount of C4-HSL measured by the pDO100 (pKD-rhlA) system. ΔbfmS/p-bfmS strain (bfmS) and ΔbfmS/p-bfmSR393H strain (bfmSR393H) were grown in M8-glutamate minimal medium supplemented with 0.2% glucose at 37°C for 36 h with shaking (250 rpm). Supernatants were subsequently prepared and measured for relative C4-HSL contents. The assays were independently repeated at least three times, and the data shown are representative of comparable results. Values represent means ± SEM. CPS, counts per second. (TIF) [file ppat.1004340.s009.tif]

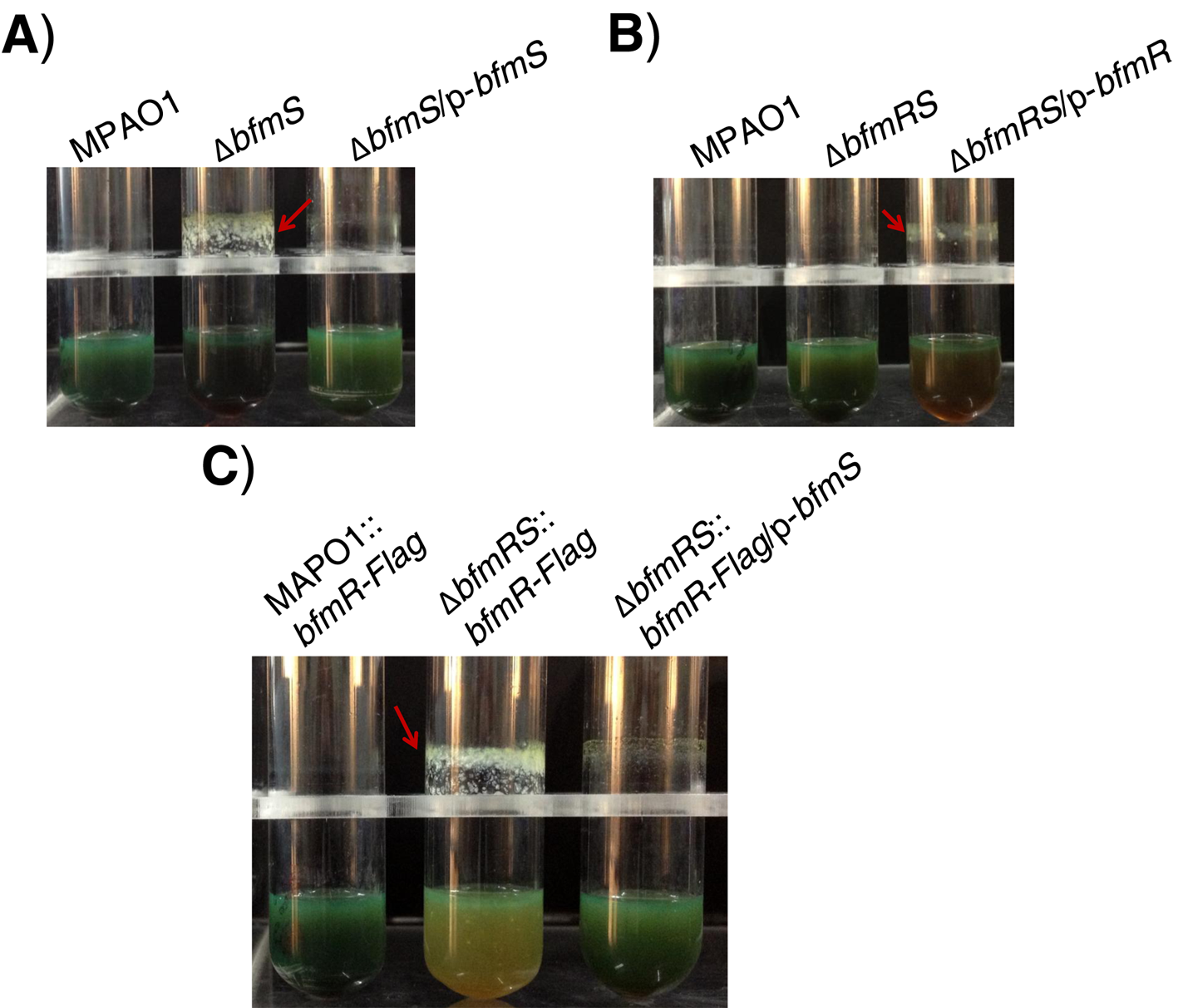

Supplement: Figure S10 — Effect of bfmRS on the biofilm formation of P. aeruginosa . In all panels, MPAO1, ΔbfmS, ΔbfmRS, MPAO1::BfmR-Flag and the ΔbfmRS::BfmR-Flag strain harbor plasmid PAK1900, respectively. MPAO1 and its derivatives were grown for five days in PPB medium at 37°C with shaking (250 rpm). Deletion of bfmS A), expressing either bfmR B) or bfmR-Flag C) in ΔbfmRS strain results in a greatly induced ring of biofilm at the air-liquid interface in shaken liquid cultures, as indicated by the arrow. The assays were independently repeated at least three times, and the data shown are representative of comparable results. (TIF) [file ppat.1004340.s010.tif]

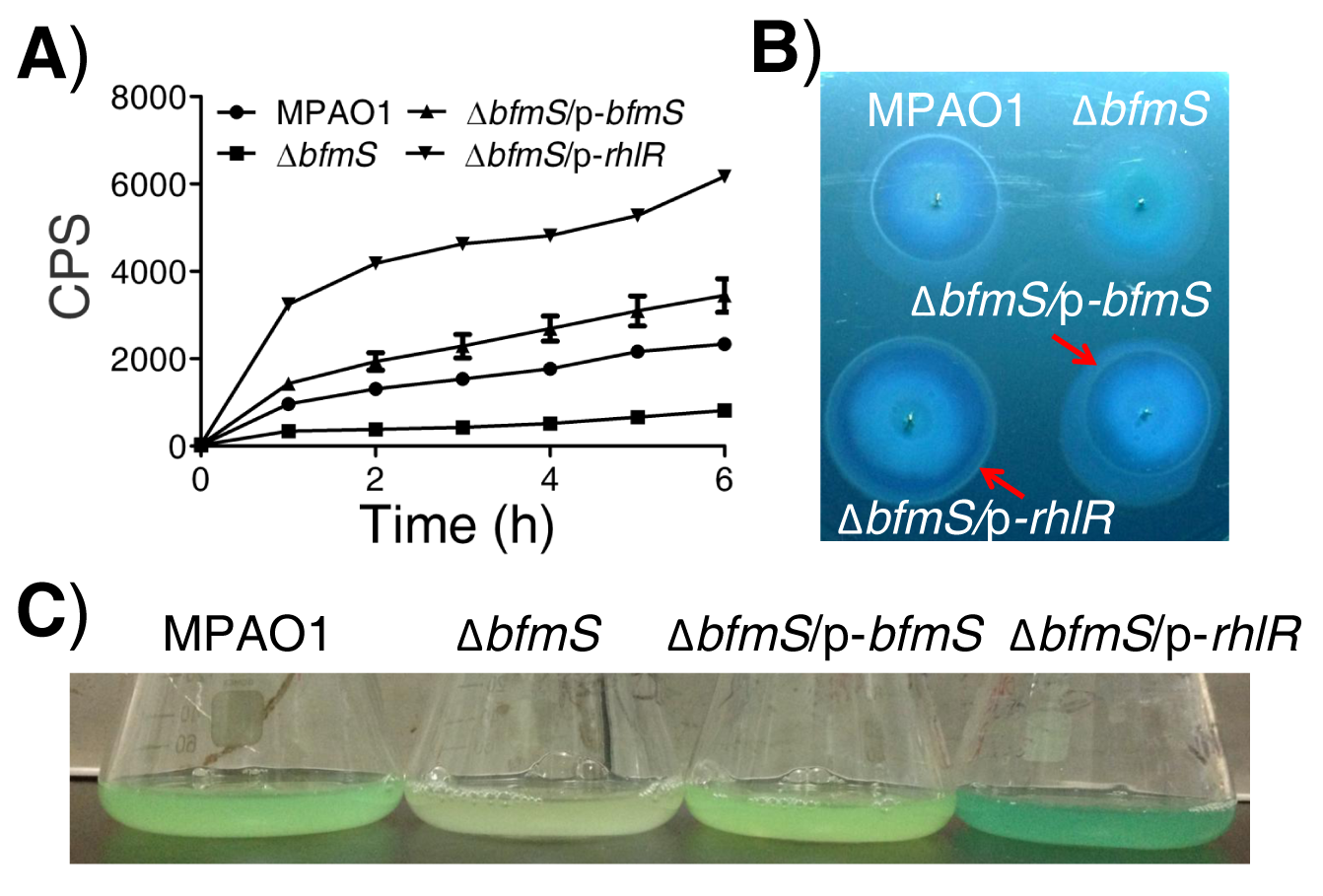

Supplement: Figure S11 — Effect of expressing rhlR in Δ bfmS strain on the production of QS signal C4-HSL, rhamnolipid and pyocyanin. A) Relative amount of C4-HSL measured by the pDO100 (pKD-rhlA) system. MPAO1 and its derivatives were grown in M8-glutamate minimal medium supplemented with 0.2% glucose at 37°C for 48 h with shaking (250 rpm). Supernatants were subsequently prepared and measured for the relative C4-HSL contents. B) Bacterial strains were inoculated onto a cetyltrimethylammonium bromide (CTAB) plate and incubated at 37°C for 24 h and then for 72 h at room temperature; the presence of a blue halo surrounding the colonies indicates production of rhamnolipids. C) MPAO1 and its derivatives were grown in Pyocyanin production broth (PPB) medium at 37°C for 24 h with shaking (250 rpm); the presence of the blue-green pigment indicates pyocyanin production. The assays were independently repeated at least three times, and the data shown are representative of comparable results. Values represent means ± SEM. CPS, counts per second. (TIF) [file ppat.1004340.s011.tif]
