# Supplementary material for: A Novel Signal Transduction Pathway that Modulates rhl Quorum Sensing and Bacterial Virulence in Pseudomonas aeruginosa
Source: PLoS Pathog. 2014 Aug 28;10(8):e1004340. doi: 10.1371/journal.ppat.1004340 (PMC4148453; doi:10.1371/journal.ppat.1004340)
Supplement: Text S1 — contains six Supplemental Tables (Table S1–6). Table S1. Plasmids and bacterial strains used in this study; Table S2. 131 genes whose expressions are up-regulated more than 2-fold in ΔbfmS strain compared to wild-type MPAO1 strain; Table S3. 71 genes whose expression are down-regulated more than 2-fold in ΔbfmS strain compared to wild-type MPAO1 strain; Table S4. Verification of microarray results by Real-Time RT-PCR; Table S5. 41 promoters identified by consensus sequence search; Table S6. Primers used in this study. (DOCX) [file ppat.1004340.s012.docx]

**Supporting Information:**

# **A Novel Signal Transduction Pathway that Modulates *rhl* Quorum Sensing and Bacterial Virulence in *Pseudomonas aeruginosa***

Qiao Cao^1,2,#^, Yue Wang^2,3,#^, Feifei Chen^2^, Yongjie Xia^2^, Jingyu Lou^2^, Xue Zhang^2^, Nana Yang^2^, Xiaoxu Sun^2^, Qin Zhang^4^, Chao Zhuo^4^, Xi Huang^1^, Xin Deng^6^, Cai-Guang Yang^2^, Yan Ye^5^, Jing Zhao^3,*^, Min Wu^5,*^, Lefu Lan^2,*^

^1^Hainan University, Haikou, Hainan 570228, China

^2^Shanghai Institute of Materia Medica, Chinese Academy of Sciences, 555 Zuchongzhi Road, Pudong Zhangjiang Hi-Tech Park, Shanghai 201203, China

^3^Institute of Chemistry and BioMedical Sciences, State Key Laboratory of Pharmaceutical Biotechnology, School of Life Sciences, Nanjing University, Nanjing 210093, China

^4^State Key Laboratory of Respiratory Diseases and the First Affiliated Hospital of Guangzhou Medical College, Guangzhou 510182, China

^5^Department of Basic Sciences, University of North Dakota School of Medicine and Health Sciences, Grand Forks, ND 58203, USA

^6^Department of Chemistry and Institute for Biophysical Dynamics, The University of Chicago, Chicago, Illinois 60637, USA

^#^Contributed equally.

*E.mail: [jingzhao@nju.edu.cn](mailto:jingzhao@nju.edu.cn) (JZ); [min.wu@med.und.edu](mailto:min.wu@med.und.edu) (MW); [llan@simm.ac.cn](mailto:llan@simm.ac.cn) (LL)

**Running Title:** **Regulation of *rhl* QS by BfmS in *P. aeruginosa***

**Text S1:**

**Table S1. Plasmids and bacterial strains and used in this study**

| Plasmids or strains | Relevant characteristics^a^ | Source |
| --- | --- | --- |
| Plasmids |  |  |
| PAK1900 | *E. coli–P. aeruginosa* shuttle cloning vector, Ap^r^ Cb^r^ | [[1](#_ENREF_1)] |
| pEX18Ap | Gene replacement vector, mob^+^*sacB*, Ap^r^ | [[2](#_ENREF_2)] |
| pPS858 | pBR322 derivative carrying a FRT-Gm cassette, Ap^r^ | [[2](#_ENREF_2)] |
| pET28a | T7 *lac* promoter–operator, N-terminal His tag, kan^r^ | Novagen |
| pET28b | T7 *lac* promoter–operator, N-terminal His tag, kan^r^ | Novagen |
| mini-CTX-lacZ | Gene delivery vector for inserting genes at the CTX phage *att* site on *P. aeruginosa* chromosome, Tc^r^ | [[3](#_ENREF_3)] |
| pMS402 | *lux-*based promoter reporter plasmid, Km^r^ Tp^r^ | [[4](#_ENREF_4)] |
| pKD-*rhlA* | pMS402 containing *rhlA* promoter region | [[4](#_ENREF_4)] |
| p-*bfmS* | PAK1900 derivative carrying *bfmS* (*PA4102*) on a *c*. 1.4 kb *Hin*dIII/*Bam*HI fragment in same orientation as p*lac* | This study |
| p-*bfmR* | PAK1900 derivative carrying *bfmR* (*PA4101*) on a *c*. 0.8 kb *Hin*dIII/*Bam*HI fragment in same orientation as p*lac* | This study |
| p-*bfmRS* | PAK1900 derivative carrying *bfmRS* (*PA4101-PA4102*) on a *c*. 2.0 kb *Hin*dIII/*Bam*HI fragment in same orientation as p*lac* | This study |
| p-*bfmS_L181P_* | p-*bfmS* derivative carrying proline substitution mutant at the site leucine 181 | This study |
| p-*bfmS_E376Q_* | p-*bfmS* derivative carrying glutamine substitution mutant at the site glutamic acid 376 | This study |
| p-*bfmS_L181P/E376Q_* | p-*bfmS* derivative carrying both L181P and E376Q substitutions | This study |
| p-*bfmR_D55A_* | p-*bfmR* derivative carrying alanine substitution mutant at the site of aspartate residue 55 | This study |
| p-*rhlR* | PAK1900 derivative carrying *rhlR* gene on a *c*. 0.8 kb *Hin*dIII fragment in same orientation as p*lac* | This study |
| p-*acka-pta* | PAK1900 derivative carrying *rhlR* gene on a *c*. 3.5 kb *Hin*dIII/*Bam*HI fragment in same orientation as p*lac* | This study |
| pEX18Ap::*bfmS*UGD | pEX18Ap derivative, for replacing MPAO1 *bfmS* gene with a gentamicin resistance cassette from plasmid pPS858 | This study |
| pEX18Ap::*bfmRS*UGD | pEX18Ap derivative, for replacing MPAO1 *bfmRS* loci with a gentamicin resistance cassette from plasmid pPS858 | This study |
| pEX18Ap::*acka-pta*UTD | pEX18Ap derivative, for replacing *acka-pta* loci with a tetracycline resistance cassette from plasmid mini-CTX-lacZ | This study |
| pET28a-6His-BfmR | pET28a derivative carrying *bfmR*(*PA4101*) | This study |
| pET28a-6His-BfmR_D55A_ | pET28a derivative carrying *bfmR*(*PA4101*) which has alanine substitution mutant at the site of aspartate residue 55 | This study |
| pET28b-*bfmS_34-154_* | pET28a derivative carrying *bfmS_34-154_* (encoding residues 34-154 of BfmS, designated *bfmS_34-154_*) | This study |
| mini-ctx-BfmR-Flag | mini-CTX-lacZ derivative carrying *bfmR-Flag* | This study |
| *rhlA-lux* | pMS402 containing *rhlA* promoter region (from -526 to -20 of the start codon) | This study |
| *bfmR*-*lux* | pMS402 containing *bfmR* promoter region | This study |
| *rhlR-lux* | pMS402 containing *rhlR* promoter region (from −450 to +19 of the start codon) | This study |
| *rhlR-D-lux* | pMS402 containing *rhlR* promoter region that lacks the putative BfmR-binding site (GATACT) | This study |
| *4103-lux* | pMS402 containing *PA4103* promoter region (from −420 to +19 of the start codon) | This study |
| *4103-M-lux* | pMS402 containing mutated *PA4103* promoter region (from −420 to +19 of the start codon, GATACA was mutated to ATATAT) | This study |
| pKD-*rhlI* | pMS402 containing *rhlI* promoter region | [[5](#_ENREF_5)] |
| Strains |  |  |
| MPAO1 | Wild type | [[6](#_ENREF_6)] |
| pDO100 (pKD-*rhlA*) | *rhlI* mutant of PAO1 harboring plasmid pKD-*rhlA* | [[5](#_ENREF_5)] |
| Δ*bfmS* | MPAO1 derivative with a gentamicin resistance cassette replaced the *bfmS* gene | This study |
| Δ*bfmRS* | MPAO1 derivative with a gentamicin resistance cassette replaced the *bfmRS* locus | This study |
| MPAO1/PAK1900 | MPAO1 carrying plasmid PAK1900 | This study |
| Δ*bfmS*/PAK1900 | Δ*bfmS* carrying plasmid PAK1900 | This study |
| Δ*bfmRS*/PAK1900 | Δ*bfmRS* carrying plasmid PAK1900 | This study |
| Δ*bfmS*/p-*bfmS* | Δ*bfmS* carrying plasmid p-*bfmS* | This study |
| Δ*bfmRS*/p-*bfmR* | Δ*bfmRS* carrying plasmid p-*bfmR* | This study |
| Δ*bfmRS*/p-*bfmS* | Δ*bfmRS* carrying plasmid p-*bfmS* | This study |
| Δ*bfmRS*/p-*bfmRS* | Δ*bfmRS* carrying plasmid p-*bfmRS* | This study |
| MPAO1/PAK1900 + *bfmR*-*lux* | MPAO1 carrying plasmids of PAK1900 and *bfmR*-*lux* | This study |
| Δ*bfmS*/PAK1900 + *bfmR*-*lux* | Δ*bfmS* carrying plasmids of PAK1900 and *bfmR*-*lux* | This study |
| Δ*bfmRS*/PAK1900 + *bfmR*-*lux* | Δ*bfmRS* carrying plasmids of PAK1900 and *bfmR*-*lux* | This study |
| Δ*bfmS*/p-*bfmS* + *bfmR*-*lux* | Δ*bfmS* carrying plasmids of p-*bfmS* and *bfmR*-*lux* | This study |
| Δ*bfmRS*/p-*bfmR* + *bfmR*-*lux* | Δ*bfmRS* carrying plasmids of p-*bfmR* and *bfmR*-*lux* | This study |
| Δ*bfmRS*/p-*bfmS* + *bfmR*-*lux* | Δ*bfmRS* carrying plasmids of p-*bfmS* and *bfmR*-*lux* | This study |
| Δ*bfmRS*/p-*bfmRS*+ *bfmR*-*lux* | Δ*bfmRS* carrying plasmids of p-*bfmRS* and *bfmR*-*lux* | This study |
| MPAO1/PAK1900 + *rhlR-lux* | MPAO1 carrying plasmids of PAK1900 and *rhlR-lux* | This study |
| Δ*bfmS*/PAK1900 + *rhlR-lux* | Δ*bfmS* carrying plasmids of PAK1900 and *rhlR-lux* | This study |
| Δ*bfmRS*/PAK1900 + *rhlR-lux* | Δ*bfmRS* carrying plasmids of PAK1900 and *rhlR-lux* | This study |
| Δ*bfmS*/p-*bfmS* + *rhlR-lux* | Δ*bfmS* carrying plasmids of p-*bfmS* and *rhlR-lux* | This study |
| Δ*bfmRS*/p-*bfmR* + *rhlR-lux* | Δ*bfmRS* carrying plasmids of p-*bfmR* and *rhlR-lux* | This study |
| Δ*bfmS* /p-*rhlR* | Δ*bfmS* carrying plasmids of p-*rhlR* | This study |
| MPAO1::BfmR-Flag | Strain MPAO1 with genes *bfmR-flag* and *lacZ* inserted at the CTX phage *att* site on the chromosome | This study |
| Δ*bfmRS*::*BfmR*-*Flag* | Strain Δ*bfmRS* with genes *bfmR-flag* and *lacZ* inserted at the CTX phage *att* site on the chromosome | This study |
| MPAO1::BfmR-Flag/PAK1900 | MPAO1::BfmR-Flag carrying plasmid PAK1900 | This study |
| Δ*bfmRS*::*BfmR*-*Flag*/PAK1900 | Δ*bfmRS*::*BfmR*-*Flag* carrying plasmid PAK1900 | This study |
| Δ*bfmRS*::*BfmR*-*Flag*/p-*bfmS* | Δ*bfmRS*::*BfmR*-*Flag* carrying plasmid p-*bfmS* | This study |
| Δ*bfmS* /p-*bfmS_L181P_* | Δ*bfmS* carrying plasmid p-*bfmS_L181P_* | This study |
| Δ*bfmS* /p-*bfmS_E376Q_* | Δ*bfmS* carrying plasmid p-*bfmS_E376Q_* | This study |
| Δ*bfmS* /p-*bfmS_L181P/E376Q_* | Δ*bfmS* carrying plasmid p-*bfmS_L181P/E376Q_* | This study |
| Δ*bfmS* /p-*bfmS_L181P_* + *bfmR-lux* | Δ*bfmS* carrying plasmids of p-*bfmS_L181P_* and *bfmR-lux* | This study |
| Δ*bfmS* /p-*bfmS_E376Q_* + *bfmR-lux* | Δ*bfmS* carrying plasmid p-*bfmS_E376Q_* and *bfmR-lux* | This study |
| Δ*bfmS* /p-*bfmS_L181P/E376Q_* + *bfmR-lux* | Δ*bfmS* carrying plasmid p-*bfmS_L181P/E376Q_* and *bfmR-lux* | This study |
| DH5a | *endA hsdR17 supE44 thi-1 recA1 gyrA relA1*Δ(*lacZYA-argF*)*U169 deoR* (*φ80dlac*Δ(*lacZ*)*M15*) | Lab stock |
| BL21 | F^–^*ompT hsdS*_B_ (r_B_^-^ m_B_^-^) *gal dcm met* (DE3) | Lab stock |
| S17 λ-pir | *recA thi pro hsdR*^−^*M*^+^ RP4-2-Tc::Mu Km::Tn*7* λpir (Tp^r^ Str^r^) | Lab stock |

^a^ Ap^r^, ampicillin resistance; Cb^r^, carbenicillin resistance; Km^r^, kanamycin resistance; Tc^r^, tetracycline resistance; Tp^r^, trimethoprim resistance; Str^r^, Streptomycin resistance.

**Table S2. 131 genes whose expressions are up-regulated more than 2-fold in Δ*bfmS* strain compared to wild-type MPAO1 strain**

| Gene Id* | Fold Change | q-value | Annotation | Function |
| --- | --- | --- | --- | --- |
| PA4103* | 634.58 | 0.00 | Hypothetical protein | Hypothetical protein |
| PA4107* | 552.59 | 0.00 | Hypothetical protein | Hypothetical protein |
| PA4104 | 364.89 | 0.00 | Hypothetical protein | Hypothetical protein |
| PA4106 | 338.13 | 0.00 | Hypothetical protein | Hypothetical protein |
| PA4101* | 90.06 | 0.00 | Probable two-component response regulator | Regulatory function |
| PA4105 | 85.31 | 0.00 | Hypothetical protein | Hypothetical protein |
| PA4100 | 10.38 | 0.00 | Probable dehydrogenase | Central intermediary metabolism |
| PA4471 | 9.46 | 4.02 | Hypothetical protein | Hypothetical protein |
| PA1178* | 5.98 | 0.00 | Outer membrane protein H1 precursor | Adaptation, protection |
| PA2009 | 5.52 | 0.80 | Homogentisate 1,2-dioxygenase | Carbon compound catabolism |
| PA4714* | 4.62 | 0.37 | Hypothetical protein | Hypothetical protein |
| PA2381* | 4.22 | 0.90 | Hypothetical protein | Hypothetical protein |
| PA0872 | 3.83 | 0.21 | Phenylalanine-4-hydroxylase | Amino acid biosynthesis and metabolism |
| PA5446* | 3.83 | 2.17 | Hypothetical protein | Hypothetical protein |
| PA2190* | 3.61 | 0.45 | Hypothetical protein | Hypothetical protein |
| PA2146 | 3.58 | 0.00 | Hypothetical protein | Hypothetical protein |
| PA0865 | 3.55 | 3.56 | 4-hydroxyphenylpyruvate dioxygenase | Amino acid biosynthesis and metabolism |
| PA2166 | 3.41 | 1.87 | Hypothetical protein | Hypothetical protein |
| PA0567 | 3.40 | 0.68 | Hypothetical protein | Hypothetical protein |
| PA5482 | 3.28 | 0.45 | Hypothetical protein | Hypothetical protein |
| PA4739 | 3.26 | 0.45 | Hypothetical protein | Hypothetical protein |
| PA1333 | 3.07 | 1.14 | Hypothetical protein | Hypothetical protein |
| PA4738 | 3.05 | 0.49 | Hypothetical protein | Hypothetical protein |
| PA5304 | 3.02 | 0.21 | D-amino acid dehydrogenase, small subunit | Amino acid biosynthesis and metabolism |
| PA5217 | 3.02 | 0.37 | Probable binding protein component of ABC iron transporter | Transport of small molecules |
| PA3274 | 2.92 | 0.00 | Hypothetical protein | Hypothetical protein |
| PA0355* | 2.91 | 0.68 | Protease PfpI | Protein degradation |
| PA3712 | 2.90 | 0.00 | Hypothetical protein | Hypothetical protein |
| PA1179 | 2.88 | 0.00 | Two-component response regulator PhoP | Regulatory function |
| PA2143 | 2.87 | 0.00 | Hypothetical protein | Hypothetical protein |
| PA2008* | 2.83 | 0.00 | Fumarylacetoacetase | Carbon compound catabolism |
| PA4876 | 2.82 | 0.21 | Osmotically inducible lipoprotein OsmE | Adaptation, protection |
| PA1332 | 2.78 | 0.45 | Hypothetical protein | Hypothetical protein |
| PA5212* | 2.75 | 1.66 | Hypothetical protein | Hypothetical protein |
| PA2604 | 2.73 | 0.00 | Hypothetical protein | Hypothetical protein |
| PA4108* | 2.66 | 0.00 | Hypothetical protein | Hypothetical protein |
| PA3575 | 2.64 | 0.45 | Hypothetical protein | Hypothetical protein |
| PA5528 | 2.64 | 0.37 | Hypothetical protein | Hypothetical protein |
| PA2134 | 2.63 | 0.00 | Hypothetical protein | Hypothetical protein |
| PA1053 | 2.62 | 0.21 | Hypothetical protein | Hypothetical protein |
| PA4877 | 2.62 | 0.96 | Hypothetical protein | Hypothetical protein |
| PA5360 | 2.57 | 0.00 | Two-component response regulator PhoB | Regulatory function |
| PA2747* | 2.55 | 3.19 | Hypothetical protein | Hypothetical protein |
| PA0059 | 2.53 | 0.21 | Osmotically inducible protein OsmC | Adaptation, protection |
| PA2985 | 2.48 | 1.14 | Hypothetical protein | Hypothetical protein |
| PA3371* | 2.48 | 4.02 | Hypothetical protein | Hypothetical protein |
| PA3040 | 2.47 | 0.21 | Hypothetical protein | Hypothetical protein |
| PA5124* | 2.46 | 0.21 | Two-component sensor NtrB | Regulatory function |
| PA2433* | 2.45 | 1.14 | Hypothetical protein | Hypothetical protein |
| PA2483 | 2.44 | 0.00 | Hypothetical protein | Hypothetical protein |
| PA2754 | 2.42 | 1.08 | Hypothetical protein | Hypothetical protein |
| PA2184 | 2.38 | 0.00 | Hypothetical protein | Hypothetical protein |
| PA2024 | 2.35 | 0.72 | Probable ring-cleaving dioxygenase | Putative enzymes |
| PA2485 | 2.35 | 2.17 | Hypothetical protein | Hypothetical protein |
| PA1324 | 2.32 | 0.21 | Hypothetical protein | Hypothetical protein |
| PA3056* | 2.30 | 0.00 | Hypothetical protein | Hypothetical protein |
| PA0745 | 2.28 | 4.35 | Probable enoyl-CoA hydratase | Putative enzymes |
| PA1985 | 2.28 | 1.53 | Pyrroloquinoline quinone biosynthesis protein A | Biosynthesis of cofactors |
| PA4623 | 2.28 | 2.17 | Hypothetical protein | Hypothetical protein |
| PA1112 | 2.24 | 0.21 | Hypothetical protein | Hypothetical protein |
| PA5200 | 2.24 | 0.21 | Two-component response regulator OmpR | Regulatory function |
| PA1323 | 2.23 | 0.21 | Hypothetical protein | Hypothetical protein |
| PA0833 | 2.23 | 1.05 | Hypothetical protein | Hypothetical protein |
| PA2830 | 2.22 | 0.21 | Heat shock protein HtpX | Adaptation, protection |
| PA2159* | 2.22 | 0.21 | Hypothetical protein | Hypothetical protein |
| PA0114* | 2.22 | 0.72 | Hypothetical protein | Hypothetical protein |
| PA2857* | 2.21 | 0.45 | ATP-binding component of ABC transporter | Transport of small molecules |
| PA2148* | 2.20 | 2.17 | Hypothetical protein | Hypothetical protein |
| PA1404* | 2.17 | 2.48 | Hypothetical protein | Hypothetical protein |
| PA3691 | 2.17 | 0.37 | Hypothetical protein | Hypothetical protein |
| PA2165 | 2.15 | 0.21 | Probable glycogen synthase | Energy metabolism |
| PA5481 | 2.15 | 4.35 | Hypothetical protein | Hypothetical protein |
| PA4094 | 2.15 | 0.72 | Probable transcriptional regulator | Regulatory function |
| PA3055 | 2.13 | 0.21 | Hypothetical protein | Hypothetical protein |
| PA1180* | 2.12 | 0.49 | Two-component sensor PhoQ | Regulatory function |
| PA3969* | 2.09 | 0.37 | Hypothetical protein | Hypothetical protein |
| PA3786 | 2.08 | 0.80 | Hypothetical protein | Hypothetical protein |
| PA2160 | 2.08 | 0.90 | Probable glycosyl hydrolase | Putative enzymes |
| PA2915 | 2.08 | 0.21 | Hypothetical protein | Hypothetical protein |
| PA0788 | 2.08 | 1.53 | Hypothetical protein | Hypothetical protein |
| PA0805* | 2.08 | 1.05 | Hypothetical protein | Hypothetical protein |
| PA0038 | 2.08 | 0.45 | Hypothetical protein | Hypothetical protein |
| PA4661 | 2.07 | 1.66 | Hypothetical protein | Hypothetical protein |
| PA3788* | 2.06 | 2.17 | Hypothetical protein | Hypothetical protein |
| PA2007 | 2.06 | 3.19 | Maleylacetoacetate isomerase | Carbon compound catabolism |
| PA3042 | 2.05 | 0.21 | Hypothetical protein | Hypothetical protein |
| PA3041* | 2.04 | 0.00 | Hypothetical protein | Hypothetical protein |
| PA3273 | 2.04 | 0.00 | Hypothetical protein | Hypothetical protein |
| PA0777 | 2.03 | 0.37 | Hypothetical protein | Hypothetical protein |
| PA0485 | 2.03 | 0.21 | Hypothetical protein | Hypothetical protein |
| PA0929 | 2.03 | 3.56 | Two-component response regulator | Regulatory function |
| PA5521 | 2.02 | 3.71 | Probable short-chain dehydrogenase | Putative enzymes |
| PA2607 | 2.01 | 2.48 | Hypothetical protein | Hypothetical protein |
| PA0744 | 2.00 | 3.71 | Probable enoyl-CoA hydratase/isomerase | Putative enzymes |

Microarray data were analyzed by using SAM (Significance Analysis of Microarrays) software (15). The criterion of cutoff limitation as a fold change ≥2 or ≤0.5 and q-value ≤5% was used to select differential expression genes. * The promoter (−1 bp to −400 bp of the coding region) harbors a conserved gatacannGC (where n is any nucleotide) DNA sequence without mismatch or with no more than one mismatch.

**Table S3. 71 genes whose expression are down-regulated more than 2-fold in Δ*bfmS* strain compared to wild-type MPAO1 strain**

| Gene Id | Fold Change | q-value | Annotation | Function |
| --- | --- | --- | --- | --- |
| PA1780 | -24.88 | 0.31 | Assimilatory nitrite reductase small subunit (NirD) | Central intermediary metabolism |
| PA2513* | -19.69 | 0.42 | Anthranilate dioxygenase small subunit (AntB) | Carbon compound catabolism |
| PA1779 | -17.36 | 0.31 | Assimilatory nitrate reductase | Central intermediary metabolism |
| PA2512 | -16.56 | 0.31 | Anthranilate dioxygenase large subunit (AntA) | Carbon compound catabolism |
| PA3479 | -16.31 | 0.31 | Rhamnosyltransferase chain A (RhlA) | Secreted Factors |
| PA1782 | -16.21 | 0.00 | Probable serine/threonine-protein kinase | Adaptation, protection |
| PA0811 | -12.72 | 0.00 | Probable MFS transporter | Transport of small molecules |
| PA2514 | -12.52 | 0.00 | Anthranilate dioxygenase reductase (AntC) | Carbon compound catabolism |
| PA3478* | -11.93 | 0.00 | Rhamnosyltransferase chain B (RhlB) | Secreted Factors |
| PA1783 | -11.86 | 0.00 | Nitrate transporter (NasA) | Transport of small molecules |
| PA2682 | -11.14 | 0.31 | Hypothetical protein | Hypothetical protein |
| PA0812 | -9.80 | 0.31 | Hypothetical protein | Hypothetical protein |
| PA0810* | -8.50 | 0.00 | Probable haloacid dehalogenase | Carbon compound catabolism |
| PA1781 | -7.47 | 0.00 | Assimilatory nitrite reductase large subunit | Central intermediary metabolism |
| PA1130 | -5.81 | 0.00 | Rhamnosyltransferase 2 (RhlC) | Secreted Factors |
| PA1131 | -5.26 | 0.00 | Probable MFS transporter | Transport of small molecules |
| PA4862 | -5.07 | 0.52 | Probable ATP-binding component of ABC transporter | Transport of small molecules |
| PA2511 | -4.64 | 0.52 | Probable transcriptional regulator | Regulatory function |
| PA1524 | -4.42 | 0.00 | Xanthine dehydrogenase | Nucleotide biosynthesis and metabolism |
| PA1863 | -4.31 | 1.53 | Molybdate-binding periplasmic protein precursor ModA | Transport of small molecules |
| PA1864* | -3.97 | 1.09 | Probable transcriptional regulator | Regulatory function |
| PA1155 | -3.86 | 0.00 | Ribonucleoside reductase, small chain | Nucleotide biosynthesis and metabolism |
| PA0085* | -3.74 | 0.00 | Hypothetical protein | Hypothetical protein |
| PA5476 | -3.58 | 0.00 | Citrate transporter | Transport of small molecules |
| PA3724 | -3.57 | 0.00 | Elastase LasB | Secreted Factors |
| PA3860 | -3.57 | 0.52 | Probable AMP-binding enzyme | Putative enzymes |
| PA3080 | -3.55 | 1.66 | Probable ATP-binding component of ABC transporter | Transport of small molecules |
| PA2507 | -3.55 | 0.52 | Catechol 1,2-dioxygenase | Carbon compound catabolism |
| PA2508 | -3.52 | 0.80 | Muconolactone delta-isomerase | Carbon compound catabolism |
| PA3192 | -3.45 | 0.00 | Two-component response regulator GltR | Regulatory function |
| PA1156* | -3.38 | 0.31 | Ribonucleoside reductase, large chain | Nucleotide biosynthesis and metabolism |
| PA4861 | -3.36 | 0.70 | Probable ATP-binding component of ABC transporter | Transport of small molecules |
| PA0809 | -3.26 | 0.00 | Probable transporter | Transport of small molecules |
| PA4860 | -3.25 | 1.53 | Probable permease of ABC transporter | Transport of small molecules |
| PA4261 | -3.17 | 0.52 | 50S ribosomal protein L23 | Translation |
| PA3907 | -3.16 | 0.00 | Hypothetical protein | Hypothetical protein |
| PA4933* | -3.13 | 1.09 | Hypothetical protein | Hypothetical protein |
| PA0084 | -3.11 | 0.52 | Hypothetical protein | Hypothetical protein |
| PA3476 | -3.09 | 0.00 | Autoinducer synthesis protein RhlI | Adaptation, protection |
| PA3326 | -3.08 | 0.00 | Probable Clp-family ATP-dependent protease | Protein degradation |
| PA4243* | -3.06 | 1.09 | Secretion protein SecY | Protein secretion/export apparatus |
| PA1523 | -3.04 | 1.14 | Xanthine dehydrogenase | Nucleotide biosynthesis and metabolism |
| PA3193 | -3.03 | 0.00 | Glucokinase | Carbon compound catabolism |
| PA0326 | -2.95 | 0.00 | Probable ATP-binding component of ABC transporter | Transport of small molecules |
| PA3779 | -2.95 | 0.00 | Hypothetical protein | Hypothetical protein |
| PA4407* | -2.94 | 0.00 | Cell division protein FtsZ | Cell division |
| PA4868 | -2.93 | 0.00 | Urease alpha subunit | Central intermediary metabolism |
| PA1048* | -2.90 | 0.00 | Probable outer membrane protein | Transport of small molecules |
| PA2003* | -2.88 | 0.75 | 3-hydroxybutyrate dehydrogenase | Carbon compound catabolism |
| PA5117 | -2.83 | 0.52 | Regulatory protein TypA | Adaptation, protection |
| PA4695 | -2.83 | 1.53 | Acetolactate synthase isozyme III small subunit | Amino acid biosynthesis and metabolism |
| PA1289 | -2.82 | 0.31 | Hypothetical protein | Hypothetical protein |
| PA0175 | -2.76 | 0.52 | Probable chemotaxis protein methyltransferase | Adaptation, protection |
| PA0122 | -2.76 | 0.75 | Hypothetical protein | Hypothetical protein |
| PA3182 | -2.76 | 0.52 | Hypothetical protein | Hypothetical protein |
| PA4262 | -2.75 | 0.42 | 50S ribosomal protein L4 | Translation |
| PA4259 | -2.71 | 1.14 | 30S ribosomal protein S19 | Translation |
| PA4260* | -2.65 | 0.52 | 50S ribosomal protein L2 | Translation |
| PA0174 | -2.61 | 0.52 | Hypothetical protein | Hypothetical protein |
| PA1199 | -2.60 | 0.31 | Probable lipoprotein | Membrane proteins |
| PA4258 | -2.59 | 1.66 | 50S ribosomal protein L22 | Translation |
| PA2305 | -2.57 | 0.00 | Probable non-ribosomal peptide synthetase | Putative enzymes |
| PA0176 | -2.55 | 0.52 | Probable chemotaxis transducer | Adaptation, protection |
| PA4859 | -2.54 | 1.87 | Probable permease of ABC transporter | Transport of small molecules |
| PA1584 | -2.53 | 0.52 | Succinate dehydrogenase (B subunit) | Energy metabolism |
| PA2266 | -2.47 | 1.87 | Probable cytochrome c precursor | Carbon compound catabolism |
| PA4273 | -2.45 | 0.80 | 50S ribosomal protein L1 | Translation |
| PA4865 | -2.44 | 1.09 | Urease gamma subunit | Central intermediary metabolism |
| PA2302 | -2.43 | 0.52 | Probable non-ribosomal peptide synthetase | Putative enzymes |
| PA4244 | -2.40 | 0.52 | 50S ribosomal protein L15 | Translation |
| PA4408 | -2.39 | 0.00 | Cell division protein FtsA | Cell division |
| PA0813 | -2.38 | 0.52 | Hypothetical protein | Hypothetical protein |
| PA3181* | -2.37 | 0.00 | 2-keto-3-deoxy-6-phosphogluconate aldolase | Carbon compound catabolism |
| PA4694 | -2.36 | 3.71 | Ketol-acid reductoisomerase | Amino acid biosynthesis and metabolism |
| PA0173 | -2.36 | 0.70 | Probable methylesterase | Adaptation, protection |
| PA0814* | -2.35 | 0.93 | Hypothetical protein | Hypothetical protein |
| PA4245 | -2.35 | 0.52 | 50S ribosomal protein L30 | Translation |
| PA4686 | -2.34 | 0.31 | Hypothetical protein | Hypothetical protein |
| PA2303 | -2.32 | 0.00 | Hypothetical protein | Hypothetical protein |
| PA4182 | -2.32 | 0.31 | Hypothetical protein | Hypothetical protein |
| PA5453 | -2.28 | 2.90 | GDP-mannose 4,6-dehydratase | Cell wall / LPS / capsule |
| PA4266 | -2.27 | 1.53 | Elongation factor G | Translation |
| PA4740* | -2.27 | 0.52 | Polyribonucleotide nucleotidyltransferase | Transcription and RNA processing |
| PA5036 | -2.26 | 0.52 | Glutamate synthase large chain precursor | Amino acid biosynthesis and metabolism |
| PA4744 | -2.26 | 2.17 | Translation initiation factor IF-2 | Translation |
| PA2304 | -2.18 | 0.31 | Hypothetical protein | Hypothetical protein |
| PA0904 | -2.16 | 0.52 | Aspartate kinase alpha and beta chain | Amino acid biosynthesis and metabolism |
| PA4935 | -2.14 | 0.52 | 30S ribosomal protein S6 | Translation |
| PA0083 | -2.13 | 4.69 | Hypothetical protein | Hypothetical protein |
| PA0090 | -2.13 | 0.00 | Probable ClpA/B-type chaperone | Protein degradation |
| PA3906 | -2.13 | 0.00 | Hypothetical protein | Hypothetical protein |
| PA4263 | -2.12 | 0.00 | 50S ribosomal protein L3 | Translation |
| PA3743 | -2.10 | 0.00 | tRNA (guanine-N1)-methyltransferase | Transcription and RNA processing |
| PA1583 | -2.10 | 3.56 | Succinate dehydrogenase (A subunit) | Energy metabolism |
| PA4344 | -2.10 | 0.93 | Probable hydrolase | Putative enzymes |
| PA0147* | -2.08 | 4.69 | Probable oxidoreductase | Putative enzymes |
| PA1589 | -2.08 | 0.52 | Succinyl-CoA synthetase alpha chain | Energy metabolism |
| PA4181 | -2.06 | 1.66 | Hypothetical protein | Hypothetical protein |
| PA3194* | -2.06 | 2.17 | Phosphogluconate dehydratase | Carbon compound catabolism |
| PA5035 | -2.06 | 1.09 | Glutamate synthase small chain | Amino acid biosynthesis and metabolism |
| PA4416 | -2.06 | 0.00 | UDP-N-acetylmuramoylalanyl-D-glutamyl-2, 6-diaminopimelate--D-alanyl-D-alanyl ligase | Cell wall/ LPS / capsule |
| PA0411 | -2.06 | 0.31 | Twitching motility protein PilJ | Motility & Attachment; Chemotaxis |
| PA1337 | -2.05 | 0.75 | Glutaminase-asparaginase | Amino acid biosynthesis and metabolism |
| PA5056 | -2.04 | 0.52 | Poly(3-hydroxyalkanoic acid) synthase 1 | Central intermediary metabolism |
| PA2537 | -2.04 | 0.00 | Probable acyltransferase | Fatty acid and phospholipid metabolism |
| PA4246 | -2.03 | 0.31 | 30S ribosomal protein S5 | Translation |
| PA1588 | -2.02 | 0.75 | Succinyl-CoA synthetase beta chain | Energy metabolism |
| PA4272 | -2.01 | 4.69 | 50S ribosomal protein L10 | Translation |

Microarray data were analyzed by using SAM (Significance Analysis of Microarrays) software (15). The criterion of cutoff limitation as a fold change ≥2 or ≤0.5 and q-value ≤5% was used to select differential expression genes. * The promoter (−1 bp to −400 bp of the coding region) harbors a conserved gatacannGC (where n is any nucleotide) DNA sequence without mismatch or with no more than one mismatch.

**Table S4. Verification of microarray results by Real-Time RT-PCR^a^**

|  | △*bfmS* vs MPAO1 | | △*bfmRS* vs MPAO1 |
| --- | --- | --- | --- |
| Gene | Microarray | qRT-PCR | qRT-PCR |
| *PA4100* | 10.38 | 8.2 | 0.94 |
| *PA4103* | 634.58 | >5000 | 4 |
| *PA4107* | 552.59 | 1237.03 | 0.99 |
| *PA4108* | 2.66 | 2.37 | 1.28 |
| *ntrB* | 2.46 | 2.11 | 1.43 |
| *oprH* | 5.98 | 15.45 | 1.88 |
| *phoB* | 2.57 | 6.94 | 0.74 |
| *hmgA* | 5.52 | 2.06 | 1.12 |
| *rhlA* | -16.31 | -14.5 | 1.3 |
| *antA* | -16.56 | -14.25 | 2.14 |
| *nasA* | -11.86 | -6.08 | -1.87 |
| *rhlI* | -3.09 | -2.07 | 1.32 |

^a^The primers used for Real-Time RT-PCR are listed in Table S6. Relative expression levels (fold change) of interest genes were calculated by the relative quantification method (ΔΔCT) as previously described [[7](#_ENREF_7)].

Table S5. 41 promoters identified by consensus sequence search

| SeqID | Strand | Start^a^ | End^a^ | matching_seq^b^ |
| --- | --- | --- | --- | --- |
| NP_248850.1\|PA0160 | R | -247 | -238 | gaggGATACAGCGCatag |
| NP_249026.1\|PA0335 | D | -357 | -348 | tcgcGATACAGCGCctct |
| NP_249026.1\|PA0335 | D | -113 | -104 | ggagGATACAGGGCcggc |
| NP_249027.1\|ygdP | R | -100 | -91 | ggagGATACAGGGCcggc |
| NP_249028.1\|ptsP | R | -358 | -349 | tcgcGATACAGCGCctct |
| NP_249146.1\|dbpA | D | -100 | -91 | caaaGATACATTGCctgt |
| NP_249147.1\|PA0456 | R | -257 | -248 | caaaGATACATTGCctgt |
| NP_249169.1\|PA0478 | D | -148 | -139 | tggcGATACAGGGCgtcg |
| NP_249699.1\|bcp | R | -260 | -251 | gggcGATACACCGCgctg |
| NP_249880.1\|PA1189 | R | -395 | -386 | tgcgGATACAGCGCcgcc |
| NP_250069.1\|PA1378 | R | -271 | -262 | ttgcGATACAGCGCcagc |
| NP_250163.1\|PA1472 | R | -229 | -220 | gtgcGATACAGCGCttcc |
| NP_250353.1\|PA1662 | R | -388 | -379 | gttcGATACACTGCtcgg |
| NP_250404.1\|exsA | D | -299 | -290 | gattGATACATTGCctgc |
| NP_250602.1\|femI | R | -274 | -265 | cgtgGATACAGCGCgcga |
| NP_250646.1\|PA1956 | D | -279 | -270 | cagcGATACACCGCcagc |
| NP_250838.1\|PA2148 | D | -12 | -3 | gcgaGATACAACGCcg |
| NP_250924.1\|pslD | R | -164 | -155 | acgcGATACAGGGCaccg |
| NP_250959.1\|PA2269 | D | -297 | -288 | tggtGATACAGCGCgccg |
| NP_251186.1\|PA2496 | R | -370 | -361 | tggcGATACAGCGCgccg |
| NP_251223.1\|PA2533 | R | -257 | -248 | gaggGATACACGGCatgc |
| NP_251367.1\|PA2677 | R | -398 | -389 | gaaaGATACAGAGCac |
| NP_251530.1\|PA2840 | R | -124 | -115 | agcgGATACACAGCcaga |
| NP_251531.1\|PA2841 | D | -196 | -187 | agcgGATACACAGCcaga |
| NP_251566.1\|pyrF | R | -183 | -174 | tgcgGATACAGGGCgtac |
| NP_251833.1\|PA3143 | D | -247 | -238 | gtagGATACAAGGCgggt |
| NP_251834.1\|PA3144 | D | -109 | -100 | gtagGATACAAGGCgggt |
| NP_251836.1\|wbpK | R | -196 | -187 | ggcaGATACACCGCaatc |
| NP_252154.1\|PA3464 | R | -207 | -198 | cgctGATACAAGGCaccc |
| NP_252155.1\|PA3465 | D | -19 | -10 | cgctGATACAAGGCaccc |
| NP_252268.1\|PA3578 | R | -66 | -57 | gggcGATACAGTGCgatc |
| NP_252305.1\|PA3615 | D | -188 | -179 | acctGATACATGGCgagc |
| NP_252376.1\|adk | R | -115 | -106 | ccaaGATACACAGCgccc |
| NP_252790.1\|bfmR | D | -219 | -210 | ggccGATACATGGCcgta |
| NP_252790.1\|bfmR | D | -89 | -80 | tccgGATACAGTGCaata |
| NP_252790.1\|bfmR | D | -44 | -35 | cgcgGATACACTGCttgc |
| NP_252796.1\|PA4107 | R | -205 | -196 | catgGATACAAAGCgata |
| NP_252797.1\|PA4108 | D | -128 | -119 | catgGATACAAAGCgata |
| NP_252824.1\|PA4135 | R | -280 | -271 | ccggGATACAACGCcagc |
| NP_253112.1\|PA4422 | D | -41 | -32 | tcgcGATACAATGCcgcc |
| NP_253113.1\|PA4423 | R | -95 | -86 | tcgcGATACAATGCcgcc |
| NP_253746.1\|PA5059 | R | -364 | -355 | gagcGATACACCGCgtcc |
| NP_253765.1\|PA5078 | D | -382 | -373 | gacaGATACATGGCcttt |
| NP_254173.1\|PA5486 | R | -168 | -159 | gctcGATACAGCGCctgg |

^a^Upstream regions from the start codon; ^b^Uppercase letters indicate the potential BfmR binding targets (GATACAnnGC, where n is any nucleotide) located upstream (−1 bp to −400 bp) of the coding region of the *P. aeruginosa* PAO1 genome by using RAST (http://rsat.ulb.ac.be/rsat/). Additionally, there are 984 promoters (−1 bp to −400 bp of the coding region) harbor a conserved gatacannGC (where n is any nucleotide) DNA sequence with one mismatch.

**Table S6. Primers used in this study**

| Name | Oligonucleotide sequence (5' to 3') |
| --- | --- |
| BfmSupF | TTTGAATTCATCATGATCGGCGAGAAGG |
| BfmSupR | TTTTCTAGAGGTAGCGCTCGTAGAACTGC |
| BfmSdownF | TTTTCTAGAGATTCCCGCCGAGGAACTCG |
| BfmSdownR | TTTAAGCTTGGTTCTGGCTTGCTGTGAA |
| BfmRupF | TTTGAATTCCCGATCAACCAGGTGTTCTT |
| BfmRupR | TTTTCTAGAGTGATCGACATGCTCCATTG |
| BfmR(comp)Fwr | TTTAAGCTTCACGGGAGCCAGGCAATGGA |
| BfmR(comp)Rev | TCTGGATCCGATCAGCGCCAGGC |
| BfmS(comp)Fwr | GGTAAGCTTGCTGCCGGTGCGACT |
| BfmS(comp)Rev | TTTGGATCCTCCGCAGTGTGTCCGC |
| RhlR-OE-F | GGGAAGCTTGCCAATTCTGCTGTGATGC |
| RhlR-OE-R | GGTAAGCTTCAGATGAGACCCAGCGCC |
| BfmR(D55A)-F | GTGGACCTGATCGTCCTCGCCATCATGATGCCCGGCGAC |
| BfmR(D55A)-R | GTCGCCGGGCATCATGATGGCGAGGACGATCAGGTCCAC |
| PA4102L181P-F | ACCGCCATCGGTCCGCCGACCCGGCTGGTGAAG |
| PA4102L181P-R | CTTCACCAGCCGGGTCGGCGGACCGATGGCGGT |
| PA4102E376Q-F | GGGGATTCCCGCCGAGCAACTCGACGAGGTGCT |
| PA4102E376Q-R | AGCACCTCGTCGAGTTGCTCGGCGGGAATCCCC |
| R393H-F | GAAAACTCGCGCAATCACGACACCGGTGGCACC |
| R393H-R | GGTGCCACCGGTGTCGTGATTGCGCGAGTTTTC |
| bfmR-F | TTGGACACATATGGAGCATGTCGATCACATCCTG |
| bfmR-R | CACACTCGAGTCATGGATGGGCCTCGA |
| bfmR-F(EMSA) | ATGCCGTAGCGGTAGCTTT |
| bfmR-R(EMSA) | GTGATCGACATGCTCCATTG |
| rhlR-F(EMSA) | TGGTCCATAAGGGCGGGCGGTATC |
| rhlR-R(EMSA) | AAGCCTCCGTCATTCCTCAT |
| rhlI-F(EMSA) | CTGCGTTGCATGATCGAG |
| rhlI-R(EMSA) | ATGACCAAGTCCCCGTGTCGT |
| Acka-up-F | TGGGGTACCGCGAAGGCTTCCTTGCTGTC |
| Acka-up-R | TTGGGATCCCTCTGGTGGAAGGCGGTGTC |
| Pta-domn-F | GTTGGATCCGCGGTGAATCCCGATCCTTC |
| Pta-domn-R | GGGAAGCTTCGATCGCGAGCAACTCCACT |
| Mini-TC-F | CGGGATCCcggtcgctaccattaccagt |
| Mini-TC-R | CGGGATCCctggtgagtcaagggttggt |
| Acka-comp-F | TTTAAGCTTAACACCTGCGCCACCAACCG |
| Pta-comp-R | TTTGGATCCCGCCGTTCACATGGCACATCC |
| 6FAM-bfmR-R | 6FAM-GTGATCGACATGCTCCATTG |
| 6FAM-rhlR-R | 6FAM-AAGCCTCCGTCATTCCTCAT |
| rhlA-F(EMSA) | GTGACCCTCGAGTTCTCCAA |
| rhlA-R(EMSA) | TTCGAACAGGCAAACAGCTA |
| rhlC-F (EMSA) | GCTTGCCCTGGTATTCCTC |
| rhlC-R(EMSA) | TCTCCCGTAGGTCGAAGTTG |
| PMS402-bfmR-F | TTTCTCGAGATGCCGTAGCGGTAGCTTT |
| PMS402-bfmR-R | TTTGGATCCGTGATCGACATGCTCCATTG |
| pms402-rhlA-F | TTTCTCGAGGCAGATGCTCTTCCTGCAAT |
| pms402-rhlA-R | TTTGGATCCTTCGAACAGGCAAACAGCTA |
| pms402-rhlR-1F | TTTGGATCCTGGTCCATCCGGGCGGTATC |
| pms402-rhlR-R | TTTGGATCCAAGCCTCCGTCATTCCTCAT |
| pms402-rhlR(D1)F | AATTGTCACAACCGCACGCTTGCGGTAAAGCGGC |
| pms402-rhlR(D1)R | GCCGCTTTACCGCAAGCGTGCGGTTGTGACAATT |
| bfmRflag-F | GGTAAGCTTTGCAAGATGGGCCACGACCA |
| bfmRflag-R | TTTGGATCCTTACTTATCGTCGTCATCCTTGTAATCTGGATGGGCCTCGACCAG |
| RT-PA4100-F | ACGAGCTGCTGCCGTACT |
| RT-PA4100-R | CCTGGAAGAACACCTGGTTG |
| RT-PA4103-F | CTGATCCTGGCCTACTGGAA |
| RT-PA4103-R | TTGAAGAACGACAGGCAGAA |
| RT-PA4107-F | GATCACGATGGCAAGGTCTC |
| RT-PA4107-R | GCTATCGATGCTGTCGAACTC |
| RT-PA4108-F | TCTGGAAAAGAGGTGGCTTC |
| RT-PA4108-R | TCGATCCAGACCTCCTTCAC |
| RT-ntrB-F | GATACCCTGCACCGACTGTT |
| RT-ntrB-R | ATGTACTCCAGGCGCAGTTC |
| RT-oprH-F | CTCGACAAGGTGATCGACAA |
| RT-oprH-R | GCTGGTGTCGGAGATGTTCT |
| RT-phoB-F | CATCGTTGATGACGAAGCAC |
| RT-phoB-R | ATCCAGTCGAGCAGGATCAG |
| RT-hmgA-F | CGATGGAGCGTTGCTTCTAC |
| RT-hmgA-R | ATCTCCAGCGGTTCGACTT |
| RT-rhlA-F | GGCGCGAAAGTCTGTTGG |
| RT-rhlA-R | CCAACGCGCTCGACATG |
| RT-antA-F | CATGCCTGGTGCTACAAGAA |
| RT-antA-R | AAGCCCCTGTAGCTCTGGAT |
| RT-nasA-F | CCTGAGCTTCATGGTCTGGT |
| RT-nasA-R | GCTGTTGGGTGCTCAGTTG |
| RT-rhlI-F | GCAGCTGGCGATGAAGATATTC |
| RT-rhlI-R | CGAACGAAATAGCGCTCCAT |
| PA4102-EX-F | ttccatgggcCAGTTCTACGAGCGCTACC |
| PA4102-EX-R | ttggatccttaCCAGCCGGACAGCGGC |
| pms402-p4103-F | TTTCTCGAGacagggacaagctgtggaac |
| pms402-p4103-R | TTTGGATCCGAGAGGTTGGGACGGTCAT |
| p4103-mutation-F | CCTGAGGCGCCCGTACAGAGCATATATTAACCGCCCTCGGCGGACAC |
| p4103-mutation-R | GTCCGCCGAGGGCGGTTAATATATGCTCTGTACGGGCGCCTCAGGGG |
| p4103-F (EMSA) | GTCGACAATGCGCTGAAGT |
| p4103-R (EMSA) | GAGAGGTTGGGACGGTCAT |
| p4103-R-FAM | FAM-GAGAGGTTGGGACGGTCAT |
| PA4108-F | GATGTATCGGGGGTGTATCG |
| PA4108-R | TCGATCCAGACCTCCTT |
| pZE.05 | CCAGCTGGCAATTCCGA |
| pZE.06 | AATCATCACTTTCGGGAA |

**REFERENCES**

1. Jansons I, Touchie G, Sharp R, Almquist K, Farinha MA, et al. (1994) Deletion and transposon mutagenesis and sequence analysis of the pRO1600 OriR region found in the broad-host-range plasmids of the pQF series. Plasmid 31: 265-274.

2. Hoang TT, Karkhoff-Schweizer RR, Kutchma AJ, Schweizer HP (1998) A broad-host-range Flp-FRT recombination system for site-specific excision of chromosomally-located DNA sequences: application for isolation of unmarked Pseudomonas aeruginosa mutants. Gene 212: 77-86.

3. Becher A, Schweizer HP (2000) Integration-proficient Pseudomonas aeruginosa vectors for isolation of single-copy chromosomal lacZ and lux gene fusions. Biotechniques 29: 948-950, 952.

4. Duan K, Dammel C, Stein J, Rabin H, Surette MG (2003) Modulation of Pseudomonas aeruginosa gene expression by host microflora through interspecies communication. Mol Microbiol 50: 1477-1491.

5. Liang H, Duan J, Sibley CD, Surette MG, Duan K (2011) Identification of mutants with altered phenazine production in Pseudomonas aeruginosa. J Med Microbiol 60: 22-34.

6. Jacobs MA, Alwood A, Thaipisuttikul I, Spencer D, Haugen E, et al. (2003) Comprehensive transposon mutant library of Pseudomonas aeruginosa. Proc Natl Acad Sci U S A 100: 14339-14344.

7. Lan L, Chen W, Lai Y, Suo J, Kong Z, et al. (2004) Monitoring of gene expression profiles and isolation of candidate genes involved in pollination and fertilization in rice ( Oryza sativa L.) with a 10K cDNA microarray. Plant Mol Biol 54: 471-487.
